# Comparative genomic insights into adaptation, selection signatures, and population dynamics in indigenous Indian sheep and foreign breeds

**DOI:** 10.3389/fgene.2025.1621960

**Published:** 2025-08-21

**Authors:** Malarmathi Muthusamy, Oludayo Michael Akinsola, Pritam Pal, Chitra Ramasamy, Saravanan Ramasamy, Abdulraheem Arome Musa, Aranganoor Kannan Thiruvenkadan

**Affiliations:** ^1^ Department of Animal Genetics and Breeding, Veterinary College and Research Institute, TANUVAS, Namakkal, India; ^2^ Department of Theriogenology and Production, Faculty of Veterinary Medicine, University of Jos, Jos, Nigeria; ^3^ Animal Genetics and Breeding Division, Indian Council of Agricultural Research (ICAR)-National Dairy Research Institute (NDRI), Karnal, India; ^4^ Research Institute for Farm Animal Biology (FBN), Dummerstorf, Germany; ^5^ College of Poultry Production and Management, TANUVAS, Hosur, India

**Keywords:** indigenous sheep, genomic inbreeding, runs of homozygosity (ROH), selection signatures, polygenic adaptation, population structure, effective population size, livestock conservation genetics

## Abstract

**Background:**

India’s indigenous sheep breeds have evolved under extreme and diverse agro‐ecological pressures, yet the genomic basis of their resilience and local adaptation remains poorly understood.

**Method:**

This study combines genomic inbreeding estimates, runs of homozygosity (ROH), population structure analyses, and composite selection scans to investigate three native Indian breeds—Changthangi, Deccani, and Garole—within a panel of nine breeds that also includes populations from Africa (Ethiopian Menz), East and South Asia (Tibetan, Chinese Merino, Bangladesh Garole, Bangladesh East), and Europe (Suffolk).

**Results:**

ROH and heterozygosity estimates revealed strong contrasts: Bangladesh East sheep exhibited high genomic inbreeding (F_ROH_≈14.4%) and low observed heterozygosity (∼30.6%), whereas Deccani sheep showed low inbreeding (F_ROH_≈1.1%) and high observed heterozygosity (∼35.6%), consistent with broader gene flow and larger flock sizes. Changthangi and Garole showed moderate inbreeding and distinct ROH length profiles. Population structure analyses confirmed ecological clustering and gene flow shaped by geography and husbandry practices: high‐altitude breeds clustered together, while directional migration edges traced admixture from European Suffolk into Changthangi and from Chinese Merino into Ethiopian Menz. Historical effective population sizes showed sharp declines in most breeds, especially those under recent selection. Selection scans identified 118 significant genomic regions across breeds. In Changthangi, key pathways included purinergic signaling, thyrotropin-releasing hormone, and autophagy—consistent with cold and hypoxia adaptation. Deccani showed enrichment for immune adhesion and epidermal regeneration, reflecting parasite resistance and heat stress. Garole displayed signals for gap-junction communication and skeletal development, aligned with high fertility and compact stature.

**Conclusion:**

These findings reveal ecotype‐pecific adaptive nature shaped by polygenic selection, gene flow, and demography, offering actionable insights for sustainable smallholder breeding strategies.

## 1 Introduction

India sustains one of the world’s largest sheep populations, estimated at approximately 80.7 million head, producing more than 11% of the nation’s meat and nearly all of its wool (FAOSTAT, 2024; Singh, 2024). These flocks provide livelihoods to millions of rural households spread across extreme and heterogeneous agro-ecological zones, from the cold deserts of Ladakh in northern India to the saline marshlands of the Sundarbans in the east (Bhateshwar et al., 2022). Indigenous breeds adapt to these environments under traditional management practices that include nomadic pastoralism, smallholder subsistence, and occasional crossbreeding (Banerjee et al., 2011; Sridhar, 2017; Saravanan et al., 2021). Three ecotypes exemplify the range of selective pressures in India: Changthangi, Deccani, and Garole. Changthangi sheep are renowned for their smooth, fine wool—prized in the production of luxurious fabrics—and for their remarkable high-altitude adaptation in the Ladakh region, where persistent hypoxia and subzero temperatures demand exceptional metabolic and thermoregulatory efficiencies (Ganai et al., 2011; Khan et al., 2022). Deccani sheep inhabit the semi-arid Deccan Plateau, facing recurrent heat stress, fodder scarcity, and parasites, which selects for disease resistance and robust meat-producing traits (APDAI, 2015; Sridhar, 2017). Garole sheep thrive in the Sundarbans delta, where salinity levels fluctuate seasonally and environmental resources are limited, thus shaping selection for high fecundity, tolerance to salt-affected grazing, and low-input productivity (Banerjee et al., 2011; Dhar, 2011).

Although India ranks among the leading global producers of sheep and sheep-derived products, the genomic underpinnings of adaptation in these local populations remain incompletely understood. Landmark global analyses such as Kijas et al. (2012) have highlighted broad patterns of ovine diversity but offered limited resolution of how unique environments and gene flows shaped the genomes of Indian sheep specifically. More recent studies have identified signatures of selection in certain indigenous breeds (Ahmad et al., 2021; Saravanan et al., 2021), yet they often relied on narrower panels or single statistical methods such as integrated haplotype score (iHS) or cross-population extended haplotype homozygosity (XP-EHH), potentially overlooking subtle or polygenic selective pressures (Voight et al., 2006; Ma et al., 2015). In addition, historical evidence suggests that cross-border exchanges, facilitated by trade routes and migratory pastoralism, introduced key alleles for traits like disease tolerance and enhanced wool yield (Muigai and Hanotte, 2013). However, systematic genomic comparisons among Indian, neighboring Asian, and more distantly related foreign breeds—particularly in the context of inbreeding, effective population size shifts, and polygenic selection—have received less comprehensive attention.

To address these gaps, the present study expands both the breed panel and analytical toolkit. We incorporate three indigenous breeds (Changthangi, Deccani, and Garole) alongside six additional populations that represent a spectrum of global agro-ecological and breeding objectives: Bangladesh Garole and Bangladesh East for cross-border reference, Tibetan for parallel high-altitude adaptation, Chinese Merino for intensive wool selection, Ethiopian Menz for African highland production, and Suffolk for commercial meat traits. This broader sampling captures how diverse environments and breeding aims drive genetic variation, while enabling more direct inferences on whether historical gene flow from foreign or regional breeds shaped local adaptation in India (Ganai et al., 2011; Rinchen and Nazia, 2023).

Methodologically, we integrate multiple genomic inbreeding metrics (Purcell et al., 2007; Yang et al., 2011; Purfield et al., 2012; Akinsola et al., 2024), reconstruct historical effective population sizes (Barbato et al., 2015), and quantify population differentiation ( $F_{ST}$ ) to clarify how demographic processes intersect with environmental selection (Pickrell and Pritchard, 2012). We further enhance single-population selection scans by adopting a de-correlated composite of multiple signals (DCMS) approach (Ma et al., 2015), which combines haplotype-based (e.g., iHS) and allele frequency–based (e.g., Tajima’s D) metrics to detect both recent and more subtle adaptive loci. This composite method is particularly relevant in sheep, where traits like cold tolerance, heat tolerance, and reproductive efficiency may stem from numerous genes of modest individual effect rather than a few large-effect loci (Voight et al., 2006; Ahmad et al., 2021; Saravanan et al., 2021).

We hypothesize that smaller or more geographically isolated breeds will exhibit pronounced inbreeding and more extensive runs of homozygosity, whereas lines experiencing broader gene flow or larger effective population sizes—such as Deccani—will display lower inbreeding. We further postulate that high-altitude breeds, notably Changthangi and Tibetan, will share genomic footprints linked to cold and hypoxia tolerance, reflected by low pairwise $F_{ST}$ and partially overlapping DCMS outliers. Finally, we anticipate that integrating haplotype- and frequency-based selection tests will reveal additional candidate loci tied to fecundity, thermotolerance, and immunological defense, underscoring the polygenic architecture of resilience in these small ruminants. By linking genomic signatures to specific ecological conditions and breeding practices, this study offers a more integrative view of how Indian sheep adapt to harsh environments and how external germplasm may be harnessed or managed to enhance productivity without eroding essential local adaptations.

## 2 Materials and methods

### 2.1 Data description

Genotypic data were obtained from the Web-Interfaced Next-generation Database for Genetic Diversity Exploration (WIDDE; Sempéré et al., 2015) a publicly accessible repository that requires no further ethical approvals. The dataset encompasses 240 individuals from nine sheep breeds, eight derived from a global ovine diversity survey (Kijas et al., 2012) and the ninth breed, Suffolk, from Rochus et al. (2018). All animals were genotyped on the Illumina Ovine SNP50 BeadChip (approximately 50,000 single nucleotide polymorphisms, SNPs), ensuring uniform coverage across the genome. The chromosomal locations are based on the OAR v3.1 assembly of the ovine genome.

Three indigenous Indian populations—Changthangi (CHA, n=29 ), Deccani (IDC, n=24 ), and Garole (GAR, n=26 )—formed the focal core of this investigation. The CHA sheep, from the cold desert of Ladakh, endure severe hypoxia and subzero temperatures (Ganai et al., 2011; Khan et al., 2022); IDC sheep, from the semi-arid Deccan Plateau, face recurrent heat stress and fodder scarcity (APDAI, 2015; Sridhar, 2017); and GAR sheep, from the Sundarbans delta, tolerate brackish, marshy conditions and have high fecundity (Banerjee et al., 2011; Dhar, 2011). To situate these within a broader comparative framework, six additional breeds representing diverse agro-ecological zones and breeding objectives were included: Bangladesh Garole (BGA, n = 24), Bangladesh East (BGE, n = 24), Tibetan (TIB, n = 37), Chinese Merino (CME, n = 23), Ethiopian Menz (EMZ, n = 34), and Suffolk (SUF, n = 19).

### 2.2 Genotypic quality control

All genotype files were processed in PLINK v1.9 (Purcell et al., 2007). The initial PED/MAP files were converted to binary BED/BIM/FAM format using the--make-bed command. Sample-level filtering excluded any individual with a call rate below 90% (i.e., --mind 0.10), which removed two Deccani samples and left a total of 238 individuals. At the SNP level, markers with a call rate below 95% (i.e., --geno 0.05) were removed, yielding 39,685 autosomal SNPs from the original 50K array. Sex chromosomes were excluded so that analyses focused solely on autosomal variation.

Because runs of homozygosity (ROH) analysis can benefit from maximal SNP density (Meyermans et al., 2020), no further pruning for minor allele frequency (MAF), Hardy–Weinberg equilibrium (HWE), or linkage disequilibrium (LD) was conducted at this stage. The final autosomal dataset averaged one SNP per ∼61 kb, covering more than 99% of the autosomal genome. Where specialized subsets were required (e.g., for ADMIXTURE, TreeMix, $F_{ST}$ , or selection scans), further LD pruning or MAF thresholds are described in the relevant subsections below.

### 2.3 Runs of homozygosity and inbreeding coefficients

ROH were identified using PLINK, guided by small-ruminant-oriented recommendations (Meyermans et al., 2020). We set a minimum ROH length of 1,000 kb (i.e., --homozyg-kb 1,000) and allowed gaps of up to 1,000 kb (i.e., --homozyg-gap 1,000) to reduce artificial fragmentation in genomic regions with moderate SNP spacing. Each sliding window of SNPs could not contain any heterozygous calls (--homozyg-window-het 0) and could tolerate one missing call (--homozyg-window-missing 1). A density threshold of one SNP per 150 kb (i.e., --homozyg-density 150) helped maintain consistency in coverage. We applied breed-specific minimum SNP thresholds (i.e., --homozyg-snp) using the L-parameter approach to account for variation in local linkage disequilibrium. The final ROH segments were sorted into categories of 1–5, 5–10, 10–15, 15–20, and >20 Mb in length to distinguish older from more recent inbreeding events (Curik et al., 2014).

Multiple genomic inbreeding coefficients were computed to capture different facets of autozygosity. $F_{ROH}$ was obtained as the proportion of total autosomal coverage in ROH, where the numerator is the total length of ROH per individual and the denominator is the total genomic length covered by SNPs (McQuillan et al., 2008). The variance-standardized genomic relationship coefficient ( $F_{GRM}$ ) was calculated using the--ibc function, allowing for the possibility of negative values if individuals exhibit higher heterozygosity than predicted by the reference allele frequencies (Purcell et al., 2007; Yang et al., 2011). $F_{\mathrm{HOM}}$ measured deviations in homozygosity relative to Hardy–Weinberg expectations (Purcell et al., 2007), and $F_{IS}$ was defined as ${1-H}_{O}/H_{E}$ , where $H_{O}$ and $H_{E}$ are the observed and expected heterozygosities, respectively. These metrics were collectively evaluated to reduce ambiguity that might arise from any single estimator (Purfield et al., 2012; Akinsola et al., 2024). Breed-specific means for ROH lengths and all inbreeding coefficients were compared by one-way analysis of variance, followed by Tukey–Kramer *post hoc* tests in the agricolae v1.3-7 R package (Felipe, 2023).

### 2.4 Population structure and demographic analyses

ADMIXTURE v1.3.0 (Alexander et al., 2009) was used to investigate genomic clusters. To limit the confounding impact of LD, we created a subset of unlinked SNPs in PLINK with a sliding window of 50 SNPs, a step of 5 SNPs, and an $r^{2}$ threshold of 0.2 (--indep-pairwise 50 5 0.2). Pairs of samples with an estimated relatedness $\hat{\rho }>0.25$ were removed to avoid biases from close kin. We ran ADMIXTURE for K clusters ranging from 1 to 7, recording cross-validation (CV) errors at each K. The optimal K was identified as the one with the lowest CV error. Individual ancestry coefficients were visualized in R as stacked bar plots.

Historical gene flow patterns were explored using TreeMix v1.13 (Pickrell and Pritchard, 2012). We removed SNPs with more than 5% missingness, then used the--freq option in PLINK to generate the required allele-count input. We tested models allowing 0 to 4 migration edges $\left(-m\;0\right.$ to −m 4 ), employing a block-jackknife of 500 SNPs $\left(-k\;500\right.$ ) to account for residual linkage. Suffolk served as the outgroup to root the tree, given its recognized genetic divergence as a commercial terminal-sire breed in many global surveys. The model fit was assessed using residual plots generated by TreeMix, which display the residual covariance between observed and model-predicted allele frequencies. A well-fitting model exhibits minimal and symmetrically distributed residuals. The four-migration-edge model minimized these residuals and was selected accordingly.

LD-based historical effective population size ( Ne ) was assessed using SNeP v1.1 (Barbato et al., 2015). Before analysis, a more stringent LD pruning was applied (a 100-SNP window, step size of 50 SNPs, and $r^{2}<0.1$ ). We modeled LD decay from 50 kb to 4 Mb using bin widths of 50 kb, with a mutation parameter α = 2 (Ohta and Kimura, 1971) and the Sved and Feldman (1973) mapping function. The 50 kb bin width was selected to ensure each window contained sufficient SNPs given the average SNP density (∼1 SNP/61 kb) of the ovine 50K SNP array. This approach balances resolution with statistical robustness, following practices from Barbato et al. (2015). The resulting Ne curves were traced from about 847 to 13 generations ago, acknowledging that smaller sample sizes in some breeds and reliance on a 50K SNP array can introduce uncertainties (Hayes et al., 2003; Corbin et al., 2012).

Population differentiation was assessed via the Weir and Cockerham (1984) $F_{ST}$ estimator in PLINK using--fst--within, setting negative $F_{ST}$ values to zero. Means for each pairwise breed comparison were assembled into a matrix and depicted as a heatmap in R (R Core Team, 2024). Negative estimates commonly arise from sampling variance or contrasting allele-frequency references (Purcell et al., 2007) and were not further interpreted as biological signals.

### 2.5 Selection scans and DCMS integration

Within each breed, we integrated multiple selection statistics into a DCMS framework (Ma et al., 2015). Haplotype-based metrics were computed on phased data produced by Beagle v5.4 (Browning et al., 2018; Browning et al., 2021).

The iHS was calculated in rehh v3.2.1 (Gautier et al., 2017) using chromosome-wise standardization, excluding SNPs with MAF <5%. The H12 statistic (Garud et al., 2015) was determined in 25-SNP windows with a 1-SNP step, while ZHp (Hofmeister et al., 2023) was calculated in 200 kb windows overlapping by 50%. The nucleotide diversity (π) and Tajima’s D were each estimated in 300 kb windows via VCFtools (Danecek et al., 2011), and windows containing fewer than 10 SNPs were excluded. iHS, H12, and π were rank-transformed for right-tailed p -values, whereas ZHp and Tajima’s D were left-tailed. To mitigate local LD effects, median smoothing was applied where appropriate.

For DCMS, we adopted 500 kb non-overlapping windows into which these five statistics were merged. The choice of a 500 kb window was based on the need to ensure adequate SNP representation per window for robust estimation of all five statistics. Given the SNP density of the ovine array (∼1 SNP per 61 kb), this window size provides a suitable balance between genomic resolution and statistical reliability. The sample covariance matrix among iHS, H12, ZHp, π, and Tajima’s D ensured that correlated signals did not artificially inflate composite scores. The DCMS analysis was performed in R using MINOTAUR v0.0.9000 (Verity et al., 2017), and each DCMS score was compared to a normal distribution parameterized by the sample mean and standard deviation. Benjamini–Hochberg adjustment was applied to control for multiple testing (Benjamini and Hochberg, 1995), and windows with q<0.05 were considered significant.

### 2.6 Candidate gene annotation and functional analysis

Significant DCMS windows were extended by ±500 kb and queried against the Ensembl Representational State Transfer (REST) application programming interface (Yates et al., 2020), mapped to OAR v3.1 Gene symbols and annotations were refined using BiomaRt v2.60.1 (Durinck et al., 2009). Putative functional roles were examined with the Database for Annotation, Visualization and Integrated Discovery (DAVID; Huang et al., 2009) at a nominal p<0.05 , focusing on Gene Ontology (GO) terms and Kyoto Encyclopedia of Genes and Genomes (KEGG) pathways linked to climate adaptation, parasite resistance, and reproductive traits. Any large gene sets underwent internal Benjamini–Hochberg correction in DAVID to reduce false positives. Finally, a network of enriched categories and gene clusters was visualized in the R package igraph v2.1.1 (Csardi and Tamas, 2006; Csárdi et al., 2025), with nodes colored by ontology domain and sized according to statistical significance or gene counts.

## 3 Results

### 3.1 Inbreeding, heterozygosity, and ROH

Table 1 summarizes $H_{O}$ (in %), ROH metrics, and multiple genomic inbreeding coefficients (in %) for the nine breeds. $H_{O}$ varied significantly among populations ( p<0.0001 ), ranging from 37.81 ± 0.42 in CME to 30.62 ± 0.75 in BGE. Within the indigenous Indian group, IDC showed the highest $H_{O}$ (35.63 ± 0.27), whereas CHA and GAR were at intermediate levels (34.48 ± 0.47 and 33.37 ± 0.60, respectively).

**TABLE 1:** Summary of runs of homozygosity (ROH) metrics and genomic inbreeding coefficients (mean ± se) in Indian and foreign sheep populations.

Metric	BGA	BGE	CHA	CME	EMZ	GAR	IDC	SUF	TIB	*p-*value
H_O_ (%)	33^d^ ± 0.58	30.62^e^ ± 0.75	34.48^bcd^ ± 0.47	37.81^a^ ± 0.42	34.51^bcd^ ± 0.26	33.37^d^ ± 0.6	35.63^abc^ ± 0.27	36.49^ab^ ± 0.14	34.11^cd^ ± 0.33	3.89 × 10^−21^
ROH (Mb)	32.79^de^ ± 2.69	29.96^de^ ± 2.87	14.52^b^ ± 1.94	17.74^bc^ ± 2.62	4.29^a^ ± 0.89	24.92^cd^ ± 2.8	2.95^a^ ± 0.7	37.47^e^ ± 1.24	15.43^b^ ± 1.79	2.96 × 10^−33^
ROH 1–5 Mb	15.92^d^ ± 1.27	11.25^c^ ± 1.06	5.24^ab^ ± 0.72	11.61^c^ ± 1.64	2.5^a^ ± 0.28	13.54^cd^ ± 0.97	1.45^a^ ± 0.31	24.79^e^ ± 1.23	6.59^b^ ± 0.62	6.78 × 10^−50^
ROH 5–10 Mb	10.21^c^ ± 1.01	8.33^c^ ± 0.92	4.48^b^ ± 0.61	3.65^ab^ ± 0.65	0.85^a^ ± 0.33	6.92^bc^ ± 1.01	0.68^a^ ± 0.21	10.26^c^ ± 0.67	4.59^b^ ± 0.65	1.11 × 10^−26^
ROH 10–15 Mb	3.5^c^ ± 0.61	3.58^c^ ± 0.6	1.9^bc^ ± 0.39	1.09^ab^ ± 0.28	0.24^a^ ± 0.0	1.81^ab^ ± 0.44	0.32^ab^ ± 0.23	1.32^ab^ ± 0.24	1.86^b^ ± 0.34	5.72 × 10^−11^
ROH 15–20 Mb	1.08^ab^ ± 0.23	2.04^b^ ± 0.41	0.83^a^ ± 0.21	0.61^a^ ± 0.22	0.21^a^ ± 0.11	1^a^ ± 0.28	0.27^a^ ± 0.15	0.84^a^ ± 0.21	0.76^a^ ± 0.18	2.7 × 10^−6^
ROH >20 Mb	2.08^a^ ± 0.7	4.75^b^ ± 1.11	2.07^a^ ± 0.65	0.78^a^ ± 0.23	0.5^a^ ± 0.32	1.65^a^ ± 0.58	0.23^a^ ± 0.16	0.26^a^ ± 0.13	1.62^a^ ± 0.35	8.19 × 10^−7^
ROH genome coverage (Mb)	256.7^cd^ ± 36	349.8^d^ ± 55.15	160.6^bc^ ± 31.78	109.49^ab^ ± 20.32	39.02^a^ ± 15.86	187.26^bc^ ± 36.12	25.48^a^ ± 10.53	188.81^bc^ ± 5.6	141.98^abc^ ± 20.44	9.32 × 10^−14^
$F_{ROH}$ (%)	10.56^cd^ ± 1.48	14.39^d^ ± 2.27	6.61^bc^ ± 1.31	4.5^ab^ ± 0.84	1.61^a^ ± 0.65	7.7^bc^ ± 1.49	1.05^a^ ± 0.43	7.77^bc^ ± 0.23	5.84^abc^ ± 0.84	9.37 × 10^−14^
$F_{GRM}$ (%)	−9.38^ab^ ± 1.81	−2.06^ab^ ± 2.84	0.28^b^ ± 2.33	−6.69^a^b ± 1.59	−6.82^ab^ ± 0.66	−12.85^a^ ± 5.95	−7.7^ab^ ± 1.29	−9.94^ab^ ± 0.79	−1.58^b^ ± 0.94	2.18 × 10^−3^
$F_{\mathrm{HOM}}$ (%)	3.62^ab^ ± 1.68	9.72^b^ ± 2	3.85^ab^ ± 1.51	−3.76^a^ ± 1.87	−0.2^a^ ± 0.9	0.58^a^ ± 3.69	−1.43^a^ ± 1.5	−2.55^a^ ± 0.64	3.37^ab^ ± 0.96	1.84 × 10^−5^
$F_{IS}$ (%)	0.05^c^ ± 0.02	0.12^d^ ± 0.02	0.04^bc^ ± 0.01	−0.04^a^ ± 0.01	0^abc^ ± 0.01	0^abc^ ± 0.02	−0.02^ab^ ± 0.01	−0.03^a^ ± 0	0.04^bc^ ± 0.01	1.7 × 10^−15^

All values are reported as mean ± standard error (SE). Different superscripts (a–e) in the same row indicate statistically significant differences (p < 0.05) based on Tukey–Kramer *post hoc* tests following one-way ANOVA. Abbreviations: BGA, Bangladesh Garole; BGE, Bangladesh East; CHA, Changthangi; CME, Chinese merino; EMZ, Ethiopian menz; GAR, Indian Garole; IDC, Deccani; SUF, Suffolk; TIB, Tibetan. HO: Observed heterozygosity; ROH (Mb): Total runs of homozygosity length in Mb; ROH 1–5 Mb, 5–10 Mb, *etc.*,: ROH length categories; ROH Genome Coverage: Length of autosomes covered by ROH segments in each breed; $F_{ROH}$ : Inbreeding coefficient based on the fraction of the genome in ROH; $F_{GRM}$ : Variance-standardized genomic relationship inbreeding coefficient, reflecting overall allele sharing; $F_{\mathrm{HOM}}$ : Homozygosity-based inbreeding coefficient exceeding Hardy–Weinberg expectations; $F_{IS}$ : Inbreeding coefficient derived from the ratio of observed to expected heterozygosity.

Analyses of total ROH length indicated that SUF, BGA, and BGE had the greatest ROH coverage, whereas IDC and EMZ had lower ROH totals. A large proportion of BGE’s ROH consisted of segments over 20 Mb, contributing to its elevated $F_{ROH}$ (14.39 ± 2.27). By contrast, IDC exhibited the lowest $F_{ROH}$ (1.05 ± 0.43). CHA and GAR fell between these extremes, with $F_{ROH}$ values of 6.61% ± 1.31% and 7.70% ± 1.49%, respectively.

Negative or near-zero inbreeding estimates arose in certain metrics. In GAR, $F_{GRM}$ was −12.85% ± 5.95%, which may reflect sample-related allele-frequency effects or a relatively high proportion of heterozygous individuals compared to breed-wide reference frequencies. The standard error indicates that some estimates could overlap zero, suggesting minimal actual inbreeding for those individuals. SUF, CME, and IDC also showed near-zero or negative $F_{IS}$ , implying lower observed homozygosity than expected. Overall, BGE displayed the strongest homozygosity measures, while IDC and EMZ maintained relatively low ROH-based inbreeding. CHA and GAR showed intermediate patterns, each with distinct ROH length distributions.

### 3.2 Population structure and demographics

**FIGURE 1:**
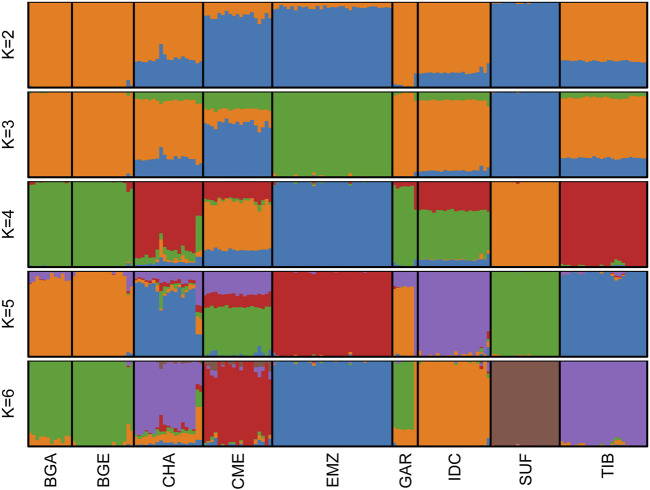
Population structure of Indian and foreign sheep breeds. ADMIXTURE plots showing genetic ancestry proportions at varying ancestral clusters (K = 2–6) for nine sheep populations. Each vertical bar represents a single individual, and the colors indicate ancestry fractions from inferred ancestral sources. Cross-validation analysis identified K = 6 as the optimal number of clusters (see Supplementary Figure S1). Breeds include Changthangi (CHA) from the high-altitude Ladakh region in northern India, Deccani (IDC) from the semi-arid Deccan Plateau in India, Indian Garole (GAR) from the Sundarbans delta in India, Bangladesh Garole (BGA) from southwestern Bangladesh, Bangladesh East (BGE) from eastern Bangladesh, Chinese Merino (CME) from northern China, Ethiopian Menz (EMZ) from the Ethiopian Highlands, Suffolk (SUF) from the United Kingdom, and Tibetan (TIB) from Himalayan regions of Asia.

Cross-validation identified K=6 as the best-supported model (Supplementary Figure S1). At K=2 , the high-altitude breeds CHA and TIB grouped together, while all remaining breeds formed the second cluster (Figure 1). Introducing a third component ( K=3 ) singled out the Bangladeshi populations (BGA, BGE). With K=4 , the foreign breed references began to separate: CME and SUF were almost entirely assigned to a new component, and EMZ already exceeded 90% segregation in its own. A fifth component ( K=5 ) detached IDC and GAR still shared a sizable proportion of Bangladeshi ancestry.

The fully resolved K=6 solution (Figure 1) showed that every breed except GAR was dominated by a single ancestry block. Mean proportions in that dominant component were TIB 97.7%, EMZ 93.6%, BGE 90.6%, BGA 90.5%, IDC 87.6%, SUF 89.2%, CME 81.7%, and CHA 68.0%. GAR retained 53.4% in its own component and 46.3% in the Bangladeshi component.

**FIGURE 2:**
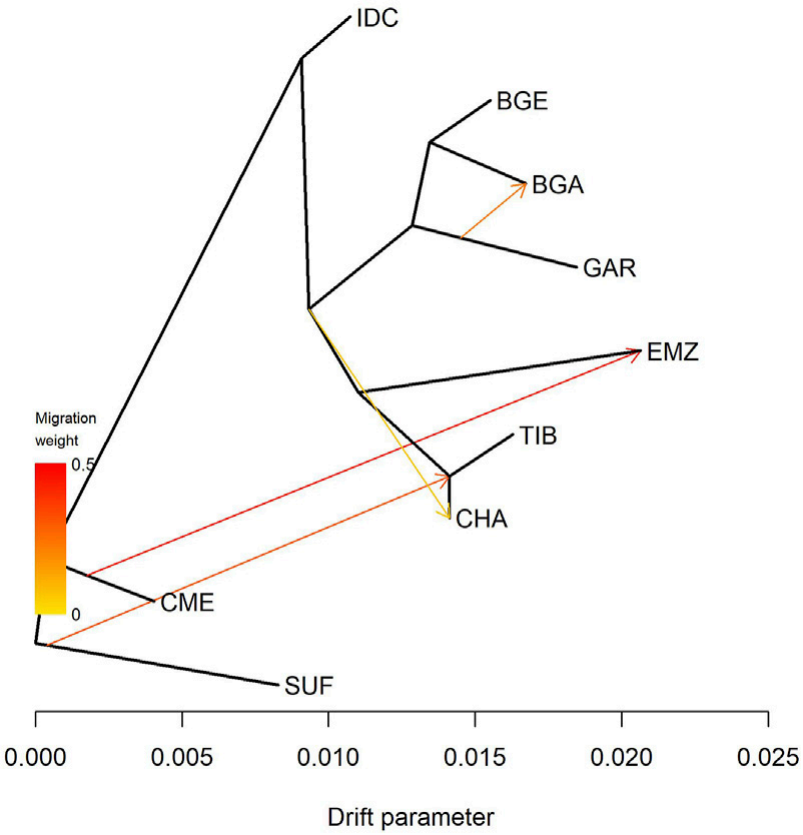
Maximum-likelihood phylogeny of Indian and foreign sheep breeds. This phylogeny was constructed using TreeMix with four migration edges that minimized residual errors (Supplementary File S1). Breeds include Changthangi (CHA) from the high-altitude Ladakh region in northern India, Deccani (IDC) from the semi-arid Deccan Plateau in India, Indian Garole (GAR) from the Sundarbans delta in India, Bangladesh Garole (BGA) from southwestern Bangladesh, Bangladesh East (BGE) from eastern Bangladesh, Chinese Merino (CME) from northern China, Ethiopian Menz (EMZ) from the Ethiopian Highlands, Suffolk (SUF) from the United Kingdom, and Tibetan (TIB) from Himalayan regions of Asia.

TreeMix (Figure 2) supported these findings through a four-edge migration model that minimized residual errors (Supplementary File S1). IDC appeared on the deepest branch, with no incoming gene flow. Two high-weight edges connected the reference breeds: one from SUF to CHA and another from CME to EMZ. In South Asia, BGA, BGE, and GAR formed a close cluster; a medium-weight edge ran from GAR to BGA and a weaker edge extended from the same Garole cluster towards CHA.

**FIGURE 3:**
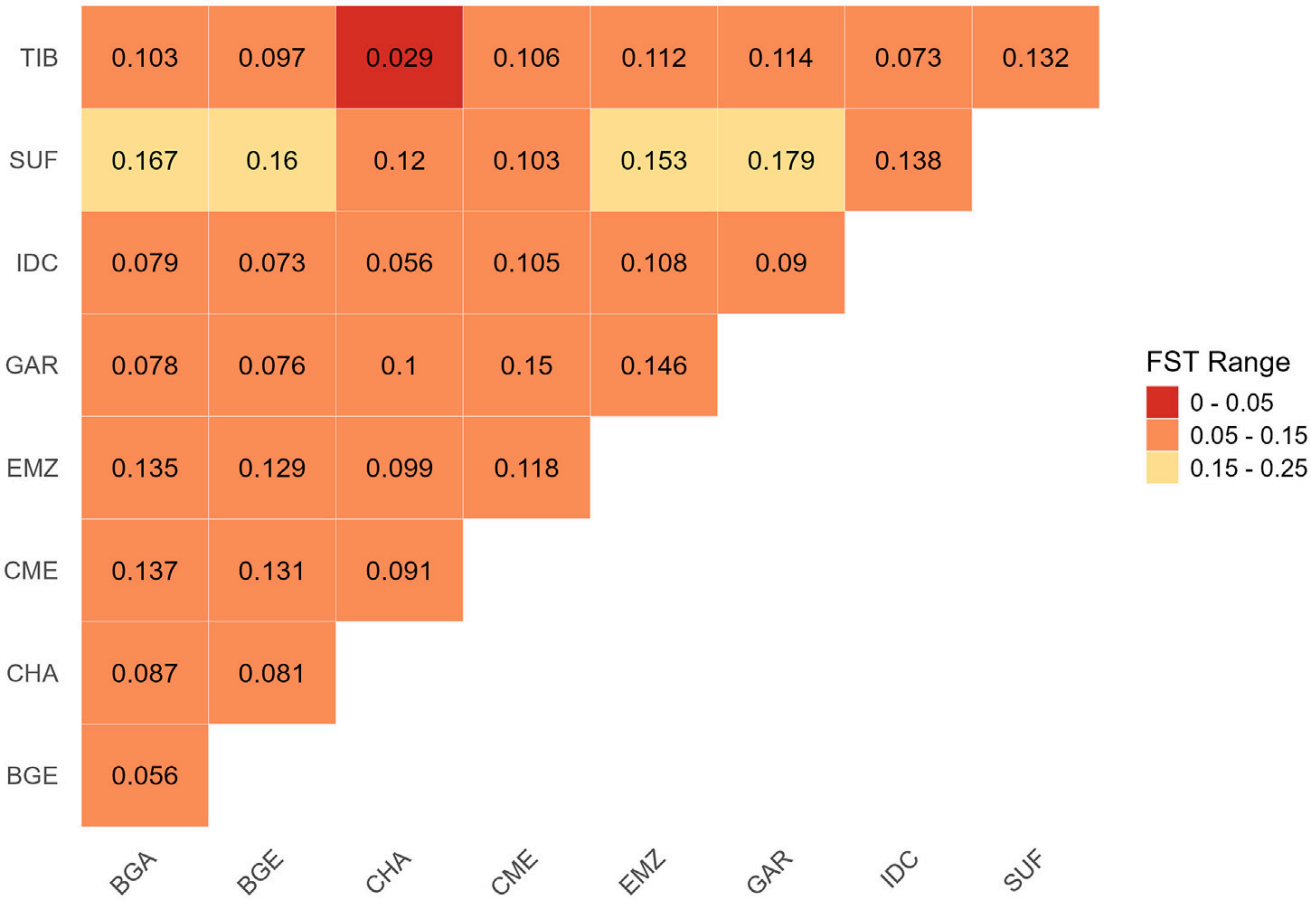
Pairwise genetic differentiation ( $F_{ST}$ ) heatmap among Indian and foreign sheep breeds. Lower $F_{ST}$ values (red) indicate low differentiation, orange indicate moderate differentiation, and yellow indicate high differentiation. Breeds include Changthangi (CHA) from the high-altitude Ladakh region in northern India, Deccani (IDC) from the semi-arid Deccan Plateau in India, Indian Garole (GAR) from the Sundarbans delta in India, Bangladesh Garole (BGA) from southwestern Bangladesh, Bangladesh East (BGE) from eastern Bangladesh, Chinese Merino (CME) from northern China, Ethiopian Menz (EMZ) from the Ethiopian Highlands, Suffolk (SUF) from the United Kingdom, and Tibetan (TIB) from Himalayan regions of Asia.

Pairwise $F_{ST}$ values (Figure 3) ranged from 0.029 (TIB vs. CHA) to 0.179 (SUF vs. GAR). Within India, CHA and IDC were the least differentiated ( $F_{ST}$ = 0.056), whereas CHA and GAR had a higher value (0.1). BGA and BGE showed moderate similarity ( $F_{ST}$ = 0.056). Other pairs, including BGE vs. CHA (0.081) and CME vs. IDC (0.105), fell in intermediate ranges. These patterns generally mirrored ADMIXTURE and TreeMix, highlighting lower genetic distance among ecologically similar breeds and higher divergence between populations adapted to contrasting environments or selected under distinct breeding objectives.

**FIGURE 4:**
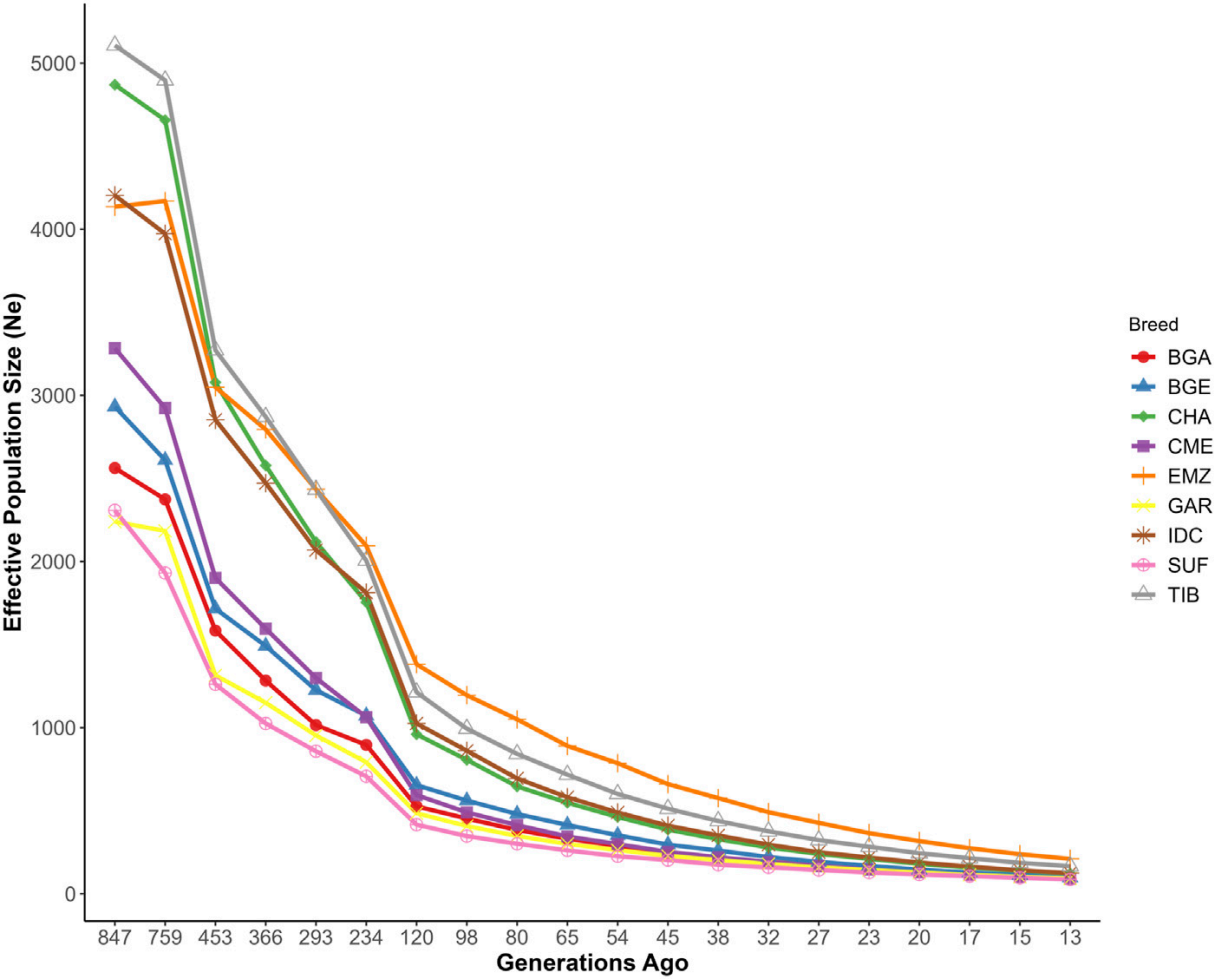
Historical trends in effective population size of Indian and foreign sheep breeds. Breeds include Changthangi (CHA) from the high-altitude Ladakh region in northern India, Deccani (IDC) from the semi-arid Deccan Plateau in India, Indian Garole (GAR) from the Sundarbans delta in India, Bangladesh Garole (BGA) from southwestern Bangladesh, Bangladesh East (BGE) from eastern Bangladesh, Chinese Merino (CME) from northern China, Ethiopian Menz (EMZ) from the Ethiopian Highlands, Suffolk (SUF) from the United Kingdom, and Tibetan (TIB) from Himalayan regions of Asia.

Estimates of historical $N_{e}$ (Figure 4) spanned 847 to 13 generations ago, revealing varying degrees of contraction or stability in each breed. At ∼847 generations, CHA, IDC, and TIB exceeded 4,000, while GAR stood at 2,240. BGA, BGE, and SUF were intermediate (2,563, 2,932, and 2,308, respectively). By ∼120 generations, CHA was ∼960, IDC 1,025, and GAR 485. Near the most recent time point (∼13 generations), IDC (124) and CHA (122) both remained above 100, while GAR dropped to 92. BGA and BGE dipped below 100, and SUF and CME were at 86 and 91, respectively. Because SNeP calculations can be sensitive to sample size and SNP density, these plots primarily provide a relative sense of demographic trends rather than absolute population sizes.

### 3.3 Selection signatures identified by DCMS

**FIGURE 5:**
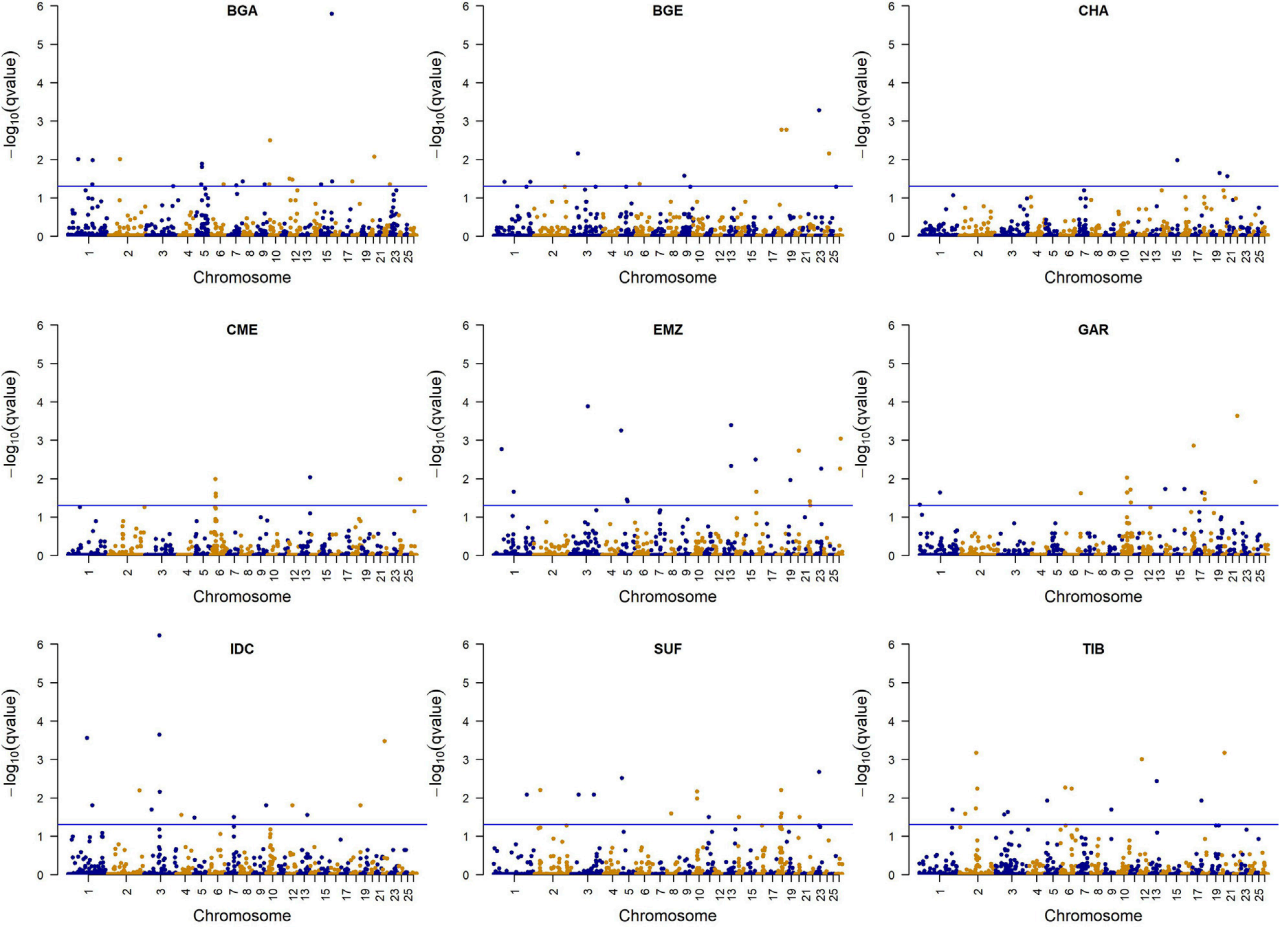
Manhattan plots show selection signals detected using the decorrelated composite of multiple signals for sheep breeds. The horizontal blue lines represent the false discovery rate threshold at q = 0.05. The points represent windows with varying statistical significance. Breeds include Changthangi (CHA) from the high-altitude Ladakh region in northern India, Deccani (IDC) from the semi-arid Deccan Plateau in India, Indian Garole (GAR) from the Sundarbans delta in India, Bangladesh Garole (BGA) from southwestern Bangladesh, Bangladesh East (BGE) from eastern Bangladesh, Chinese Merino (CME) from northern China, Ethiopian Menz (EMZ) from the Ethiopian Highlands, Suffolk (SUF) from the United Kingdom, and Tibetan (TIB) from Himalayan regions of Asia.

The DCMS analysis integrated five within-population statistics (iHS, H12, ZHp, π, and Tajima’s D), each calculated in 500 kb non-overlapping windows (Figure 5; Supplementary Table S1). In total, 118 windows surpassed the significance threshold ( q<0.05 ) across nine breeds, spanning multiple chromosomes. Among the Indian breeds, IDC and GAR each had 16 outlier windows, whereas CHA showed 3. In the reference populations, BGA had 22, EMZ 17, SUF and TIB 15 each, BGE 9, and CME 5. Annotations for these regions (Supplementary Table S2) revealed 521 protein-coding genes, with the largest counts observed in BGA (103), SUF (91), and IDC (79). Nine genes were shared among multiple breeds, while the rest appeared in distinct populations.

**FIGURE 6:**
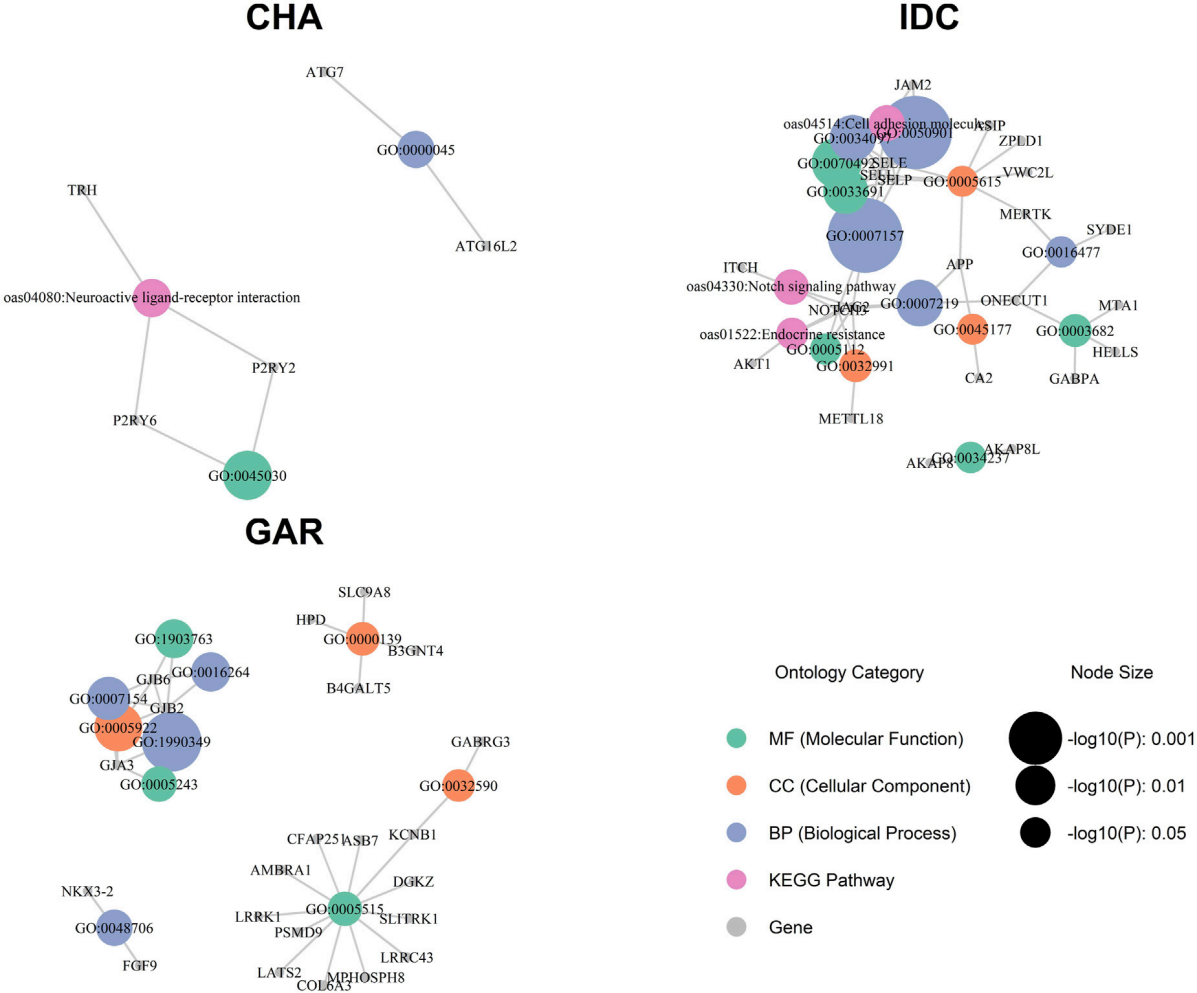
Gene ontology (GO) and KEGG pathway enrichment analysis for Indian sheep breeds: Changthangi (CHA), Deccani (IDC), and Indian Garole (GAR).

GO and KEGG pathway analysis (Supplementary Table S3) uncovered 73 enriched categories at p<0.05 , 10 of which remained significant after Benjamini–Hochberg correction ( q≤0.05 ). The Indian ecotypes exhibited characteristic enrichment patterns (Figure 6). CHA had three enriched terms at nominal levels, with GO:0045030 (“G protein–coupled UTP receptor activity”) meeting FDR significance (FDR = 0.0173) based on *P2RY6* and *P2RY2*; these two genes, along with *TRH*, also mapped to the KEGG pathway “Neuroactive ligand–receptor interaction” (oas04080; p=0.0144 ). An additional term, GO:0000045 (“Autophagosome assembly”), reached nominal significance ( p=0.0188 ) via *ATG16L2* and *ATG7* but did not remain after FDR correction. IDC displayed the most extensive profile among the Indian breeds, with three FDR-significant terms out of 16 nominally enriched categories, including GO:0007157 (“Heterophilic cell–cell adhesion via plasma membrane cell adhesion molecules”) and GO:0050901 (“Leukocyte tethering or rolling”). These were driven largely by *SELP*, *SELL*, *SELE*, *JAM2*, and *NOTCH3*, with additional contributors *APP* and *ITCH* detected in the same Notch-signalling window. A separate nominal enrichment for GO:0003682 (“chromatin binding”) involved *HELLS*, *MTA1*, *ONECUT1* and *GABPA*, suggesting possible selection on epigenetic regulators in IDC. GAR presented 10 nominal enrichments, five of which centered on gap-junction genes such as *GJB2*, *GJA3*, and *GJB6*; the strongest signal was GO:1990349 (“Gap junction-mediated intercellular transport”) at FDR = 0.0134. GAR also showed smaller-scale enrichment for embryonic skeletal development ( p=0.0183 ) involving *FGF9* and *NKX3-2*.

The foreign or reference breeds exhibited distinctive pathways (Supplementary Table S3; Supplementary File S2). BGA shared only one category with an Indian sheep, namely, “Neuroactive ligand–receptor interaction” (oas04080; p=0.0264 ), overlapping with CHA; within this pathway, supplementary hits were observed at GABRR3, GABRG3 and prolactin-releasing peptides PRP1/2. BGE displayed nominally enriched term, GO:1902176 (“Negative regulation of oxidative stress–induced intrinsic apoptotic signaling”; p=0.0067 ), driven by *BAG5* and *HSPB1*, which did not reach FDR significance. CME featured an FDR-significant KEGG category, oas04512 (“ECM–receptor interaction”; FDR = 0.0326), involving *SDC4*, *IBSP*, and *MEPE*. EMZ showed cytoskeletal and immune-related signals, including GO:0005856 (“Cytoskeleton”; FDR = 0.028), supported by *KITLG*, *TYK2*, and *ARHGAP26*, *PPP2R2B*, *TACC1* and *FRMD4B*. SUF had highly significant enrichment for desmosomal genes—GO:0030057 (“Desmosome”; FDR = 6.1 × 10^−8^)—and homophilic cell-adhesion loci, underpinned by *DSG1*, *DSC1*, *DSC3*, and *CDH15*. TIB returned nominal neuronal-structure enrichments (e.g., GO:0030424, “Axon,” p=0.0037 , driven by *NEFL*, *NEFM*, *SLC8A1*, and *SNCA*) that did not survive FDR.

Although some pathways overlapped partially among the foreign lines, only the “Neuroactive ligand–receptor interaction” category was shared across an Indian breed (CHA) and a foreign breed (BGA). Adhesion-related processes detected in IDC, gap-junction themes in GAR, and desmosomal signals in SUF did not appear in other populations, underscoring different selection histories and adaptive pressures among these breeds. All gene-level annotations and associated p-values are detailed in Supplementary Table S3.

## 4 Discussion

The integration of inbreeding metrics, population structure analyses, and composite selection scans provides a multifaceted view of how ecological pressures, demographic history, and human-mediated gene flow have shaped the genomes of Indian sheep—CHA, GAR and IDC—and related breeds. The patterns observed do not point to a single axis of differentiation—such as isolation or selection—but rather reflect the interplay of multiple forces acting at different intensities across breeds and landscapes. While some breeds display hallmarks of isolation and constrained diversity, others retain clear genomic evidence of admixture and broader mating networks. In parallel, the detected selection signatures are largely polygenic, with moderate-effect loci clustering within physiological and developmental pathways, reflecting adaptation to specific stressors like hypoxia, heat, parasites, or saline foraging. These insights add depth to earlier surveys of Indian sheep (Ahmad et al., 2021; Saravanan et al., 2021) by resolving finer-scale variation in genomic structure and by revealing the physiological systems most shaped by local environments and breeding regimes.

### 4.1 Inbreeding and heterozygosity

The combined analysis of genomic inbreeding metrics and heterozygosity revealed contrasting genetic profiles closely tied to breed-specific management practices and ecological contexts (Table 1). The BGE sheep exhibited exceptionally high genomic inbreeding, with approximately 14.39% of their autosomal genome encompassed by extensive ROH segments exceeding 20 Mb, alongside notably low observed heterozygosity (≈30.62%). Such genomic architecture typically arises in populations maintained in small, closed flock systems or under stringent selection practices. Comparable patterns are well-documented in intensively managed or isolated breeds, including Nguni and Blackhead Persian sheep (Dzomba et al., 2021), the miniature Ouessant breed from France (Ma et al., 2025), the improved Awassi line (Getachew et al., 2020), and isolated Mozambican river buffalo (Macciotta et al., 2021). Consistent with these examples, the exceptionally small flock sizes typical of indigenous sheep populations in Bangladesh, often limited to 5–30 individuals per household (Asaduzzaman et al., 2021), substantially elevate the risk of inbreeding accumulation and associated reductions in genetic diversity.

Conversely, IDC sheep demonstrated the lowest genomic inbreeding among the studied populations, with minimal ROH coverage ( $F_{ROH}$ ≈ 1.05%) and correspondingly high heterozygosity (≈35.63%). Such genetic signatures typify extensively managed breeds benefiting from periodic gene flow and crossbreeding events with Nellore sheep (APDAI, 2015). Similar genomic profiles have been reported in well-managed populations such as Small-tailed Han, Altay, Hu, and Bashibai sheep (Ma et al., 2025). IDC sheep, numbering approximately 1.4 million head and typically maintained in flocks of 25–200 animals, frequently receive genetic infusions from other breeds such as Garole, Bannur, or Awassi, thereby sustaining higher genetic diversity (Nimbkar et al., 2023). Such admixture practices, as documented in other extensively managed populations including farmed large white pigs (Shi et al., 2020) and cross-bred fat-tailed sheep (Kizilaslan et al., 2024), routinely yield low or even negative genomic inbreeding estimates.

CHA and GAR populations displayed intermediate levels of genomic autozygosity and heterozygosity. The moderate genomic inbreeding observed in CHA sheep ( $F_{ROH}$ ≈ 6.61%) likely reflects their geographic isolation in Ladakh’s high-altitude desert, tempered by periodic migratory pastoralism and limited gene inflow (Ganai et al., 2011; Lv et al., 2022). GAR sheep ( $F_{ROH}$ ≈ 7.70%), managed by smallholders in the ecologically distinct Sundarbans delta, also exhibited moderate genomic inbreeding consistent with partial population isolation, yet mitigated by cross-border genetic exchanges with Bangladeshi breeds (Banerjee et al., 2011). Notably, GAR sheep exhibited negative genomic relationship metrics ( $F_{GRM}$ ≈ −12.85%), indicative of excess heterozygosity relative to external allele frequency references. Such patterns are recognized statistical outcomes in populations with sporadic admixture, underscoring the importance of context-specific interpretation (Purfield et al., 2012; Shi et al., 2020).

These distinct genomic architecture highlight the strong interplay between flock size, genetic management strategies, and ecological setting in shaping breed-specific patterns of genetic diversity. Specifically, small, isolated flocks (e.g., BGE) risk rapid genomic erosion, while larger, periodically admixed populations (e.g., IDC) effectively maintain genetic health and resilience. Intermediate scenarios, as represented by CHA and GAR sheep, underscore the delicate balance between isolation-induced adaptive specialization and necessary genetic admixture to maintain long-term viability.

### 4.2 Population structure and demographic patterns

Population-structure analyses based on ADMIXTURE, TreeMix, and pairwise $F_{ST}$ estimates revealed distinct genetic clusters reflecting both ecological adaptation and historical patterns of gene flow. At optimal clustering ( K=6 ), each breed predominantly formed its own ancestral component. The IDC population demonstrated a notably homogeneous ancestry profile (>87% breed-specific ancestry), reflecting its extensive yet stable management across the Deccan Plateau. CHA, with approximately 68% of their genomic ancestry derived from a single breed-specific cluster, displayed moderate admixture consistent with historical interactions along Himalayan pastoral routes (Ganai et al., 2011; Ahmad et al., 2021). GAR, by contrast, retained only around 53% breed-specific ancestry, with a substantial genetic component (∼46%) shared with BGA. This admixture pattern aligns closely with documented cross-border exchanges in the Sundarbans region (Banerjee et al., 2011).

The TreeMix model provided complementary insights into directional gene flow patterns. IDC occupied the longest, edge-free branch, indicative of minimal recent admixture, consistent with documented selection sweeps associated with heat and drought tolerance (Saravanan et al., 2021). In contrast, the GAR population shared gene flow edges with Bangladeshi populations (BGA and BGE), supporting historical records of frequent trade in breeding rams between Indian and Bangladeshi Sundarbans communities (Banerjee et al., 2011). Additionally, moderate gene flow signals connecting SUF and CME to geographically distant breeds (e.g., CHA and EMZ) underscore historical crossbreeding efforts to introduce improved wool or meat traits, paralleling global sheep breeding trends (Kijas et al., 2012; Rochus et al., 2018; Da Silva et al., 2024).

$F_{ST}$ analyses reinforced the ADMIXTURE and TreeMix findings by quantitatively highlighting genetic divergence patterns. The lowest differentiation ( $F_{ST}$ ≈ 0.029) was observed between TIB and CHA sheep, affirming their shared genetic heritage and parallel high-altitude adaptations (Ganai et al., 2011). Conversely, maximal divergence ( $F_{ST}$ ≈ 0.179) between the specialized meat-producing SUF and the prolific, saline-tolerant GAR underscores strong ecological and selection-driven genetic differentiation (Banerjee et al., 2011; Kijas et al., 2012). Intermediate $F_{ST}$ values, such as those observed between CHA and IDC (≈0.056) or between BGE and CHA (≈0.081), reflect limited but measurable gene flow moderated by geographic and ecological barriers. Such gradations mirror gene-flow patterns reported in Ethiopian sheep across diverse agro-ecological zones (Edea et al., 2017).

Historical $N_{e}$ reconstructions highlighted dynamic demographic trajectories closely linked to breed-specific management and selection practices. IDC and CHA sheep historically maintained substantial $N_{e}$ (>4,000 individuals approximately 850 generations ago), indicating historically robust and diverse ancestral populations. However, both populations have experienced significant demographic contraction over recent generations (<130 individuals ∼13 generations ago), likely driven by increased management intensification, rangeland pressure, and restricted gene flow (Ganai et al., 2011; Sridhar, 2017). Conversely, GAR consistently exhibited smaller effective population sizes throughout its demographic history ( $N_{e}$ < 100 by recent generations), consistent with persistent smallholder management practices and habitat-induced isolation documented by Banerjee et al. (2011). Notably, commercial breeds such as CME and SUF displayed pronounced demographic contractions associated with intensive selective breeding regimes, echoing trends observed in other heavily selected livestock populations globally (Liu et al., 2017; Macciotta et al., 2021).

From a breeding management perspective, these demographic and structural insights underline two critical considerations. Firstly, breeds with stable admixture levels and historical gene inflow, exemplified by IDC, maintain sufficient genetic diversity and resilience to adaptively buffer against environmental stresses. Secondly, breeds with declining effective population sizes, such as GAR and CHA, require cautious management to balance ongoing selection pressures with controlled gene inflow strategies that preserve critical adaptive genetic variants (Ganai et al., 2011). Thus, strategic genetic improvement program tailored to each breed’s demographic history and ecological context emerges as pivotal for long-term sustainability and adaptive potential.

### 4.3 Selection signatures and polygenic adaptation

The DCMS framework applied here revealed nuanced selection signatures across the nine breeds, supporting the hypothesis that local adaptation in Indian sheep operates through polygenic architectures rather than single, hard sweeps. This composite approach, integrating haplotype- and frequency-based metrics, was particularly effective in uncovering subtle selection pressures, which might remain undetected by isolated statistics such as iHS alone—an issue highlighted in previous studies (Ahmad et al., 2021; Saravanan et al., 2021). A complete list of all enriched GO and KEGG terms, together with the underlying genes, is provided in Supplementary Table S3.

In CHA, the most compelling DCMS outlier regions included the purinergic receptors *P2RY2* and *P2RY6*, alongside *TRH* and autophagy-related genes *ATG7* and *ATG16L2*. These loci collectively reflect a multi-scale physiological response to high-altitude stress. Purinergic signalling plays a well-characterized role in endothelial vasodilation and oxygen delivery under hypoxic conditions (Erlinge and Burnstock, 2008; Burnstock and Pelleg, 2015), while *TRH* is integral to thermogenic control via thyroid hormone activation, a pathway upregulated under acute cold stress in mammals (Cabral et al., 2012). Meanwhile, the autophagy-related genes are known targets of HIF-1α-mediated hypoxia response, modulating cell survival through enhanced mitochondrial recycling (Chen et al., 2012). Although “Autophagosome assembly” did not survive FDR correction, the co-location of *ATG7*/*ATG16L2* with *P2RY2/6* and *TRH* inside the KEGG term “Neuroactive ligand–receptor interaction” indicates that CHA has tuned an integrated sensor-effector loop: purinergic receptors sense shear-stress ATP, *TRH* drives thyroidal heat, and mitophagy protects mitochondrial output, collectively potentially supporting ewe mobility at extremely low temperatures and lambing at high altitudes. The co-occurrence of these pathways suggests that CHA sheep have not adapted via single major-effect loci, but rather through subtle modulation of diverse pathways coordinating vasodilation, thermogenesis, and cellular protection in a hypoxic environment. Comparable high-altitude sweeps involving *ARHGEF17*, a mitotic-checkpoint regulator detected by XP-EHH in the same population (Ahmad et al., 2021), lend further support to the notion that multiple cell-survival pathways are co-opted in this breed.

IDC sheep exhibited a distinct immunological and thermotolerance profile. Notably, selectin genes (*SELP*, *SELL*, *SELE*), junctional adhesion molecule *JAM2*, and Notch pathway components (*NOTCH3*, *JAG2*, *APP*) were prominent within enriched categories linked to leukocyte trafficking and cell–cell communication. These molecules orchestrate the rapid mobilisation of neutrophils, a response critical under chronic parasite exposure—a well-known challenge in the semi-arid Deccan Plateau (McEver and Zhu, 2010; Vestweber, 2015; Sridhar, 2017). The inclusion of *APP* and the ubiquitin ligase ITCH—both regulators of γ-secretase turnover—implies that IDC has fine-tuned Notch signal duration rather than merely boosting receptor copy number, an adjustment likely advantageous for repeated tick infestation cycles. The associated Notch signalling further supports keratinocyte turnover and skin repair, traits that would be valuable under conditions of intense solar radiation and ectoparasite pressure (Bray, 2016). A supplementary finding, involving *AKAP8*/*AKAP8L*, points to scaffolded activation of the *PKA* cascade, a central axis in mammalian heat-shock response (Pidoux and Taskén, 2010).

In addition, nominal enrichment for “chromatin binding” (*HELLS*, *MTA1*, *ONECUT1*, *GABPA*) signals an epigenetic layer of immune regulation. *HELLS* encodes a chromatin helicase that remodels chromatin to facilitate DNA methylation by supporting *de novo* DNA methyltransferase activity (Zocchi et al., 2020) and is essential for ectopic proliferation in the developing retina, suggesting that selection on *HELLS* may help optimize chromatin control under intense solar irradiance. The functional breadth of IDC’s response is widened by *TBC1D12* and several RAB-family GTPases (*RAB17*/*21*/*24*/*28*) detected in additional nominal windows; these genes modulate vesicle traffic and cellular energy balance, traits previously associated with climate-mediated adaptation in sheep (Lv et al., 2014) and broadly relevant to intracellular adaptability and resilience in mammals (Homma et al., 2021). Together, these candidate loci and pathways suggest that IDC sheep have adapted to their environment through enhanced epithelial resilience, immune agility, and heat-responsive intracellular signalling.

GAR’s selective landscape is distinguished by its reproductive and morphological adaptations. The leading DCMS signal was driven by connexin genes (*GJB2*, *GJA3*, *GJB6*), which regulate intercellular metabolic cooperation in the ovary and epidermis (Goodenough and Paul, 2009). Connexins are essential for oocyte–granulosa cell communication, and their disruption impairs meiotic progression and ovulation, indicating that selection on these genes may underpin the breed’s high fecundity (Kidder and Mhawi, 2002). The same genes may also reinforce epidermal integrity, relevant in the Sundarbans’ saline and water-logged habitat. A second cluster of outliers included *NKX3-2* and *FGF9*—genes involved in skeletal patterning and chondrocyte proliferation, respectively (Hellemans et al., 2009; Ornitz and Itoh, 2015). These loci may support the compact, lightweight conformation characteristic of GAR sheep, facilitating mobility in swampy terrain. This constellation of traits—high fertility, skin integrity, and efficient locomotion—likely constitutes an integrated adaptive strategy tailored to the Sundarbans’ extreme and fluctuating conditions. Human studies indicate that mutations in *GJB2* and *GJB6* cause the majority of genetic cases of non-syndromic hearing loss (Chan and Chang, 2014), underscoring the conserved physiological importance of these gap-junction genes across mammals. Parallel signatures of selection on *GJB2* and *GJB6* have been detected in sheep and goats adapted to arid environments (Kim et al., 2016), possibly suggesting a shared evolutionary strategy among small ruminants in response to harsh ecological conditions. Another distinct genomic region under selection in Garole sheep includes *RNF17* and *PARP4*: *RNF17* is associated with germ-cell development, potentially enhancing reproductive resilience, whereas *PARP4* plays a critical role in DNA strand-break detection and repair and is notably expressed in sheep adipose tissue (Jean et al., 1999), implicating a role in oxidative stress resilience, possibly related to environmental challenges such as saline inundation. In the comparative reference populations, additional breed-specific adaptations were evident. The Ganges delta breed BGA showed signals in the neuroactive ligand–receptor pathway, particularly involving hypoxia-responsive receptors *P2RX3* and *APLNR*, which may regulate cardiorespiratory responses and vascular perfusion during oxygen deprivation. The *GABRR3*, *GABRG3*, and prolactin-releasing peptide signals suggest potential neuroendocrine modulation of water–salt balance, possibly complementing the *P2X3*–apelin perfusion system under prolonged submersion scenarios. These signals likely reflect physiological responses to the periodic flooding and high humidity that typify deltaic ecosystems. In BGE, stress-related genes *BAG5* and *HSPB1* were associated with antioxidant and anti-apoptotic responses, a plausible adaptation to oxidative stress induced by seasonal heat and waterlogging (Kalia et al., 2004; Webster et al., 2019). The same genomic region also contains *EXT1*, which encodes a glycosyltransferase essential for heparan sulfate biosynthesis and is implicated in tissue morphogenesis and developmental regulation (Okada et al., 2010). Its role in extracellular matrix formation and signaling could plausibly influence follicle morphogenesis and wool fiber diameter, potentially providing a link between flood-plain nutrition and fleece quality.

CME, shaped by decades of selection for wool traits, showed FDR-significant enrichment for ECM–receptor interaction pathways involving *SDC4*, *IBSP*, and *MEPE*. These genes contribute to matrix mineralisation and skin-follicle anchoring (Bouleftour et al., 2014; Carneiro et al., 2014), potentially supporting high wool density and structural robustness. SUF presented a highly significant signal for the desmosome complex, with enriched cadherins (*DSG1–4*, *DSC1–3*, *CDH15*) known to support epidermal cohesion under shear stress and contribute to muscle fibre integrity—attributes critical for a fast-growing meat breed managed under intensive systems (Yin and Green, 2004; Garrod and Chidgey, 2008).

EMZ, from the Ethiopian highlands, demonstrated polygenic enrichment for cytoskeletal and antioxidant genes, notably *DNAH9* and *MSRB3*. *DNAH9* encodes dynein chains crucial for mucociliary clearance, while MSRB3 protects against hypoxia-induced oxidative stress (Takeuchi et al., 2021; Chandran and Binninger, 2024; Seifu et al., 2024). Additional partners (*PPP2R2B*, *TACC1*, *FRMD4B*) strengthen microtubule stability, further potentially supporting high-altitude endurance. These findings align with earlier reports of altitude-driven adaptation in sheep (Wei et al., 2016). In TIB, while no FDR-significant hits were found, nominal enrichment for axonal structure genes (*NEFL*, *NEFM*, *SLC8A1*, *SNCA*) implies possible selection on peripheral nerve conductivity under cold-stress conditions, consistent with the breed’s highland origins (Yuan et al., 2017). Together with signatures of selection at *ATP12A*, a gene implicated in trophectoderm development and possibly linked to placental efficiency in cattle (Wei et al., 2017), these loci suggest coordinated selection on reproductive and metabolic pathways in sheep inhabiting East African highland environments. Altogether, the DCMS scan across these breeds elucidates a common theme: adaptive traits in sheep are not governed by singular, easily identifiable loci but emerge from small-effect variants distributed across multiple physiological pathways. This finding reinforces the utility of composite methods in livestock genomics, particularly for dissecting complex traits such as cold tolerance, parasite resistance, and reproductive efficiency—traits critical for flock sustainability in marginal environments.

### 4.4 Complementarity with single-metric studies

The added value of our DCMS-based approach becomes clearer when compared directly with earlier genome-wide selection scans that relied on single metrics. Saravanan et al. (2021), for example, used iHS alone to detect candidate sweeps in Indian sheep breeds. That study yielded important insights into loci under recent directional selection but was inherently biased toward detecting strong, ongoing sweeps with long haplotypes. In contrast, our composite method integrated iHS with four additional statistics—H12, ZHp, π, and Tajima’s D—allowing it to capture not only these canonical hard sweeps but also incomplete or diffuse signals consistent with soft sweeps or polygenic adaptation (Voight et al., 2006; Ma et al., 2015).

A direct juxtaposition of the two approaches (Supplementary Table S4) demonstrates both overlap and expansion. Nearly all of the high-confidence iHS signals reported by Saravanan et al. (2021) reappear in our DCMS analysis, reaffirming their biological relevance and underscoring the robustness of our pipeline. Notably, however, DCMS identifies an additional 19 outlier windows not captured by iHS alone. These novel windows are enriched for pathways involved in immune regulation (e.g., leukocyte adhesion, Notch signalling), thermotolerance (e.g., AKAP-mediated PKA activation), and tissue homeostasis (e.g., gap-junction and desmosomal integrity). Their biological plausibility is strengthened by earlier findings in animals exposed to analogous environmental challenges (Garrod and Chidgey, 2008; McEver and Zhu, 2010).

This layered discovery reflects the theoretical strengths of DCMS. While iHS performs well under assumptions of long, unbroken haplotypes rising rapidly in frequency, it is less effective at capturing older selection events or signals arising from subtle shifts in allele frequency. ZHp and π, for instance, are particularly sensitive to reductions in heterozygosity due to long-term selection but may miss more recent signals unless paired with haplotype-based tests. Tajima’s D, meanwhile, is informative for identifying population-level deviations in allele-frequency spectrum caused by balancing or directional selection but lacks spatial resolution on its own. The H12 statistic excels at detecting soft sweeps from standing variation, especially when multiple haplotypes are under selection. By decorrelating these metrics and aggregating them in a single composite, DCMS balances their complementary strengths while mitigating redundant signals (Ma et al., 2015; Verity et al., 2017).

Our findings illustrate that composite tests are not simply additive but synergistic: they can detect functionally relevant genomic regions that remain invisible to any single approach. Particularly in livestock species like sheep, where complex traits such as reproductive performance, parasite resilience, or thermal adaptation arise from distributed genetic architectures, reliance on single metrics risks underestimating the scope and heterogeneity of adaptive evolution.

### 4.5 Implications for breeding, conservation, and rural livelihoods

The selection signatures revealed in this study are not just of academic interest; they point to ecotype-specific constellations of genes—adaptive nature—that underpin real-world fitness and productivity under marginal conditions. These multi-gene configurations offer a genomic blueprint for tailoring breeding strategies that preserve key local adaptations while selectively enhancing production traits.

For CHA, the ensemble of purinergic receptors (*P2RY2*, *P2RY6*), *TRH*, and autophagy-related genes (*ATG7*, *ATG16L2*) represents a cold-climate physiological toolkit: vasodilation ensures tissue oxygenation, *TRH* promotes thermogenesis, and autophagy protects cellular function during hypoxic stress. These adaptations may be critical for winter survival in the Ladakh highlands. In IDC sheep, signals linked to neutrophil tethering (*SELP*, *SELE*, *SELL*, JAM2), Notch-mediated epidermal homeostasis (*NOTCH3*), and AKAP-anchored heat-shock signaling suggest a strong selection for immune responsiveness, skin repair, and acute stress response—traits that are indispensable in semi-arid environments with high parasite loads and radiant heat. In GAR, the prominence of gap-junction genes (*GJB2*, *GJB6*, *GJA3*) alongside mild enrichment in skeletal morphogenesis genes (*FGF9*, *NKX3-2*) supports a profile oriented toward reproductive efficiency, physical resilience in flooded terrain, and a compact frame suited to low-input production.

Preserving these local adaptations requires careful management. Unlike hard sweeps, which often involve high-frequency haplotypes at a few loci, polygenic traits are vulnerable to erosion through indiscriminate crossbreeding. Introgression of commercial traits—e.g., Merino fleece density or Suffolk carcass yield—into these populations is not inherently detrimental, but must be accompanied by strategies to monitor and retain key indigenous haplotypes. Selective breeding programs that use low-density SNP panels centered on the 118 DCMS outlier windows could achieve this balance at low cost.

The inbreeding patterns observed offer additional management cues. Breeds like BGE, with elevated $F_{ROH}$ and low observed heterozygosity, appear at risk of inbreeding depression unless fresh genetics are introduced. The sharply contracted $N_{e}$ values in BGE and GAR, combined with their village-scale flock structures (often <30 animals; Asaduzzaman et al., 2021), highlight the urgency of implementing controlled mating schemes. In contrast, IDC demonstrates how broad mating networks and occasional crossbreeding with Garole, Bannur or Awassi sires support a healthy genomic profile. These findings support models in which gene flow is neither excessive nor absent, but managed—preserving variation without eroding adaptive identity.

Breeds with historically large but recently contracted effective sizes, such as CHA and IDC, may retain enough diversity to support future selection programs, provided that key adaptive blocks are not lost. Conversely, lines with small long-term $N_{e}$ , such as GAR or BGE, may benefit more from conservation-focused interventions than intensive selection. These differences suggest that national breeding policies should not adopt a one-size-fits-all model, but instead align strategies with each breed’s demographic history, adaptive profile, and economic role.

From a rural livelihoods perspective, these genomic insights translate into concrete benefits. Adaptive traits—cold resilience, parasite defense, fertility—directly affect flock survival and productivity, especially under low-input conditions where veterinary care and supplemental feeding are scarce. Genomic conservation of these traits can reduce reliance on external inputs and buffer smallholders against climatic or epidemiological shocks. Aligning these goals with India’s livestock-mission objectives may offer a pathway toward pro-poor, climate-smart genetic improvement.

## 5 Conclusion

This study provides a comprehensive comparative genomic analysis of indigenous Indian sheep and related breeds across South Asia, Africa, and Europe, integrating metrics of inbreeding, population structure, and composite selection signals. By uniting haplotype- and frequency-based statistics through DCMS, we captured both recent and diffuse polygenic sweeps, identifying candidate genes linked to thermoregulation, immune responsiveness, hypoxia tolerance, fecundity, and oxidative stress resilience. Our results highlight that Indian breeds are not genetically insular; rather, they are shaped by local ecological pressures as well as episodic gene flow—historical and ongoing. The study confirms that adaptive traits are governed by multiple genomic pathways and that preserving these traits will require targeted management strategies to balance productivity gains with genetic conservation. Future work incorporating high-resolution phenotypes and functional validation will be essential to translate these genomic signals into breeding indices that support climate-smart, smallholder-oriented livestock improvement.

## Data Availability

Publicly available datasets were analyzed in this study. This data can be found here: http://widde.toulouse.inra.fr/widde/widde/main.do?module=sheep.

## References

[B1] Ahmad S. F. Mehrotra A. Charles S. Ganai N. A. (2021). Analysis of selection signatures reveals important insights into the adaptability of high-altitude Indian sheep breed Changthangi. Gene 799, 145809. 10.1016/j.gene.2021.145809 34224833

[B2] Akinsola O. M. Musa A. A. Muansangi L. Singh S. P. Mukherjee S. Mukherjee A. (2024). Genomic insights into adaptation and inbreeding among Sub-Saharan African cattle from pastoral and agropastoral systems. Front. Genet. 15, 1430291. 10.3389/fgene.2024.1430291 39119582 PMC11306176

[B3] Alexander D. H. Novembre J. Lange K. (2009). Fast model-based estimation of ancestry in unrelated individuals. Genome Res. 19, 1655–1664. 10.1101/gr.094052.109 19648217 PMC2752134

[B4] APDAI (2015). Reviving Deccani sheep breed for climate resilience by wassan NGO - Issuu. Available online at: https://issuu.com/wassanngo/docs/reviving_deccani_sheep_breed_for_cl (Accessed February 27, 2025).

[B5] Asaduzzaman M. Alam M. G. Jha P. K. Bari F. (2021). On‐farm management, breeding practice and constraints between two sheep breeds in Bangladesh. J. Anim. Prod., 62 (1),15–24. Available online at: https://dergipark.org.tr/en/pub/hayuretim/issue/62749/767083 (Accessed April 14, 2025).

[B6] Banerjee S. Galloway S. M. Davis G. H. (2011). Distribution of prolific Garole sheep in West Bengal, India. Anim. Genet. Resour. 48, 29–35. 10.1017/s207863361100004x

[B7] Barbato M. Orozco-terWengel P. Tapio M. Bruford M. W. (2015). SNeP: a tool to estimate trends in recent effective population size trajectories using genome-wide SNP data. Front. Genet. 6, 109. 10.3389/fgene.2015.00109 25852748 PMC4367434

[B8] Benjamini Y. Hochberg Y. (1995). Controlling the false discovery rate: a practical and powerful approach to multiple testing. J. R. Stat. Soc. Ser. B Stat. Methodol. 57, 289–300. 10.1111/j.2517-6161.1995.tb02031.x

[B9] Bhateshwar V. Rai D. C. Datt M. Aparnna V. P. (2022). Current status of sheep farming in India. J. Livest. Sci. 13, 135. 10.33259/jlivestsci.2022.135-151

[B10] Bouleftour W. Boudiffa M. Wade-Gueye N. M. Bouët G. Cardelli M. Laroche N. (2014). Skeletal development of mice lacking Bone Sialoprotein (BSP) - impairment of long bone growth and progressive establishment of high trabecular bone mass. PLoS One 9, e95144. 10.1371/journal.pone.0095144 24816232 PMC4015893

[B11] Bray S. (2016). Notch signalling in context. Nat. Rev. Mol. Cell Biol. 17, 722–735. 10.1038/nrm.2016.94 27507209

[B12] Browning B. L. Zhou Y. Browning S. R. (2018). A one-penny imputed genome from next-generation reference panels. Am. J. Hum. Genet. 103, 338–348. 10.1016/j.ajhg.2018.07.015 30100085 PMC6128308

[B13] Browning B. L. Tian X. Zhou Y. Browning S. R. (2021). Fast two-stage phasing of large-scale sequence data. Am. J. Hum. Genet. 108, 1880–1890. 10.1016/j.ajhg.2021.08.005 34478634 PMC8551421

[B14] Burnstock G. Pelleg A. (2015). Cardiac purinergic signalling in health and disease. Purinergic Signal 11, 1–46. 10.1007/s11302-014-9436-1 25527177 PMC4336308

[B15] Cabral A. Valdivia S. Reynaldo M. Cyr N. E. Nillni E. A. Perello M. (2012). Short-term cold exposure activates TRH neurons exclusively in the hypothalamic paraventricular nucleus and raphe pallidus. Neurosci. Lett. 518, 86–91. 10.1016/j.neulet.2012.04.059 22580206

[B16] Carneiro B. R. Pernambuco Filho P. C. A. De Sousa Mesquita A. P. Santos Da Silva D. Pinhal M. A. S. Nader H. B. (2014). Acquisition of anoikis resistance up-regulates syndecan-4 expression in endothelial cells. PLoS One 9, e116001. 10.1371/journal.pone.0116001 25549223 PMC4280138

[B17] Chan D. K. Chang K. W. (2014). GJB2-associated hearing loss: systematic review of worldwide prevalence, genotype, and auditory phenotype. Laryngoscope 124, E34–E53. 10.1002/lary.24332 23900770

[B18] Chandran S. Binninger D. (2024). Role of oxidative stress, methionine oxidation and methionine sulfoxide reductases (MSR) in alzheimer’s disease. Antioxidants 13, 21. 10.3390/antiox13010021 PMC10812627 38275641

[B19] Chen B. Longtine M. S. Nelson D. M. (2012). Hypoxia induces autophagy in primary human trophoblasts. Endocrinology 153, 4946–4954. 10.1210/en.2012-1472 22878401 PMC3512007

[B20] Corbin L. J. Liu A. Y. H. Bishop S. C. Woolliams J. A. (2012). Estimation of historical effective population size using linkage disequilibria with marker data. J. Anim. Breed. Genet. 129, 257–270. 10.1111/j.1439-0388.2012.01003.x 22775258

[B21] Csardi G. Tamas N. (2006). The igraph software package for complex network research. Inter. J. Complex Syst. 1695. 1–9. Available online at: http://igraph.org/ (Accessed April14, 2025).

[B22] Csárdi G. Nepusz T. Traag V. Horvát S. Zanini F. Noom D. (2025). Igraph: network analysis and visualization in R. R package. version 2.1.1. Available online at: https://CRAN.R-project.org/package=igraph.

[B23] Curik I. Ferenčaković M. Sölkner J. (2014). Inbreeding and runs of homozygosity: a possible solution to an old problem. Livest. Sci. 166, 26–34. 10.1016/j.livsci.2014.05.034

[B24] Da Silva A. Ahbara A. Baazaoui I. Jemaa S. B. Cao Y. Ciani E. (2024). History and genetic diversity of African sheep: contrasting phenotypic and genomic diversity. Anim. Genet. 56, e13488. 10.1111/age.13488 39561986 PMC11666867

[B25] Danecek P. Auton A. Abecasis G. Albers C. A. Banks E. DePristo M. A. (2011). The variant call format and VCFtools. Bioinformatics 27, 2156–2158. 10.1093/bioinformatics/btr330 21653522 PMC3137218

[B26] Dhar S. (2011). Impact of climate change on the salinity situation of the Piyali River, sundarbans, India. J. Water Resour. Prot. 03, 495–503. 10.4236/jwarp.2011.37059

[B27] Durinck S. Spellman P. Birney E. Huber W. (2009). Mapping identifiers for the integration of genomic datasets with the R/Bioconductor package biomaRt. Nat. Protoc. 4, 1184–1191. 10.1038/nprot.2009.97 19617889 PMC3159387

[B28] Dzomba E. F. Chimonyo M. Pierneef R. Muchadeyi F. C. (2021). Runs of homozygosity analysis of South African sheep breeds from various production systems investigated using OvineSNP50k data. BMC Genomics 22, 7. 10.1186/s12864-020-07314-2 33407115 PMC7788743

[B29] Edea Z. Dessie T. Dadi H. Do K. T. Kim K. S. (2017). Genetic diversity and population structure of Ethiopian sheep populations revealed by high‐density SNP markers. Front. Genet. 8, 218. 10.3389/fgene.2017.00218 29312441 PMC5744078

[B30] Erlinge D. Burnstock G. (2008). P2 receptors in cardiovascular regulation and disease. Purinergic Signal 4, 1–20. 10.1007/s11302-007-9078-7 PMC2245998 18368530

[B31] FAOSTAT (2024). Food and agriculture database. Food Agric. Organ. United Nations. Available online at: https://www.fao.org/faostat/en/#data (Accessed April 14, 2025).

[B32] Felipe M. (2023). Agricolae: statistical procedures for agricultural research. R packag. version 1.3-7. Available online at: https://cran.r-project.org/package=agricolae.

[B33] Ganai T. A. Misra S. S. Sheikh F. D. (2011). Description of Changthangi sheep of Ladakh. Indian J. Small Ruminants 17, 32–40. Available online at: https://www.cabidigitallibrary.org/doi/pdf/10.5555/20113188872 (Accessed April 14, 2025).

[B34] Garrod D. Chidgey M. (2008). Desmosome structure, composition and function. Biochim. Biophys. Acta - Biomembr. 1778, 572–587. 10.1016/j.bbamem.2007.07.014 17854763

[B35] Garud N. R. Messer P. W. Buzbas E. O. Petrov D. A. (2015). Recent selective sweeps in north American Drosophila melanogaster show signatures of soft sweeps. PLoS Genet. 11, e1005004–e1005032. 10.1371/journal.pgen.1005004 25706129 PMC4338236

[B36] Gautier M. Klassmann A. Vitalis R. (2017). Rehh 2.0: a reimplementation of the R package rehh to detect positive selection from haplotype structure. Mol. Ecol. Resour. 17, 78–90. 10.1111/1755-0998.12634 27863062

[B37] Getachew T. Haile A. Mészáros G. Rischkowsky B. Huson H. J. Gizaw S. (2020). Genetic diversity, population structure and runs of homozygosity in Ethiopian short fat-tailed and Awassi sheep breeds using genome-wide 50k SNP markers. Livest. Sci. 232, 103899. 10.1016/j.livsci.2019.103899

[B38] Goodenough D. A. Paul D. L. (2009). Gap junctions. Cold Spring Harb. Perspect. Biol. 1, a002576. 10.1101/cshperspect.a002576 20066080 PMC2742079

[B39] Hayes B. J. Visscher P. M. McPartlan H. C. Goddard M. E. (2003). Novel multilocus measure of linkage disequilibrium to estimate past effective population size. Genome Res. 13, 635–643. 10.1101/gr.387103 12654718 PMC430161

[B40] Hellemans J. Simon M. Dheedene A. Alanay Y. Mihci E. Rifai L. (2009). Homozygous inactivating mutations in the NKX3-2 gene result in Spondylo-Megaepiphyseal-Metaphyseal dysplasia. Am. J. Hum. Genet. 85, 916–922. 10.1016/j.ajhg.2009.11.005 20004766 PMC2790567

[B41] Hofmeister R. J. Ribeiro D. M. Rubinacci S. Delaneau O. (2023). Accurate rare variant phasing of whole-genome and whole-exome sequencing data in the UK Biobank. Nat. Genet. 55, 1243–1249. 10.1038/s41588-023-01415-w 37386248 PMC10335929

[B42] Homma Y. Hiragi S. Fukuda M. (2021). Rab family of small GTPases: an updated view on their regulation and functions. FEBS J. 288, 36–55. 10.1111/febs.15453 32542850 PMC7818423

[B43] Huang D. W. Sherman B. T. Lempicki R. A. (2009). Systematic and integrative analysis of large gene lists using DAVID bioinformatics resources. Nat. Protoc. 4, 44–57. 10.1038/nprot.2008.211 19131956

[B44] Jean L. Risler J. L. Nagase T. Coulouarn C. Nomura N. Salier J. P. (1999). The nuclear protein PH5P of the inter-α-inhibitor superfamily: a missing link between poly(ADP-ribose)polymerase and the inter-α-inhibitor family and a novel actor of DNA repair? FEBS Lett. 446, 6–8. 10.1016/S0014-5793(99)00173-8 10100603

[B45] Kalia S. K. Lee S. Smith P. D. Liu L. Crocker S. J. Thorarinsdottir T. E. (2004). BAG5 inhibits parkin and enhances dopaminergic neuron degeneration. Neuron 44, 931–945. 10.1016/j.neuron.2004.11.026 15603737

[B46] Khan N. N. Ganai N. Ahmad T. Shah R. (2022). Changthangi sheep: the pride of Ladakh. 1, 49–51. 10.5281/zenodo.6535189

[B47] Kidder G. M. Mhawi A. A. (2002). Gap junctions and ovarian folliculogenesis. Reproduction 123, 613–620. 10.1530/rep.0.1230613 12006089

[B48] Kijas J. W. Lenstra J. A. Hayes B. Boitard S. Neto L. R. Cristobal M. S. (2012). Genome-wide analysis of the world’s sheep breeds reveals high levels of historic mixture and strong recent selection. PLoS Biol. 10, e1001258. 10.1371/journal.pbio.1001258 22346734 PMC3274507

[B49] Kim E. S. Elbeltagy A. R. Aboul-Naga A. M. Rischkowsky B. Sayre B. Mwacharo J. M. (2016). Multiple genomic signatures of selection in goats and sheep indigenous to a hot arid environment. Heredity (Edinb) 116, 255–264. 10.1038/hdy.2015.94 26555032 PMC4806575

[B50] Kizilaslan M. Arzik Y. Behrem S. White S. N. Cinar M. U. (2024). Comparative genomic characterization of indigenous fat-tailed Akkaraman sheep with local and transboundary sheep breeds. Food Energy Secur 13, e508. 10.1002/fes3.508

[B51] Liu S. He S. Chen L. Li W. Di J. Liu M. (2017). Estimates of linkage disequilibrium and effective population sizes in Chinese Merino (Xinjiang type) sheep by genome-wide SNPs. Genes Genomics 39, 733–745. 10.1007/s13258-017-0539-2 28706593 PMC5486679

[B52] Lv F. H. Agha S. Kantanen J. Colli L. Stucki S. Kijas J. W. (2014). Adaptations to climate-mediated selective pressures in sheep. Mol. Biol. Evol. 31, 3324–3343. 10.1093/molbev/msu264 25249477 PMC4245822

[B53] Lv F. H. Cao Y. H. Liu G. J. Luo L. Y. Lu R. Liu M. J. (2022). Whole-genome resequencing of worldwide wild and domestic sheep elucidates genetic diversity, introgression, and agronomically important loci. Mol. Biol. Evol. 39, msab353. 10.1093/molbev/msab353 34893856 PMC8826587

[B54] Ma Y. Ding X. Qanbari S. Weigend S. Zhang Q. Simianer H. (2015). Properties of different selection signature statistics and a new strategy for combining them. Heredity (Edinb) 115, 426–436. 10.1038/hdy.2015.42 25990878 PMC4611237

[B55] Ma R. Liu J. Ma X. Yang J. (2025). Genome-wide runs of homozygosity reveal inbreeding levels and trait-associated candidate genes in diverse sheep breeds. Genes (Basel) 16, 316. 10.3390/genes16030316 40149467 PMC11942120

[B56] Macciotta N. P. P. Colli L. Cesarani A. Ajmone-Marsan P. Low W. Y. Tearle R. (2021). The distribution of runs of homozygosity in the genome of river and swamp buffaloes reveals a history of adaptation, migration and crossbred events. Genet. Sel. Evol. 53, 20. 10.1186/s12711-021-00616-3 33639853 PMC7912491

[B57] McEver R. P. Zhu C. (2010). Rolling cell adhesion. Annu. Rev. Cell Dev. Biol. 26, 363–396. 10.1146/annurev.cellbio.042308.113238 19575676 PMC3557855

[B58] McQuillan R. Leutenegger A.-L. Abdel-Rahman R. Franklin C. S. Pericic M. Barac-Lauc L. (2008). Runs of homozygosity in European populations. Am. J. Hum. Genet. 83, 658. 10.1016/j.ajhg.2008.10.009 PMC2556426 18760389

[B59] Meyermans R. Gorssen W. Buys N. Janssens S. (2020). How to study runs of homozygosity using plink? A guide for analyzing medium density snp data in livestock and pet species. BMC Genomics 21, 94. 10.1186/s12864-020-6463-x 31996125 PMC6990544

[B60] Muigai A. W. T. Hanotte O. (2013). The origin of African sheep: archaeological and genetic perspectives. Afr. Archaeol. Rev. 30, 39–50. 10.1007/s10437-013-9129-0 PMC4851118 27212780

[B61] Nimbkar C. Ghalsasi P. M. Walkden-Brown S. W. Van Der Werf J. H. J. (2023). FecB carrier Deccani cross bred ewes in Maharashtra, India have moderately higher litter sizes than non-carrier ewes. Proc. Assoc. Advmt. Anim. Breed. Genet. 25, 138–141. Available online at: https://hdl.handle.net/1959.11/56098 (Accessed April 14, 2025).

[B62] Ohta T. Kimura M. (1971). On the constancy of the evolutionary rate of cistrons. J. Mol. Evol. 1, 18–25. 10.1007/BF01659391 4377445

[B63] Okada M. Nadanaka S. Shoji N. Tamura J. I. Kitagawa H. (2010). Biosynthesis of heparan sulfate in EXT1-deficient cells. Biochem. J. 428, 463–471. 10.1042/BJ20100101 20377530

[B64] Ornitz D. M. Itoh N. (2015). The fibroblast growth factor signaling pathway. Wiley Interdiscip. Rev. Dev. Biol. 4, 215–266. 10.1002/wdev.176 25772309 PMC4393358

[B65] Pickrell J. K. Pritchard J. K. (2012). Inference of population splits and mixtures from genome-wide allele frequency data. PLoS Genet. 8, e1002967. 10.1371/journal.pgen.1002967 23166502 PMC3499260

[B66] Pidoux G. Taskén K. (2010). Specificity and spatial dynamics of protein kinase a signaling organized by A-kinase-anchoring proteins. J. Mol. Endocrinol. 44, 271–284. 10.1677/JME-10-0010 20150326

[B67] Purcell S. Neale B. Todd-Brown K. Thomas L. Ferreira M. A. R. Bender D. (2007). PLINK: a tool set for whole-genome association and population-based linkage analyses. Am. J. Hum. Genet. 81, 559–575. 10.1086/519795 17701901 PMC1950838

[B68] Purfield D. C. Berry D. P. McParland S. Bradley D. G. (2012). Runs of homozygosity and population history in cattle. BMC Genet. 13, 70. 10.1186/1471-2156-13-70 22888858 PMC3502433

[B69] R Core Team (2024). R: A language and environment for statistical computing. Vienna, Austria: R Foundation for Statistical Computing. Available online at: http://www.r-project.org

[B70] Rinchen D. Nazia J. (2023). Significance of wool trade in the political and economical development of Ladakh. Int. J. Creat. Res. Thoughts, 11 (5), M425–433. Available online at: https://ijcrt.org/papers/IJCRT23A5467.pdf (Accessed April 14, 2025).

[B71] Rochus C. M. Tortereau F. Plisson-Petit F. Restoux G. Moreno-Romieux C. Tosser-Klopp G. (2018). Revealing the selection history of adaptive loci using genome-wide scans for selection: an example from domestic sheep. BMC Genomics 19, 71. 10.1186/s12864-018-4447-x 29357834 PMC5778797

[B72] Saravanan K. A. Panigrahi M. Kumar H. Bhushan B. Dutt T. Mishra B. P. (2021). Genome-wide analysis of genetic diversity and selection signatures in three Indian sheep breeds. Livest. Sci. 243, 104367. 10.1016/j.livsci.2020.104367

[B73] Seifu W. D. Bekele-Alemu A. Zeng C. (2024). Genomic and physiological mechanisms of high-altitude adaptation in Ethiopian highlanders: a comparative perspective. Front. Genet. 15, 1510932. 10.3389/fgene.2024.1510932 39840284 PMC11747213

[B74] Sempéré G. Moazami-Goudarzi K. Eggen A. Laloë D. Gautier M. Flori L. (2015). WIDDE: a web-interfaced next generation database for genetic diversity exploration, with a first application in cattle. BMC Genomics 16, 940. 10.1186/s12864-015-2181-1 26573482 PMC4647285

[B75] Shi L. Wang L. Liu J. Deng T. Yan H. Zhang L. (2020). Estimation of inbreeding and identification of regions under heavy selection based on runs of homozygosity in a large white pig population. J. Anim. Sci. Biotechnol. 11, 46. 10.1186/s40104-020-00447-0 32355558 PMC7187514

[B76] Singh A. (2024). Livestock production statistics of India - 2024. Vet. Extensions. 10.13140/RG.2.2.32034.86721

[B77] Sridhar X. (2017). Study on temporal changes of Deccani sheep rearing in mahbubnagar district of Telangana state. India: P.V. Narsimha Rao Telangana Veterinary University. Available online at: http://krishikosh.egranth.ac.in/handle/1/5810021223.

[B78] Sved J. A. Feldman M. W. (1973). Correlation and probability methods for one and two loci. Theor. Popul. Biol. 4, 129–132. 10.1016/0040-5809(73)90008-7 4726005

[B79] Takeuchi K. Xu Y. Ogawa S. Ikejiri M. Nakatani K. Gotoh S. (2021). A pediatric case of productive cough caused by novel variants in DNAH9. Hum. Genome Var. 8, 3. 10.1038/s41439-020-00134-6 33452233 PMC7810879

[B80] Verity R. Collins C. Card D. C. Schaal S. M. Wang L. Lotterhos K. E. (2017). Minotaur: a platform for the analysis and visualization of multivariate results from genome scans with R Shiny. Mol. Ecol. Resour. 17, 33–43. 10.1111/1755-0998.12579 27473028

[B81] Vestweber D. (2015). How leukocytes cross the vascular endothelium. Nat. Rev. Immunol. 15, 692–704. 10.1038/nri3908 26471775

[B82] Voight B. F. Kudaravalli S. Wen X. Pritchard J. K. (2006). A map of recent positive selection in the human genome. PLoS Biol. 4, e72–e458. 10.1371/journal.pbio.0040072 16494531 PMC1382018

[B83] Webster J. M. Darling A. L. Uversky V. N. Blair L. J. (2019). Small heat shock proteins, big impact on protein aggregation in neurodegenerative disease. Front. Pharmacol. 10, 1047. 10.3389/fphar.2019.01047 31619995 PMC6759932

[B84] Wei C. Wang H. Liu G. Zhao F. Kijas J. W. Ma Y. (2016). Genome-wide analysis reveals adaptation to high altitudes in Tibetan sheep. Sci. Rep. 6, 26770. 10.1038/srep26770 27230812 PMC4882523

[B85] Wei Q. Zhong L. Zhang S. Mu H. Xiang J. Yue L. (2017). Bovine lineage specification revealed by single-cell gene expression analysis from zygote to blastocyst. Biol. Reprod. 97, 5–17. 10.1093/biolre/iox071 28859285

[B86] Weir B. S. Cockerham C. C. (1984). Estimating F-statistics for the analysis of population-structure. Evolution (N. Y) 38, 1358–1370. 10.1111/j.1558-5646.1984.tb05657.x 28563791

[B87] Yang J. Lee S. H. Goddard M. E. Visscher P. M. (2011). GCTA: a tool for genome-wide complex trait analysis. Am. J. Hum. Genet. 88, 76–82. 10.1016/j.ajhg.2010.11.011 21167468 PMC3014363

[B88] Yates A. D. Achuthan P. Akanni W. Allen J. Allen J. Alvarez-Jarreta J. (2020). Ensembl 2020. Nucleic Acids Res. 48, D682–D688. 10.1093/nar/gkz966 31691826 PMC7145704

[B89] Yin T. Green K. J. (2004). Regulation of desmosome assembly and adhesion. Semin. Cell Dev. Biol. 15, 665–677. 10.1016/j.semcdb.2004.09.005 15561586

[B90] Yuan A. Rao M. V. Nixon R. A. (2017). Neurofilaments and neurofilament proteins in health and disease. Cold Spring Harb. Perspect. Biol. 9, a018309. 10.1101/cshperspect.a018309 28373358 PMC5378049

[B91] Zocchi L. Wu S. C. Benavente C. A. (2020). Heavenly HELLS? A potential new therapeutic target for retinoblastoma. Oncoscience 7, 23–25. 10.18632/oncoscience.502 32426419 PMC7217136

