# Supplementary material for: Comparative genomic insights into adaptation, selection signatures, and population dynamics in indigenous Indian sheep and foreign breeds
Source: Front Genet. 2025 Aug 21;16:1621960. doi: 10.3389/fgene.2025.1621960 (PMC12408274; doi:10.3389/fgene.2025.1621960)
Supplement: Supplementary file 2 [file DataSheet3.pdf]

Supplementary Material

1 Supplementary Figures

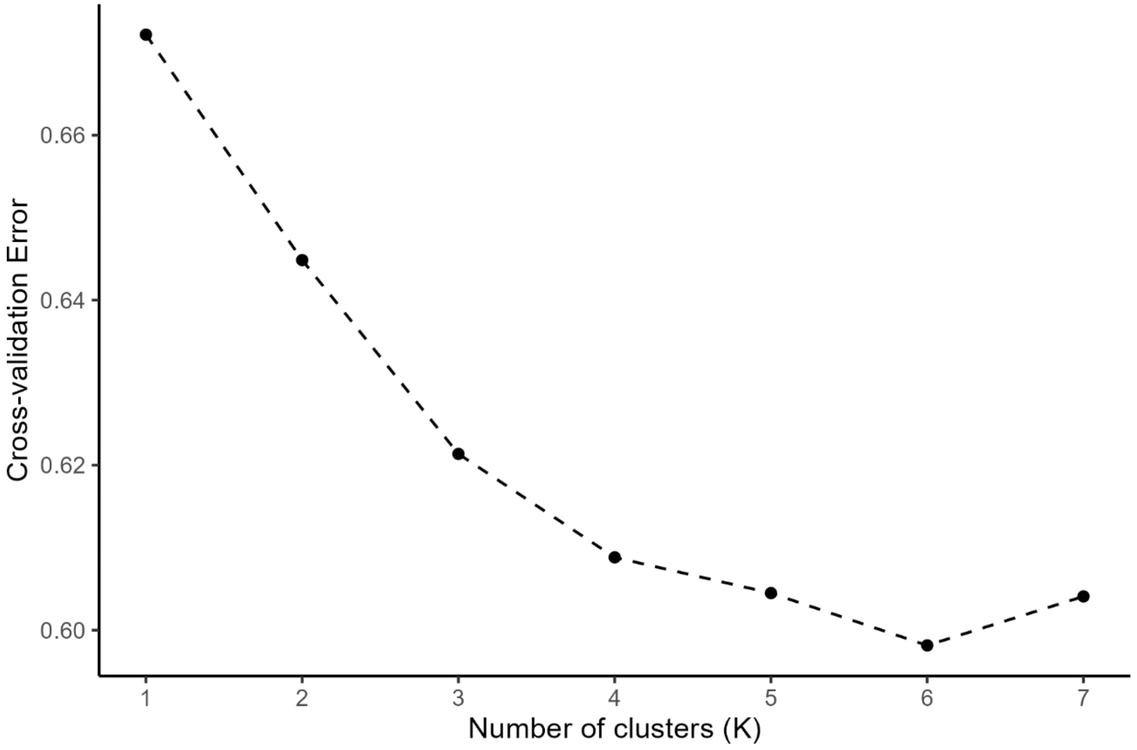

Supplementary Figure 1. Cross-Validation Error for ADMIXTURE Across K Values in Nine Sheep Breeds. Line plot illustrating the cross-validation error (y-axis) at each tested genetic cluster size ($K = 1$ to 7) for the nine sheep breeds under investigation. The optimal K is selected by identifying the minimum cross-validation error, which in this dataset occurs at $K = 6$.

2 Supplementary Tables

Supplementary Table S1. Summary of DCMS Outlier Windows Across Nine Sheep Breeds

BREED	CHR	START	END	DCMS_scores	DCMS_pvalues	DCMS_qvalues
BGA	1	68500001	6.90E+07	11.26820068	9.86E-06	0.009817116
BGA	1	163000001	163500000	9.426555307	0.000177773	0.045094495
BGA	1	164500001	1.65E+08	11.071398	1.37E-05	0.010577209
BGA	2	73000001	73500000	11.22435794	1.06E-05	0.009817116
BGA	3	182500001	1.83E+08	9.228830239	0.00023594	0.049558078
BGA	5	36000001	36500000	9.439966184	0.000174359	0.045094495
BGA	5	40000001	40500000	10.85650515	1.96E-05	0.012936449
BGA	5	41500001	4.20E+07	10.6584521	2.70E-05	0.015620167
BGA	6	82000001	82500000	9.594201981	0.000139261	0.04430099
BGA	7	56500001	5.70E+07	9.285540599	0.000217659	0.047895274
BGA	7	98000001	98500000	9.787671494	0.00010457	0.037861892
BGA	9	59000001	59500000	9.361738991	0.000195172	0.045094495
BGA	10	4000001	4500000	9.528161865	0.00015339	0.04430099
BGA	10	8000001	8500000	12.37613207	1.38E-06	0.003198827
BGA	12	2000001	2500000	10.12941436	6.23E-05	0.031969117
BGA	12	21000001	21500000	10.02840283	7.27E-05	0.033592816
BGA	15	6500001	7.00E+06	9.375506613	0.000191348	0.045094495
BGA	15	76000001	76500000	16.28138436	3.52E-10	1.63E-06
BGA	15	79000001	79500000	9.842412615	9.63E-05	0.037861892
BGA	18	4000001	4500000	9.775332068	0.000106515	0.037861892
BGA	20	33500001	3.40E+07	11.60065187	5.57E-06	0.00857892
BGA	22	48500001	4.90E+07	9.53887547	0.00015101	0.04430099
BGE	1	74000001	74500000	10.69482227	6.61E-05	0.038851724
BGE	1	247500001	2.48E+08	10.71672121	6.40E-05	0.038851724
BGE	3	53000001	53500000	12.11504053	7.48E-06	0.007033012
BGE	6	29500001	3.00E+07	10.52888933	8.39E-05	0.043842074
BGE	9	33000001	33500000	11.13906273	3.43E-05	0.026900979
BGE	18	30000001	30500000	13.36717208	8.93E-07	0.001687439
BGE	18	65500001	6.60E+07	13.26121119	1.08E-06	0.001687439
BGE	23	25500001	2.60E+07	14.49447324	1.12E-07	0.000525191
BGE	24	34500001	3.50E+07	12.21802693	6.32E-06	0.007033012
CHA	15	51000001	51500000	12.8702078	2.20E-06	0.010482167
CHA	19	55500001	5.60E+07	11.98966989	9.44E-06	0.022507375
CHA	21	3500001	4.00E+06	11.61295929	1.71E-05	0.027193484
CME	6	34000001	34500000	10.89792164	6.59E-06	0.010282697
CME	6	37500001	3.80E+07	10.01581877	3.11E-05	0.029062512
CME	6	38000001	38500000	10.24445385	2.10E-05	0.024588686
CME	13	73500001	7.40E+07	11.54398914	1.96E-06	0.009193126

CME	24	10000001	10500000	10.90602631	6.50E-06	0.010282697
EMZ	1	54000001	54500000	12.15619391	1.80E-06	0.001712011
EMZ	1	136000001	136500000	10.14079375	5.53E-05	0.021943113
EMZ	3	124500001	1.25E+08	14.25847502	2.75E-08	0.000131019
EMZ	5	12500001	1.30E+07	13.01477806	3.51E-07	0.000557931
EMZ	5	51500001	5.20E+07	9.729199956	0.000103961	0.03537651
EMZ	5	56000001	56500000	9.561619317	0.000133508	0.039751938
EMZ	13	49000001	49500000	11.32627644	7.89E-06	0.004698802
EMZ	13	49500001	5.00E+07	13.37636694	1.71E-07	0.000408081
EMZ	15	76500001	7.70E+07	11.61685316	4.75E-06	0.003233923
EMZ	16	1500001	2.00E+06	10.0900235	5.99E-05	0.021943113
EMZ	19	32000001	32500000	10.62681416	2.55E-05	0.011039896
EMZ	20	33500001	3.40E+07	11.99970952	2.39E-06	0.001899685
EMZ	22	13000001	13500000	9.573780327	0.000131123	0.039751938
EMZ	22	17000001	17500000	9.369312689	0.000177055	0.049616979
EMZ	23	41500001	4.20E+07	11.09779196	1.17E-05	0.005554955
EMZ	26	27500001	2.80E+07	11.10592691	1.15E-05	0.005554955
EMZ	26	33000001	33500000	12.61461087	7.61E-07	0.000906907
GAR	1	3500001	4.00E+06	8.626303027	0.000169371	0.047783869
GAR	1	135000001	135500000	9.293869321	5.65E-05	0.023188138
GAR	6	109000001	109500000	9.208452519	6.53E-05	0.023914083
GAR	10	35500001	3.60E+07	9.331335594	5.30E-05	0.023188138
GAR	10	36000001	36500000	10.51672204	6.26E-06	0.009416624
GAR	10	36500001	3.70E+07	9.474141343	4.15E-05	0.02292188
GAR	10	59000001	59500000	9.655063207	3.03E-05	0.019516802
GAR	10	59500001	6.00E+07	8.751212397	0.000138688	0.041735766
GAR	13	77500001	7.80E+07	9.763391975	2.50E-05	0.018802634
GAR	15	75000001	75500000	9.844746873	2.16E-05	0.018802634
GAR	16	60500001	6.10E+07	11.67093939	6.25E-07	0.001411047
GAR	17	53000001	53500000	9.418037199	4.57E-05	0.02292188
GAR	18	4500001	5.00E+06	9.176736521	6.89E-05	0.023914083
GAR	18	5000001	5500000	8.904008764	0.000108232	0.034897048
GAR	22	15500001	1.60E+07	12.80796983	5.20E-08	0.000234921
GAR	24	32500001	3.30E+07	10.2249197	1.08E-05	0.012209925
IDC	1	131000001	131500000	13.08851184	1.73E-07	0.000274809
IDC	1	167500001	1.68E+08	10.27606827	3.14E-05	0.01579406
IDC	2	216000001	216500000	11.17858523	6.70E-06	0.00639051
IDC	3	53000001	53500000	9.974853742	5.11E-05	0.020326608
IDC	3	105500001	1.06E+08	16.25265887	1.24E-10	5.93E-07
IDC	3	106000001	106500000	13.37320937	9.57E-08	0.000228306
IDC	3	107500001	1.08E+08	11.02300945	8.82E-06	0.007007621
IDC	4	35000001	35500000	9.679071285	8.16E-05	0.027805149
IDC	5	7500001	8.00E+06	9.472515714	0.000112294	0.033470521
IDC	7	55500001	5.60E+07	9.542716034	0.000100826	0.03205596
IDC	9	89500001	9.00E+07	10.20990861	3.50E-05	0.01579406
IDC	12	36500001	3.70E+07	10.44272436	2.38E-05	0.01579406
IDC	13	63500001	6.40E+07	9.707824231	7.80E-05	0.027805149

IDC	18	66500001	6.70E+07	10.35073186	2.77E-05	0.01579406
IDC	18	67000001	67500000	10.18472331	3.64E-05	0.01579406
IDC	22	15500001	1.60E+07	12.8523998	2.80E-07	0.00033342
SUF	1	221500001	2.22E+08	10.05826548	1.10E-05	0.008246734
SUF	2	40500001	4.10E+07	10.47706201	4.93E-06	0.006265267
SUF	3	53000001	53500000	9.93491754	1.38E-05	0.008246734
SUF	3	153500001	1.54E+08	9.920416428	1.42E-05	0.008246734
SUF	5	8500001	9.00E+06	11.13044671	1.33E-06	0.003080067
SUF	8	32000001	32500000	9.114132516	6.01E-05	0.025581355
SUF	10	32000001	32500000	10.26027152	7.49E-06	0.006952321
SUF	10	32500001	3.30E+07	9.72803523	2.03E-05	0.010444037
SUF	11	26500001	2.70E+07	8.872656503	9.06E-05	0.031714986
SUF	14	13500001	1.40E+07	8.798779674	0.000102527	0.031714986
SUF	18	25500001	2.60E+07	8.829391963	9.74E-05	0.031714986
SUF	18	26500001	2.70E+07	10.42991386	5.40E-06	0.006265267
SUF	18	29500001	3.00E+07	9.108983991	6.06E-05	0.025581355
SUF	20	33500001	3.40E+07	8.876228644	9.01E-05	0.031714986
SUF	23	26000001	26500000	11.63061838	4.63E-07	0.002148545
TIB	1	227000001	227500000	10.61657061	4.66E-05	0.020157115
TIB	2	40500001	4.10E+07	10.27897206	7.73E-05	0.026283817
TIB	2	112500001	1.13E+08	10.71754721	3.99E-05	0.019001247
TIB	2	115000001	115500000	13.59292097	2.83E-07	0.000672808
TIB	2	122500001	1.23E+08	11.67771316	8.61E-06	0.005856727
TIB	3	61000001	61500000	10.19625097	8.73E-05	0.027711481
TIB	3	84000001	84500000	10.39488231	6.50E-05	0.023826379
TIB	5	15500001	1.60E+07	11.08759722	2.24E-05	0.011857147
TIB	6	35500001	3.60E+07	11.92523469	5.69E-06	0.005416037
TIB	6	79000001	79500000	11.68527005	8.50E-06	0.005856727
TIB	9	48000001	48500000	10.54822031	5.17E-05	0.020497188
TIB	12	22500001	2.30E+07	13.16970991	6.26E-07	0.000994431
TIB	13	49500001	5.00E+07	12.27489404	3.12E-06	0.003717778
TIB	17	66000001	66500000	11.1069677	2.17E-05	0.011857147
TIB	20	33500001	3.40E+07	13.87589176	1.64E-07	0.000672808

This table lists the 118 significant DCMS windows ($q < 0.05$) identified in each breed, along with basic information such as breed name, chromosome, start and end coordinates, and the number of single nucleotide polymorphisms (SNPs) within each window. The analysis was performed using a 500 kb sliding window that integrated iHS, H12, ZHp, π, and Tajima’s D.

Supplementary Table S2. Annotated Genes Within Significant DCMS Windows

Breed	BTA	Start_bp	End_bp	Gene_symbol
BGA	1	68531997	68689225	HFM1
BGA	1	68792454	68818778	CDC7
BGA	1	68958611	69158590	TGFBR3
BGA	1	162972268	163007727	ARL6
BGA	1	163027934	163151757	CRYBG3
BGA	1	163152919	163185304	RIOX2
BGA	1	163197408	163244116	GABRR3
BGA	1	164940736	165008330	COL8A1
BGA	2	73009137	73030719	CDC37L1
BGA	2	73030072	73051926	AK3
BGA	2	73103727	73182368	RCL1
BGA	2	73156768	73156858	MIR101-2
BGA	2	73329555	73444824	JAK2
BGA	2	73461529	73474069	INSL6
BGA	3	182513473	182671107	PKP2
BGA	3	182650545	182718789	DNM1L
BGA	3	182745224	182984307	FGD4
BGA	5	35995315	36059775	UNC5A
BGA	5	36059754	36079369	HK3
BGA	5	36084066	36245885	UIMC1
BGA	5	36244865	36270306	ZNF346
BGA	5	36248163	36248266	U6
BGA	5	36287573	36298331	FGFR4
BGA	5	36329009	36471995	NSD1
BGA	5	36474873	36479942	RAB24
BGA	5	40251858	40262632	LYPD8
BGA	5	40338527	40352864	SH3BP5L
BGA	5	40352917	40373995	ZNF672
BGA	5	40370942	40379247	ZNF692
BGA	5	40473769	40482622	PGBD2
BGA	5	41499604	41518775	DAZAP1
BGA	5	41527077	41550798	APC2
BGA	5	41562424	41568238	PCSK4
BGA	5	41569562	41575203	REEP6
BGA	5	41582134	41589333	ADAMTSL5
BGA	5	41618007	41626593	MEX3D
BGA	5	41631475	41640113	MBD3
BGA	5	41653508	41685038	TCF3
BGA	5	41767893	41786685	ONECUT3
BGA	5	41791343	41812745	ATP8B3
BGA	5	41817273	41836172	REXO1
BGA	5	41840477	41850479	KLF16
BGA	5	41858472	41866771	ABHD17A
BGA	5	41887581	41904757	SCAMP4

BGA	5	41911162	41934787	CSNK1G2
BGA	5	41946948	41970121	BTBD2
BGA	7	56487940	56521257	MAPK6
BGA	7	56560517	56596546	LEO1
BGA	7	56605398	56698626	TMOD3
BGA	7	56713488	56771990	TMOD2
BGA	7	56788566	56802900	LYSMD2
BGA	7	56805034	56849519	SCG3
BGA	7	56894657	57067805	DMXL2
BGA	7	97976185	98135587	KCNK10
BGA	7	98177086	98217852	SPATA7
BGA	7	98232302	98304406	PTPN21
BGA	7	98319668	98362850	ZC3H14
BGA	7	98360007	98515564	EML5
BGA	9	58697417	59145257	SAMD12
BGA	9	59234759	59551841	EXT1
BGA	10	8306291	8306395	U6
BGA	12	1996886	2004981	PPP1R15B
BGA	12	2016904	2084994	PIK3C2B
BGA	12	2098776	2136195	MDM4
BGA	12	2201494	2268225	LRRN2
BGA	12	2450716	2647682	NFASC
BGA	12	20973423	21026308	RRP15
BGA	12	21040870	21135808	TGFB2
BGA	15	6442785	6582782	CEP126
BGA	15	6582349	6606149	ANGPTL5
BGA	15	75991276	76001752	ARFGAP2
BGA	15	76002420	76011655	PACSIN3
BGA	15	76026399	76045000	DDB2
BGA	15	76041381	76052290	ACP2
BGA	15	76052363	76065221	NR1H3
BGA	15	76066779	76106977	MADD
BGA	15	76107930	76125799	MYBPC3
BGA	15	76127141	76144083	SPI1
BGA	15	76167200	76175530	SLC39A13
BGA	15	76177501	76183466	PSMC3
BGA	15	76189000	76203367	RAPSN
BGA	15	76214842	76281488	CELF1
BGA	15	76298268	76304623	PTPMT1
BGA	15	76308945	76314652	NDUFS3
BGA	15	76314860	76316967	FAM180B
BGA	15	76316373	76322123	C1QTNF4
BGA	15	76335803	76356162	MTCH2
BGA	15	76372390	76404509	AGBL2
BGA	15	76406310	76437328	FNBP4
BGA	15	76428144	76494282	NUP160

BGA	15	79207844	79220370	LRRC55
BGA	15	79273486	79277459	APLNR
BGA	15	79332415	79357173	TNKS1BP1
BGA	15	79358357	79368148	SSRP1
BGA	15	79369867	79405301	P2RX3
BGA	15	79486906	79514196	SLC43A3
BGA	18	3739752	4565544	GABRG3
BGA	18	4181460	4181565	U6
BGA	20	33661664	33661768	U6
BGA	20	33685032	33697847	PRP2
BGA	20	33909222	33936091	PRP1
BGA	22	48661867	48779346	EBF3
BGA	22	48900380	48932416	GLRX3
BGE	1	74239082	75182110	DPYD
BGE	1	247471891	247622050	TFDP2
BGE	1	247642126	247676985	ATP1B3
BGE	1	247736925	247777782	GRK7
BGE	1	247806699	247806774	U6
BGE	1	247938792	248063116	RASA2
BGE	6	29597358	30017600	UNC5C
BGE	9	33296034	33701811	SNTG1
BGE	18	30022256	30346342	PEAK1
BGE	18	30346851	30447386	HMG20A
BGE	18	65727245	65734008	EIF5
BGE	18	65730682	65730808	SNORA28
BGE	18	65762081	65873058	MARK3
BGE	18	65892865	65902729	CKB
BGE	18	65901071	65907371	TRMT61A
BGE	18	65923406	65931964	BAG5
BGE	18	65930647	66040166	KLC1
BGE	23	25477225	25592176	RNF125
BGE	23	25592462	25677724	TRAPPC8
BGE	23	25799127	25869931	B4GALT6
BGE	23	25886082	25894684	TTR
BGE	23	25942407	25993761	DSG2
BGE	24	34512877	34528093	CCL24
BGE	24	34566531	34660573	POR
BGE	24	34660731	34666227	TMEM120A
BGE	24	34667238	34698895	STYXL1
BGE	24	34698135	34716699	MDH2
BGE	24	34727774	34781565	SRRM3
BGE	24	34788908	34790698	HSPB1
BGE	24	34810983	34836196	YWHAG
BGE	24	34857056	34868682	SSC4D
BGE	24	34903674	34937525	DTX2
BGE	24	34940485	34945204	UPK3B

CHA	15	50860388	51005864	ARAP1
CHA	15	51028830	51075998	STARD10
CHA	15	51076770	51095014	ATG16L2
CHA	15	51099187	51353916	FCHSD2
CHA	15	51404925	51511918	P2RY2
CHA	15	51481919	51516379	P2RY6
CHA	19	55260465	55530631	ATG7
CHA	19	55529201	55685269	VGLL4
CHA	19	55748216	55795704	TAMM41
CHA	19	55837608	55840639	TRH
CHA	19	55950931	55951034	U6
CHA	19	55972579	56051781	TMCC1
CME	6	33699466	35188219	CCSER1
CME	6	37519813	37531618	MEPE
CME	6	37552005	37565582	IBSP
CME	6	37810367	37834272	LAP3
CME	6	37856518	37976184	FAM184B
CME	6	37986716	38008444	DCAF16
CME	6	38007981	38052945	NCAPG
CME	6	38052622	38222929	LCORL
CME	13	73514433	73516914	SLPI
CME	13	73535608	73550713	MATN4
CME	13	73549055	73559879	RBPJL
CME	13	73568859	73590234	SDC4
CME	13	73616822	73624101	TP53TG5
CME	13	73647885	74304140	PIGT
CME	24	9982586	10012708	TVP23A
CME	24	10069022	10123958	CIITA
CME	24	10140503	10140790	DEXI
CME	24	10143620	10378415	CLEC16A
CME	24	10433864	10436027	SOCS1
CME	24	10453958	10455380	PRM3
CME	24	10458553	10459154	PRM1
EMZ	1	53742747	54015271	AK5
EMZ	1	54019798	54088317	ZZZ3
EMZ	1	54160619	54224621	USP33
EMZ	1	54243558	54320287	MIGA1
EMZ	1	54248797	54248904	U6
EMZ	1	54316939	54392748	NEXN
EMZ	1	54424058	54458997	DNAJB4
EMZ	1	54488786	54799266	GIPC2
EMZ	1	136054291	136656859	NCAM2
EMZ	3	124761395	124868190	KITLG
EMZ	5	12500710	12517684	ANGPTL6
EMZ	5	12504787	12510137	SHFL

EMZ	5	12519836	12519919	SNORD105
EMZ	5	12521245	12521324	SNORD105B
EMZ	5	12525801	12530457	EIF3G
EMZ	5	12536333	12585950	DNMT1
EMZ	5	12603818	12616271	S1PR2
EMZ	5	12619048	12619151	U6
EMZ	5	12626712	12635927	MRPL4
EMZ	5	12627845	12760150	TYK2
EMZ	5	12670619	12681109	ICAM1
EMZ	5	12681316	12683081	ICAM4
EMZ	5	12683659	12690947	ICAM5
EMZ	5	12781287	12792488	CDC37
EMZ	5	12793814	12841908	PDE4A
EMZ	5	12860990	12868555	KEAP1
EMZ	5	12870405	12881545	S1PR5
EMZ	5	12905446	12913957	ATG4D
EMZ	5	12913611	12923035	KRI1
EMZ	5	12923483	12925900	CDKN2D
EMZ	5	12927352	12938096	AP1M2
EMZ	5	12986576	13016358	ILF3
EMZ	5	51461116	51938080	ARHGAP26
EMZ	5	55516124	56038411	PPP2R2B
EMZ	5	56191958	56325773	STK32A
EMZ	5	56333373	56448480	DPYSL3
EMZ	15	76614185	76787236	PTPRJ
EMZ	15	76848267	76865157	OR4B1
EMZ	15	76992377	76993285	OR4C3
EMZ	16	1647962	1681358	SPDL1
EMZ	16	1693535	2188691	DOCK2
EMZ	16	1947797	2072678	INSYN2B
EMZ	19	32259247	32259350	U6
EMZ	19	32269372	32468660	FRMD4B
EMZ	20	33661664	33661768	U6
EMZ	20	33685032	33697847	PRP2
EMZ	20	33909222	33936091	PRP1
EMZ	22	13010836	13016213	PPP1R3C
EMZ	22	13144737	13249292	TNKS2
EMZ	22	13249174	13334730	BTAF1
EMZ	22	13372860	13525886	CPEB3
EMZ	22	16980694	17003028	TCTN3
EMZ	22	17029353	17129306	ENTPD1
EMZ	22	17140114	17232443	CC2D2B
EMZ	22	17243641	17261428	CCNJ
EMZ	22	17290399	17351658	ZNF518A
EMZ	22	17352255	17431162	BLNK
EMZ	22	17468517	17500493	DNTT

EMZ	23	41573978	41742730	MTCL1
EMZ	23	41928790	41949779	NDUFV2
EMZ	23	41951735	42050658	ANKRD12
EMZ	26	26633835	27770221	NRG1
EMZ	26	32927857	33051668	TACC1
EMZ	26	33108956	33175085	PLEKHA2
EMZ	26	33175428	33188708	HTRA4
EMZ	26	33187935	33196411	TM2D2
EMZ	26	33195611	33293408	ADAM9
EMZ	26	33297142	33495158	ADAM32
GAR	1	3592149	3631165	RAB17
GAR	1	3636688	3639554	PRLH
GAR	1	3645956	3685179	MLPH
GAR	1	3777567	3871102	COL6A3
GAR	6	109196576	109352162	RAB28
GAR	6	109408969	109412689	NKX3-2
GAR	6	109433497	109488047	BOD1L1
GAR	10	35503970	35504080	U5
GAR	10	35627342	35654346	FGF9
GAR	10	35711291	35763234	MICU2
GAR	10	35771362	35830743	ZDHHC20
GAR	10	35821680	35868894	SAP18
GAR	10	35906167	35930911	LATS2
GAR	10	35942141	36032666	XPO4
GAR	10	36036449	36056966	EEF1AKMT1
GAR	10	36056159	36087431	IL17D
GAR	10	36091192	36155341	IFT88
GAR	10	36167175	36227039	CRYL1
GAR	10	36289437	36299065	GJB6
GAR	10	36311927	36318646	GJB2
GAR	10	36348743	36349981	GJA3
GAR	10	36366508	36431754	ZMYM2
GAR	10	36473798	36498569	ZMYM5
GAR	10	36508476	36558287	PSPC1
GAR	10	36583106	36631226	MPHOSPH8
GAR	10	36635009	36709921	PARP4
GAR	10	36689639	36689747	U6
GAR	10	36713071	36755812	CENPJ
GAR	10	36759213	36874081	RNF17
GAR	10	36881637	36903654	ATP12A
GAR	10	59369191	59374385	SLITRK1
GAR	13	77464675	77573379	KCNB1
GAR	13	77585124	77649366	PTGIS
GAR	13	77764562	77838591	B4GALT5
GAR	13	77923181	77994841	SLC9A8
GAR	15	74891000	75080236	PHF21A

GAR	15	75216377	75254154	CREB3L1
GAR	15	75264085	75308592	DGKZ
GAR	15	75308859	75311865	MDK
GAR	15	75312326	75320085	CHRM4
GAR	15	75323865	75489635	AMBRA1
GAR	15	75497354	75506938	HARBI1
GAR	17	52982058	53003350	ZCCHC8
GAR	17	53034654	53150646	CLIP1
GAR	17	53155280	53187904	VPS33A
GAR	17	53191164	53209476	DIABLO
GAR	17	53208273	53214458	B3GNT4
GAR	17	53214865	53230223	LRRC43
GAR	17	53258241	53316745	MLXIP
GAR	17	53346207	53346387	U2
GAR	17	53377551	53450150	CFAP251
GAR	17	53450758	53467449	PSMD9
GAR	17	53491709	53503621	HPD
GAR	18	3739752	4565544	GABRG3
GAR	18	4870665	5011557	LRRK1
GAR	18	5014822	5059585	ALDH1A3
GAR	18	5367490	5432181	ASB7
GAR	18	5432563	5463533	LINS1
GAR	18	5485863	5649853	CERS3
GAR	22	15475994	15571660	TBC1D12
GAR	22	15577621	15613672	HELLS
IDC	1	130838877	131151750	APP
IDC	1	131097374	131097476	U6
IDC	1	131262355	131297636	GABPA
IDC	1	131301424	131310461	ATP5PF
IDC	1	131315873	131396073	JAM2
IDC	1	131445637	131471859	MRPL39
IDC	1	131469794	131469855	MIR155
IDC	1	167799790	167853202	ZPLD1
IDC	2	215270428	216388688	SPAG16
IDC	2	216389872	216397929	VWC2L
IDC	2	216432053	216432160	U6
IDC	3	105922128	106017137	ANAPC1
IDC	3	105995668	105995772	U6
IDC	3	106026856	106144454	MERTK
IDC	3	106038477	106038611	SNORA70
IDC	3	106162506	106212618	TMEM87B
IDC	3	106222211	106279403	FBLN7
IDC	3	106298892	106326975	ZC3H8
IDC	3	106341587	106414232	ZC3H6
IDC	3	107555919	107687256	LGR5
IDC	3	107711708	107761748	ZFC3H1

IDC	3	107761881	107778268	THAP2
IDC	3	107786855	107806581	TMEM19
IDC	3	107848064	107878324	RAB21
IDC	3	107917450	107989336	TBC1D15
IDC	4	34945297	35187671	GRM3
IDC	5	7511929	7518138	PGLYRP2
IDC	5	7521113	7532646	RASAL3
IDC	5	7534739	7560855	WIZ
IDC	5	7563858	7594664	AKAP8L
IDC	5	7594977	7612778	AKAP8
IDC	5	7719890	7725421	EPHX3
IDC	5	7738891	7777391	NOTCH3
IDC	5	7846906	7859708	ILVBL
IDC	5	7859718	7866461	SYDE1
IDC	5	7941341	7950062	CASP14
IDC	5	7988254	7996892	TEKTL1
IDC	7	55749258	55785821	ONECUT1
IDC	7	55858518	55944614	ATOSA
IDC	7	55985537	56178067	MYO5A
IDC	9	89466002	89521152	ATP6V0D2
IDC	9	89955689	89972999	CA2
IDC	9	89989217	90000497	CA3
IDC	12	36438034	36514257	F5
IDC	12	36517216	36563902	SELP
IDC	12	36593263	36646144	SELL
IDC	12	36654412	36664396	SELE
IDC	12	36725261	36734295	METTL18
IDC	12	36733693	36784718	FIRRM
IDC	12	36748132	36748238	U6
IDC	12	36781989	36827145	SCYL3
IDC	12	36789289	36789406	5S_rRNA
IDC	12	36846163	37002350	KIFAP3
IDC	13	63591538	63672121	ASIP
IDC	13	63655270	63655469	SNORA73
IDC	13	63683003	63698721	AHCY
IDC	13	63782107	63882093	ITCH
IDC	13	63886063	63907106	DYNLRB1
IDC	13	63921399	64014856	PIGU
IDC	18	66795018	66799106	C14orf180
IDC	18	66799632	66811403	TMEM179
IDC	18	66895159	66922941	INF2
IDC	18	66926236	66944932	ADSS1
IDC	18	66952103	66961841	SIVA1
IDC	18	66960100	66985480	AKT1
IDC	18	66985934	66990322	ZBTB42
IDC	18	67040397	67065285	CEP170B

IDC	18	67136553	67156820	CDCA4
IDC	18	67244916	67265851	JAG2
IDC	18	67270700	67277828	NUDT14
IDC	18	67292959	67349872	BRF1
IDC	18	67313851	67316510	BTBD6
IDC	18	67363814	67423588	PACS2
IDC	18	67424321	67440112	TEX22
IDC	18	67445052	67478617	MTA1
IDC	18	67494400	67495971	CRIP1
IDC	18	67498986	67506292	TEDC1
IDC	22	15475994	15571660	TBC1D12
IDC	22	15577621	15613672	HELLS
SUF	2	40232325	40508711	DOCK5
SUF	2	40553888	40553984	U6
SUF	2	40732317	40737962	NEFL
SUF	2	40763601	40768907	NEFM
SUF	3	152867847	153638959	GRIP1
SUF	3	153534681	153534845	U1
SUF	3	153645046	153681787	HELB
SUF	3	153725010	153825702	IRAK3
SUF	3	153803744	153820110	TMBIM4
SUF	3	153825799	153833645	LLPH
SUF	8	31762789	32003154	PREP
SUF	8	32133452	32154523	POPDC3
SUF	8	32174084	32226001	BVES
SUF	8	32243781	32370762	LIN28B
SUF	8	32380188	32547757	HACE1
SUF	10	31896107	32103106	FLT1
SUF	10	32111956	32231722	PAN3
SUF	10	32265439	32332988	FLT3
SUF	10	32363890	32370374	CDX2
SUF	10	32397584	32402746	PDX1
SUF	10	32489303	32490544	GSX1
SUF	10	32602775	32691385	LNX2
SUF	10	32757761	32767063	MTIF3
SUF	10	32764479	32776549	GTF3A
SUF	10	32885509	32888038	RASL11A
SUF	10	32898061	32898187	SNORA27
SUF	10	32898434	32898504	SNORD102
SUF	10	32939035	32993883	USP12
SUF	11	26500105	26502509	MED11
SUF	11	26502666	26550663	PELP1
SUF	11	26515812	26524299	ARRB2
SUF	11	26580582	26589340	ALOX15
SUF	11	26672919	26686500	ALOX12
SUF	11	26688274	26690214	RNASEK

SUF	11	26689446	26689552	U6
SUF	11	26690332	26693536	C17orf49
SUF	11	26693334	26693424	MIR195
SUF	11	26693661	26693742	MIR497
SUF	11	26699032	26705046	BCL6B
SUF	11	26707334	26710816	SLC16A13
SUF	11	26711463	26713938	SLC16A11
SUF	11	26717519	26721666	CLEC10A
SUF	11	26739669	26750920	ASGR2
SUF	11	26786579	26790121	ASGR1
SUF	11	26799836	26824423	DLG4
SUF	11	26822539	26830608	ACADVL
SUF	11	26830291	26838569	DVL2
SUF	11	26839069	26843505	PHF23
SUF	11	26844318	26847059	GABARAP
SUF	11	26846581	26860812	ELP5
SUF	11	26846738	26854504	CTDNEP1
SUF	11	26860773	26863167	CLDN7
SUF	11	26875375	26886834	SLC2A4
SUF	11	26881708	26888124	YBX2
SUF	11	26910321	26913587	GPS2
SUF	11	26913436	26925484	NEURL4
SUF	11	26928247	26974403	ACAP1
SUF	11	26963875	26968698	PLSCR3
SUF	11	26979488	26990331	NLGN2
SUF	11	26991669	26994153	SPEM1
SUF	11	26995314	26997840	SPEM2
SUF	14	13483772	13540727	PIEZO1
SUF	14	13553184	13558628	CDT1
SUF	14	13558172	13560786	APRT
SUF	14	13561449	13577385	GALNS
SUF	14	13577043	13582678	TRAPPC2L
SUF	14	13585359	13588364	PABPN1L
SUF	14	13594703	13672143	CBFA2T3
SUF	14	13748020	13791746	ACSF3
SUF	14	13803299	13822337	CDH15
SUF	14	13821523	13827734	SLC22A31
SUF	14	13877315	13904711	ANKRD11
SUF	18	25536045	25536151	U6
SUF	18	25915771	25981741	ENTREP2
SUF	18	26585782	26631036	TARS3
SUF	18	26632892	26645471	TM2D3
SUF	18	26783010	26954584	PCSK6
SUF	18	26966934	26978763	SNRPA1
SUF	18	29407244	29803577	SCAPER
SUF	18	29827259	29848476	RCN2

SUF	18	29893030	29937608	PSTPIP1
SUF	18	29951595	29981692	TSPAN3
SUF	20	33661664	33661768	U6
SUF	20	33685032	33697847	PRP2
SUF	20	33909222	33936091	PRP1
SUF	23	26002837	26043852	DSG3
SUF	23	26069242	26110100	DSG4
SUF	23	26123882	26165793	DSG1
SUF	23	26315275	26349888	DSC1
SUF	23	26368660	26443218	DSC2
SUF	23	26423374	26480365	DSC3
TIB	1	226780772	227034992	NMD3
TIB	1	227033921	227068355	B3GALNT1
TIB	1	227090037	227417600	PPM1L
TIB	2	40232325	40508711	DOCK5
TIB	2	40553888	40553984	U6
TIB	2	40732317	40737962	NEFL
TIB	2	40763601	40768907	NEFM
TIB	2	112478935	112565977	ANXA10
TIB	2	112618922	112740892	MFSD14B
TIB	2	122556252	122576207	ZSWIM2
TIB	2	122603799	122685241	FAM171B
TIB	2	122690340	122796476	ITGAV
TIB	2	122884739	122905357	ZC3H15
TIB	3	61156855	61210753	ST6GAL2
TIB	3	83647866	84106592	SLC8A1
TIB	3	84415879	84454319	THUMPD2
TIB	3	84472567	84530866	TMEM178A
TIB	5	15491406	15502432	TRIP10
TIB	5	15503325	15510999	GPR108
TIB	5	15765584	15770209	CD70
TIB	5	15825236	15828844	TNFSF9
TIB	5	15844241	15855958	TUBB4A
TIB	5	15857836	15867883	DENND1C
TIB	5	15867684	15870898	CRB3
TIB	5	15873258	15886987	SLC25A23
TIB	5	15889274	15897106	SLC25A41
TIB	5	15898270	15912887	KHSRP
TIB	5	15924437	15931276	GTF2F1
TIB	5	15934733	15935278	PSPN
TIB	5	15935308	15937174	ALKBH7
TIB	5	15941024	15946448	CLPP
TIB	5	15967438	15990600	ACER1
TIB	6	35534086	35727809	SNCA
TIB	6	79065569	79065675	U6
TIB	9	47739721	48107324	EYA1

TIB	9	48396114	48397820	MSC
TIB	12	22662326	22694765	SLC30A10
TIB	12	22706059	22770662	EPRS1
TIB	12	22789651	22814286	BPNT1
TIB	12	22817543	22868995	IARS2
TIB	12	22865928	22969198	RAB3GAP2
TIB	12	22914904	22915035	SNORA36B
TIB	17	66061289	66257661	SEZ6L
TIB	17	66295916	66304481	ASPHD2
TIB	17	66309904	66335615	HPS4
TIB	17	66335643	66357818	SRRD
TIB	17	66353143	66371931	TFIP11
TIB	17	66381043	66437040	TPST2
TIB	17	66444152	66454477	CRYBB1
TIB	17	66459962	66466488	CRYBA4
TIB	20	33661664	33661768	U6
TIB	20	33685032	33697847	PRP2
TIB	20	33909222	33936091	PRP1

This table presents all protein-coding genes that lie within the DCMS outlier regions for each of the nine breeds. Columns include the breed name, chromosome, genomic coordinates, and official gene symbols. Redundant entries found in multiple windows are indicated accordingly. These annotations provide an overview of candidate loci potentially associated with adaptation, production traits, or other biological functions

Supplementary Table S3. Functional Enrichment of Genes Identified in DCMS Outlier Windows

BREED	Category	Term	Count	X.	PValue	Genes	List.Total	Pop.Hits	Pop.Total	Fold.Enrichment	Bonferroni	Benjamini	FDR
CHA	GOTERM_MF_DIRECT	GO:0045030~G protein-coupled UTP receptor activity	2	20	0.001239	P2RY6, P2RY2	9	3	19369	1434.741	0.017202	0.017341	0.017341
CHA	KEGG_PATHWAY	oas04080:Neuroactive ligand-receptor interaction	3	30	0.01439	P2RY6, P2RY2, TRH	6	383	9691	12.65144	0.096487	0.115124	0.115124
CHA	GOTERM_BP_DIRECT	GO:0000045~autophagosome assembly	2	20	0.018772	ATG16L2, ATG7	9	44	18600	93.93939	0.454702	0.581941	0.581941
CHA	GOTERM_MF_DIRECT	GO:0008289~lipid binding	2	20	0.056	FCHSD2, STARD10	9	139	19369	30.96563	0.553717	0.391998	0.391998
CHA	KEGG_PATHWAY	oas04148:Efferocytosis	2	20	0.08326	P2RY6, P2RY2	6	167	9691	19.34331	0.455845	0.245046	0.245046
CHA	KEGG_PATHWAY	oas04140:Autophagy - animal	2	20	0.091892	ATG16L2, ATG7	6	185	9691	17.46126	0.490714	0.245046	0.245046
IDC	GOTERM_BP_DIRECT	GO:0007157~heterophilic cell-cell adhesion via plasma membrane cell adhesion molecules	5	7.352941	1.13E-06	JAG2, SELP, NOTCH3, SELL, SELE	52	29	18600	61.67109	0.000225	0.00016	0.00016
IDC	GOTERM_BP_DIRECT	GO:0050901~leukocyte tethering or rolling	4	5.882353	1.61E-06	SELP, SELL, SELE, JAM2	52	9	18600	158.9744	0.000321	0.00016	0.00016
IDC	GOTERM_MF_DIRECT	GO:0070492~oligosaccharide binding	3	4.411765	0.000513	SELP, SELL, SELE	56	12	19369	86.46875	0.045628	0.04669	0.04669
IDC	GOTERM_BP_DIRECT	GO:0007219~Notch signaling pathway	4	5.882353	0.001055	JAG2, NOTCH3, APP, ONECUT1	52	73	18600	19.59958	0.189481	0.060559	0.060559
IDC	GOTERM_BP_DIRECT	GO:0034097~response to cytokine	3	4.411765	0.001223	SELP, SELL, SELE	52	19	18600	56.47773	0.216205	0.060559	0.060559
IDC	GOTERM_MF_DIRECT	GO:0033691~sialic acid binding	3	4.411765	0.001606	SELP, SELL, SELE	56	21	19369	49.41071	0.136069	0.073072	0.073072
IDC	KEGG_PATHWAY	oas04514:Cell adhesion molecules	4	5.882353	0.01487	SELP, SELL, SELE, JAM2	32	163	9691	7.431748	0.898962	1	1
IDC	KEGG_PATHWAY	oas04330:Notch signaling pathway	3	4.411765	0.017134	JAG2, NOTCH3, ITCH	32	63	9691	14.42113	0.928939	1	1
IDC	GOTERM_MF_DIRECT	GO:0003682~chromatin binding	4	5.882353	0.030098	HELLS, MTA1, ONECUT1, GABPA	56	238	19369	5.813025	0.938021	0.711583	0.711583
IDC	GOTERM_CC_DIRECT	GO:0032991~protein-containing complex	3	4.411765	0.031396	JAG2, NOTCH3, METTL18	54	108	20655	10.625	0.892787	1	1
IDC	KEGG_PATHWAY	oas01522:Endocrine resistance	3	4.411765	0.038202	JAG2, NOTCH3, AKT1	32	97	9691	9.366302	0.997419	1	1
IDC	GOTERM_MF_DIRECT	GO:0034237~protein kinase A regulatory subunit binding	2	2.941176	0.041772	AKAP8L, AKAP8	56	15	19369	46.11667	0.97941	0.711583	0.711583
IDC	GOTERM_BP_DIRECT	GO:0016477~cell migration	3	4.411765	0.043512	SYDE1, ONECUT1, MERTK	52	121	18600	8.868404	0.999857	1	1
IDC	GOTERM_MF_DIRECT	GO:0005112~Notch binding	2	2.941176	0.044495	JAG2, NOTCH3	56	16	19369	43.23438	0.984108	0.711583	0.711583
IDC	GOTERM_CC_DIRECT	GO:0005615~extracellular space	8	11.76471	0.047455	SELP, APP, SELL, ASIP, VWC2L, ZPLD1, MERTK, SELE	54	1301	20655	2.352037	0.966735	1	1
IDC	GOTERM_CC_DIRECT	GO:0045177~apical part of cell	2	2.941176	0.047664	APP, CA2	54	19	20655	40.26316	0.967242	1	1
IDC	GOTERM_MF_DIRECT	GO:0004089~carbonate dehydratase activity	2	2.941176	0.052619	CA3, CA2	56	19	19369	36.40789	0.992693	0.711583	0.711583

IDC	GOTERM_MF_DIRECT	GO:0046872~metal ion binding	10	14.70588	0.054737	SELP, APP, MTA1, SIVA1, SELL, AKAP8L, AKAP8, ZC3H8, ZC3H6, NUDT14	56	1738	19369	1.990075	0.994039	0.711583	0.711583
IDC	KEGG_PATHWAY	oas00910:Nitrogen metabolism	2	2.941176	0.056088	CA3, CA2	32	18	9691	33.64931	0.999854	1	1
IDC	GOTERM_BP_DIRECT	GO:0007275~multicellular organism development	3	4.411765	0.060777	JAG2, NOTCH3, MERTK	52	146	18600	7.349842	0.999996	1	1
IDC	GOTERM_CC_DIRECT	GO:0016363~nuclear matrix	2	2.941176	0.062247	AKAP8L, AKAP8	54	25	20655	30.6	0.988878	1	1
IDC	KEGG_PATHWAY	oas04668:TNF signaling pathway	3	4.411765	0.065368	ITCH, AKT1, SELE	32	131	9691	6.935353	0.999968	1	1
IDC	GOTERM_BP_DIRECT	GO:0045197~establishment or maintenance of epithelial cell apical/basal polarity	2	2.941176	0.066381	JAG2, NOTCH3	52	25	18600	28.61538	0.999999	1	1
IDC	GOTERM_BP_DIRECT	GO:0009755~hormone-mediated signaling pathway	2	2.941176	0.071501	ASIP, LGR5	52	27	18600	26.49573	1	1	1
IDC	GOTERM_MF_DIRECT	GO:0030246~carbohydrate binding	3	4.411765	0.07662	SELP, SELL, SELE	56	161	19369	6.444876	0.999293	0.871548	0.871548
IDC	GOTERM_BP_DIRECT	GO:0016339~calcium-dependent cell-cell adhesion via plasma membrane cell adhesion molecules	2	2.941176	0.081658	SELP, SELL	52	31	18600	23.07692	1	1	1
IDC	KEGG_PATHWAY	oas05224:Breast cancer	3	4.411765	0.084571	JAG2, NOTCH3, AKT1	32	152	9691	5.977179	0.999999	1	1
IDC	GOTERM_CC_DIRECT	GO:0005634~nucleus	17	25	0.085064	NOTCH3, HELLS, APP, BRF1, SIVA1, AKAP8L, ONECUT1, AKAP8, ZFC3H1, ZC3H8, ZBTB42, WIZ, ZC3H6, THAP2, ATOSA, AKT1, GABPA	54	4419	20655	1.471487	0.998017	1	1
IDC	KEGG_PATHWAY	oas04966:Collecting duct acid secretion	2	2.941176	0.085919	CA2, ATP6V0D2	32	28	9691	21.6317	0.999999	1	1
IDC	GOTERM_MF_DIRECT	GO:0005096~GTPase activator activity	3	4.411765	0.086649	SYDE1, RASAL3, TBC1D15	56	173	19369	5.997832	0.999738	0.87612	0.87612
IDC	GOTERM_BP_DIRECT	GO:0006006~glucose metabolic process	2	2.941176	0.089204	ONECUT1, AKT1	52	34	18600	21.04072	1	1	1
GAR	GOTERM_BP_DIRECT	GO:1990349~gap junction-mediated intercellular transport	3	5.357143	7.78E-05	GJB2, GJA3, GJB6	44	6	18600	211.3636	0.013378	0.01339	0.01339
GAR	GOTERM_CC_DIRECT	GO:0005922~connexin complex	3	5.357143	0.001244	GJB2, GJA3, GJB6	48	23	20655	56.12772	0.075406	0.078352	0.078352
GAR	GOTERM_BP_DIRECT	GO:0007154~cell communication	3	5.357143	0.003664	GJB2, GJA3, GJB6	44	39	18600	32.51748	0.470088	0.315108	0.315108
GAR	GOTERM_BP_DIRECT	GO:0016264~gap junction assembly	2	3.571429	0.009216	GJB2, GJB6	44	4	18600	211.3636	0.79846	0.528386	0.528386
GAR	GOTERM_MF_DIRECT	GO:1903763~gap junction channel activity involved in cell communication by electrical coupling	2	3.571429	0.010287	GJB2, GJB6	51	4	19369	189.8922	0.571676	0.797646	0.797646
GAR	GOTERM_BP_DIRECT	GO:0048706~embryonic skeletal system development	2	3.571429	0.018349	FGF9, NKX3-2	44	8	18600	105.6818	0.959396	0.789011	0.789011

GAR	GOTERM_MF_DIRECT	GO:0005243~gap junction channel activity	2	3.571429	0.022999	GJB2, GJA3	51	9	19369	84.39651	0.851618	0.797646	0.797646
GAR	GOTERM_MF_DIRECT	GO:0005515~protein binding	12	21.42857	0.029182	CFAP251, PSMD9, LATS2, KCNB1, SLITRK1, LRRK1, ASB7, COL6A3, AMBRA1, LRRC43, DGKZ, MPHOSPH8	51	2288	19369	1.991876	0.911835	0.797646	0.797646
GAR	GOTERM_CC_DIRECT	GO:0000139~Golgi membrane	4	7.142857	0.03369	SLC9A8, B3GNT4, HPD, B4GALT5	48	311	20655	5.534566	0.884563	0.935742	0.935742
GAR	GOTERM_CC_DIRECT	GO:0032590~dendrite membrane	2	3.571429	0.044559	KCNB1, GABRG3	48	20	20655	43.03125	0.943397	0.935742	0.935742
GAR	GOTERM_MF_DIRECT	GO:0008083~growth factor activity	3	5.357143	0.053322	FGF9, MDK, DGKZ	51	144	19369	7.912173	0.988816	1	1
GAR	GOTERM_CC_DIRECT	GO:0005634~nucleus	16	28.57143	0.061409	CERS3, MLXIP, HELLS, PARP4, HARBI1, DGKZ, PSMD9, CLIP1, LATS2, XPO4, PSPC1, CREB3L1, ASB7, SAP18, MPHOSPH8, NKX3-2	48	4419	20655	1.558045	0.98155	0.967194	0.967194
GAR	GOTERM_BP_DIRECT	GO:0007267~cell-cell signaling	2	3.571429	0.064965	GJB2, FGF9	44	29	18600	29.15361	0.999991	1	1
GAR	GOTERM_BP_DIRECT	GO:0051781~positive regulation of cell division	2	3.571429	0.077882	MDK, DGKZ	44	35	18600	24.15584	0.999999	1	1
GAR	KEGG_PATHWAY	oas01100:Metabolic pathways	9	16.07143	0.087513	CERS3, ALDH1A3, PTGIS, B3GNT4, CRYL1, ATP12A, DGKZ, HPD, B4GALT5	28	1705	9691	1.826959	0.9998	1	1
GAR	GOTERM_BP_DIRECT	GO:0015031~protein transport	3	5.357143	0.097438	XPO4, VPS33A, RAB17	44	228	18600	5.562201	1	1	1
BGA	GOTERM_BP_DIRECT	GO:0008284~positive regulation of cell population proliferation	5	5.102041	0.006708	PRP2, TGFB2, PRP1, FGFR4, JAK2	77	183	18600	6.599957	0.860824	1	1
BGA	KEGG_PATHWAY	oas04080:Neuroactive ligand-receptor interaction	6	6.122449	0.026355	GABRR3, PRP2, P2RX3, PRP1, APLNR, GABRG3	44	383	9691	3.450392	0.970565	1	1
BGA	KEGG_PATHWAY	oas04550:Signaling pathways regulating pluripotency of stem cells	4	4.081633	0.02665	APC2, TCF3, FGFR4, JAK2	44	146	9691	6.034247	0.971718	1	1
BGA	GOTERM_BP_DIRECT	GO:0051694~pointed-end actin filament capping	2	2.040816	0.03223	TMOD3, TMOD2	77	8	18600	60.38961	0.999932	1	1
BGA	GOTERM_BP_DIRECT	GO:0032570~response to progesterone	2	2.040816	0.03223	TGFB2, NR1H3	77	8	18600	60.38961	0.999932	1	1
BGA	GOTERM_CC_DIRECT	GO:0005865~striated muscle thin filament	2	2.040816	0.038125	TMOD3, TMOD2	90	9	20655	51	0.979496	1	1
BGA	GOTERM_BP_DIRECT	GO:0030239~myofibril assembly	2	2.040816	0.044051	TMOD3, TMOD2	77	11	18600	43.91972	0.999998	1	1
BGA	KEGG_PATHWAY	oas04917:Prolactin signaling pathway	3	3.061224	0.054457	PRP2, PRP1, JAK2	44	85	9691	7.773529	0.999383	1	1
BGA	GOTERM_MF_DIRECT	GO:0005523~tropomyosin binding	2	2.040816	0.060418	TMOD3, TMOD2	87	14	19369	31.8046	0.999873	1	1
BGA	GOTERM_BP_DIRECT	GO:1903489~positive regulation of lactation	2	2.040816	0.067265	PRP2, PRP1	77	17	18600	28.41864	1	1	1
BGA	GOTERM_MF_DIRECT	GO:0005148~prolactin receptor binding	2	2.040816	0.072888	PRP2, PRP1	87	17	19369	26.19202	0.999982	1	1
BGA	GOTERM_CC_DIRECT	GO:0043235~receptor complex	3	3.061224	0.07788	P2RX3, NR1H3, FGFR4	90	107	20655	6.434579	0.999699	1	1

BGA	KEGG_PATHWAY	oas05210:Colorectal cancer	3	3.061224	0.08143	APC2, TGFB2, DDB2	44	107	9691	6.175234	0.999986	1	1
BGA	GOTERM_BP_DIRECT	GO:0031667~response to nutrient levels	2	2.040816	0.082432	PRP2, PRP1	77	21	18600	23.00557	1	1	1
BGA	GOTERM_CC_DIRECT	GO:1902711~GABA-A receptor complex	2	2.040816	0.082776	GABRR3, GABRG3	90	20	20655	22.95	0.999823	1	1
BGA	GOTERM_MF_DIRECT	GO:0004890~GABA-A receptor activity	2	2.040816	0.085194	GABRR3, GABRG3	87	20	19369	22.26322	0.999997	1	1
BGA	KEGG_PATHWAY	oas04820:Cytoskeleton in muscle cells	4	4.081633	0.086478	MYBPC3, TMOD3, TMOD2, PKP2	44	236	9691	3.733051	0.999993	1	1
BGA	GOTERM_BP_DIRECT	GO:0016477~cell migration	3	3.061224	0.087696	APC2, TGFB2, PIK3C2B	77	121	18600	5.989052	1	1	1
BGA	GOTERM_BP_DIRECT	GO:0006936~muscle contraction	2	2.040816	0.089924	TMOD3, TMOD2	77	23	18600	21.00508	1	1	1
BGA	GOTERM_BP_DIRECT	GO:0030879~mammary gland development	2	2.040816	0.089924	PRP2, PRP1	77	23	18600	21.00508	1	1	1
BGA	GOTERM_CC_DIRECT	GO:0030016~myofibril	2	2.040816	0.090671	TMOD3, TMOD2	90	22	20655	20.86364	0.999926	1	1
BGE	GOTERM_BP_DIRECT	GO:1902176~negative regulation of oxidative stress-induced intrinsic apoptotic signaling pathway	2	6.666667	0.006703	BAG5, HSPB1	26	5	18600	286.1538	0.559803	0.811076	0.811076
BGE	GOTERM_CC_DIRECT	GO:0005737~cytoplasm	11	36.66667	0.010543	BAG5, MDH2, SNTG1, GRK7, DPYD, HSPB1, DTX2, ATP1B3, KLC1, MARK3, YWHAG	27	3681	20655	2.286064	0.392335	0.4955	0.4955
BGE	GOTERM_MF_DIRECT	GO:0042802~identical protein binding	4	13.33333	0.013075	TTR, PEAK1, HMG20A, YWHAG	27	371	19369	7.734451	0.57492	0.81934	0.81934
BGE	GOTERM_MF_DIRECT	GO:0005080~protein kinase C binding	2	6.666667	0.02521	HSPB1, YWHAG	27	19	19369	75.51267	0.809802	0.81934	0.81934
BGE	GOTERM_BP_DIRECT	GO:0045766~positive regulation of angiogenesis	2	6.666667	0.054983	CCL24, HSPB1	26	42	18600	34.06593	0.998992	1	1
BGE	GOTERM_BP_DIRECT	GO:0035556~intracellular signal transduction	3	10	0.05885	RASA2, HSPB1, MARK3	26	294	18600	7.299843	0.999388	1	1
BGE	GOTERM_MF_DIRECT	GO:0019904~protein domain specific binding	2	6.666667	0.07381	TFDP2, YWHAG	27	57	19369	25.17089	0.993153	1	1
BGE	GOTERM_BP_DIRECT	GO:0050821~protein stabilization	2	6.666667	0.076402	BAG5, ATP1B3	26	59	18600	24.25033	0.999938	1	1
BGE	GOTERM_BP_DIRECT	GO:0007030~Golgi organization	2	6.666667	0.097355	BAG5, TRAPPC8	26	76	18600	18.82591	0.999996	1	1
CME	KEGG_PATHWAY	oas04512:ECM-receptor interaction	3	14.28571	0.001305	SDC4, IBSP, MEPE	7	92	9691	45.14441	0.030843	0.032613	0.032613
CME	KEGG_PATHWAY	oas05145:Toxoplasmosis	2	9.52381	0.080168	CIITA, SOCS1	7	134	9691	20.66311	0.865413	1	1
EMZ	GOTERM_CC_DIRECT	GO:0005856~cytoskeleton	6	10.71429	0.000849	KITLG, PPP2R2B, TACC1, FRMD4B, TYK2, ARHGAP26	53	294	20655	7.953408	0.044842	0.027933	0.027933
EMZ	GOTERM_CC_DIRECT	GO:0005737~cytoplasm	20	35.71429	0.001035	CCNJ, PLEKHA2, ZNF518A, KEAP1, AK5, TYK2, ARHGAP26, ILF3, KITLG, TNKS2, CDC37, DPYSL3, BLNK, EIF3G, TACC1, SPDL1, DOCK2, CPEB3, SHFL, ATG4D	53	3681	20655	2.117452	0.054362	0.027933	0.027933

EMZ	GOTERM_MF_DIRECT	GO:0005178~integrin binding	4	7.142857	0.001112	ADAM9, ICAM4, ICAM5, ICAM1	48	84	19369	19.21528	0.095267	0.10006	0.10006
EMZ	GOTERM_BP_DIRECT	GO:0046427~positive regulation of receptor signaling pathway via JAK-STAT	3	5.357143	0.002265	PRP2, PRP1, TYK2	48	28	18600	41.51786	0.243434	0.276384	0.276384
EMZ	GOTERM_MF_DIRECT	GO:0038036~sphingosine-1-phosphate receptor activity	2	3.571429	0.016865	S1PR2, S1PR5	48	7	19369	115.2917	0.783643	0.758941	0.758941
EMZ	KEGG_PATHWAY	oas04151:PI3K-Akt signaling pathway	5	8.928571	0.017584	PRP2, KITLG, PPP2R2B, CDC37, PRP1	26	396	9691	4.706197	0.800995	1	1
EMZ	GOTERM_BP_DIRECT	GO:0008053~mitochondrial fusion	2	3.571429	0.034813	MIGA1, NEXN	48	14	18600	55.35714	0.9872	1	1
EMZ	GOTERM_BP_DIRECT	GO:0098609~cell-cell adhesion	3	5.357143	0.036951	ICAM4, ICAM5, ICAM1	48	120	18600	9.6875	0.990255	1	1
EMZ	GOTERM_MF_DIRECT	GO:0005148~prolactin receptor binding	2	3.571429	0.040477	PRP2, PRP1	48	17	19369	47.47304	0.975734	1	1
EMZ	KEGG_PATHWAY	oas04071:Sphingolipid signaling pathway	3	5.357143	0.042004	PPP2R2B, S1PR2, S1PR5	26	127	9691	8.804664	0.979859	1	1
EMZ	GOTERM_BP_DIRECT	GO:1903489~positive regulation of lactation	2	3.571429	0.042117	PRP2, PRP1	48	17	18600	45.58824	0.994972	1	1
EMZ	GOTERM_BP_DIRECT	GO:0031667~response to nutrient levels	2	3.571429	0.051772	PRP2, PRP1	48	21	18600	36.90476	0.998554	1	1
EMZ	GOTERM_BP_DIRECT	GO:0030879~mammary gland development	2	3.571429	0.056564	PRP2, PRP1	48	23	18600	33.69565	0.999224	1	1
EMZ	GOTERM_CC_DIRECT	GO:0005829~cytosol	9	16.07143	0.06092	CDKN2D, PPP2R2B, DNAJB4, DPYSL3, ANKRD12, BLNK, KEAP1, AK5, TYK2	53	1700	20655	2.063208	0.966431	0.888776	0.888776
EMZ	GOTERM_BP_DIRECT	GO:0044772~mitotic cell cycle phase transition	2	3.571429	0.061331	CCNJ, ZNF518A	48	25	18600	31	0.999584	1	1
EMZ	GOTERM_CC_DIRECT	GO:0000307~cyclin-dependent protein kinase holoenzyme complex	2	3.571429	0.065835	CCNJ, ZNF518A	53	27	20655	28.86792	0.974715	0.888776	0.888776
EMZ	GOTERM_BP_DIRECT	GO:0000079~regulation of cyclin-dependent protein serine/threonine kinase activity	2	3.571429	0.073148	CDKN2D, CCNJ	48	30	18600	25.83333	0.999912	1	1
EMZ	KEGG_PATHWAY	oas04080:Neuroactive ligand-receptor interaction	4	7.142857	0.074103	PRP2, PRP1, S1PR2, S1PR5	26	383	9691	3.89275	0.999094	1	1
EMZ	GOTERM_MF_DIRECT	GO:0051537~2 iron, 2 sulfur cluster binding	2	3.571429	0.074857	ANKRD12, NDUFV2	48	32	19369	25.22005	0.99909	1	1
EMZ	GOTERM_MF_DIRECT	GO:0016538~cyclin-dependent protein serine/threonine kinase regulator activity	2	3.571429	0.077105	CCNJ, ZNF518A	48	33	19369	24.45581	0.999269	1	1
EMZ	GOTERM_BP_DIRECT	GO:0008284~positive regulation of cell population proliferation	3	5.357143	0.078048	PRP2, KITLG, PRP1	48	183	18600	6.352459	0.999954	1	1
EMZ	GOTERM_MF_DIRECT	GO:0043022~ribosome binding	2	3.571429	0.097104	CPEB3, SHFL	48	42	19369	19.21528	0.999898	1	1
SUF	GOTERM_CC_DIRECT	GO:0030057~desmosome	6	7.692308	8.73E-10	DSG1, DSG3, DSG4, DSC1, DSC2, DSC3	73	14	20655	121.2622	6.11E-08	6.11E-08	6.11E-08
SUF	GOTERM_BP_DIRECT	GO:0007156~homophilic cell adhesion via plasma membrane adhesion molecules	7	8.974359	3.86E-06	DSG1, CDH15, DSG3, DSG4, DSC1, DSC2, DSC3	63	126	18600	16.40212	0.000765	0.000761	0.000761

SUF	GOTERM_MF_DIRECT	GO:0005509~calcium ion binding	9	11.53846	0.002488	RCN2, PLSCR3, DSG1, CDH15, DSG3, DSG4, DSC1, DSC2, DSC3	69	677	19369	3.731745	0.218582	0.246338	0.246338
SUF	GOTERM_BP_DIRECT	GO:0033693~neurofilament bundle assembly	2	2.564103	0.006656	NEFL, NEFM	63	2	18600	295.2381	0.733463	0.65559	0.65559
SUF	GOTERM_CC_DIRECT	GO:0099160~postsynaptic intermediate filament cytoskeleton	2	2.564103	0.010422	NEFL, NEFM	73	3	20655	188.6301	0.519698	0.364756	0.364756
SUF	GOTERM_MF_DIRECT	GO:0016165~linoleate 13S-lipoxygenase activity	2	2.564103	0.010496	ALOX15, ALOX12	69	3	19369	187.1401	0.648162	0.345766	0.345766
SUF	GOTERM_MF_DIRECT	GO:0050473~arachidonate 15-lipoxygenase activity	2	2.564103	0.01397	ALOX15, ALOX12	69	4	19369	140.3551	0.751625	0.345766	0.345766
SUF	GOTERM_MF_DIRECT	GO:0004052~arachidonate 12(S)-lipoxygenase activity	2	2.564103	0.01397	ALOX15, ALOX12	69	4	19369	140.3551	0.751625	0.345766	0.345766
SUF	GOTERM_CC_DIRECT	GO:0042383~sarcolemma	3	3.846154	0.019207	POPDC3, ALOX12, SLC2A4	73	61	20655	13.91534	0.742723	0.448175	0.448175
SUF	GOTERM_MF_DIRECT	GO:0045295~gamma-catenin binding	2	2.564103	0.020883	DSG1, DSC3	69	6	19369	93.57005	0.87623	0.413488	0.413488
SUF	GOTERM_BP_DIRECT	GO:0034440~lipid oxidation	2	2.564103	0.023105	ALOX15, ALOX12	63	7	18600	84.35374	0.99023	1	1
SUF	GOTERM_BP_DIRECT	GO:0043651~linoleic acid metabolic process	2	2.564103	0.026363	ALOX15, ALOX12	63	8	18600	73.80952	0.994957	1	1
SUF	GOTERM_BP_DIRECT	GO:0015718~monocarboxylic acid transport	2	2.564103	0.042491	SLC16A11, SLC16A13	63	13	18600	45.42125	0.999815	1	1
SUF	GOTERM_MF_DIRECT	GO:0008028~monocarboxylic acid transmembrane transporter activity	2	2.564103	0.051405	SLC16A11, SLC16A13	69	15	19369	37.42802	0.994617	0.821122	0.821122
SUF	GOTERM_BP_DIRECT	GO:1903489~positive regulation of lactation	2	2.564103	0.055204	PRP2, PRP1	63	17	18600	34.73389	0.999987	1	1
SUF	GOTERM_MF_DIRECT	GO:0005148~prolactin receptor binding	2	2.564103	0.058059	PRP2, PRP1	69	17	19369	33.02472	0.997319	0.821122	0.821122
SUF	GOTERM_BP_DIRECT	GO:0031667~response to nutrient levels	2	2.564103	0.06775	PRP2, PRP1	63	21	18600	28.11791	0.999999	1	1
SUF	GOTERM_CC_DIRECT	GO:0005829~cytosol	11	14.10256	0.069851	PLSCR3, ANKRD11, USP12, ALOX15, PREP, DVL2, ALOX12, SLC2A4, ELP5, GABARAP, SLC16A13	73	1700	20655	1.830822	0.99371	1	1
SUF	GOTERM_BP_DIRECT	GO:0030879~mammary gland development	2	2.564103	0.073962	PRP2, PRP1	63	23	18600	25.67288	1	1	1
SUF	GOTERM_BP_DIRECT	GO:0045197~establishment or maintenance of epithelial cell apical/basal polarity	2	2.564103	0.080133	CDX2, DLG4	63	25	18600	23.61905	1	1	1
SUF	GOTERM_MF_DIRECT	GO:0046872~metal ion binding	11	14.10256	0.080871	GALNS, PRP2, PAN3, ACAP1, FLT1, FLT3, PHF23, PRP1, GTF3A, CBFA2T3, LNX2	69	1738	19369	1.776646	0.999763	0.989873	0.989873
SUF	GOTERM_CC_DIRECT	GO:0005886~plasma membrane	18	23.07692	0.082364	DOCK5, FLT1, ALOX15, IRAK3, SLC2A4, GABARAP, PSTPIP1, PLSCR3, CLDN7, PIEZO1, DSG1, CDH15, DSG3, DSG4, DSC1, DSC2, LNX2, DSC3	73	3449	20655	1.476664	0.997563	1	1

SUF	GOTERM_BP_DIRECT	GO:0007275~multicellular organism development	3	3.846154	0.085318	FLT1, FLT3, CDH15	63	146	18600	6.066536	1	1	1
SUF	GOTERM_BP_DIRECT	GO:0046427~positive regulation of receptor signaling pathway via JAK-STAT	2	2.564103	0.089314	PRP2, PRP1	63	28	18600	21.08844	1	1	1
SUF	GOTERM_MF_DIRECT	GO:0022857~transmembrane transporter activity	3	3.846154	0.089988	SLC16A11, SLC22A31, SLC16A13	69	143	19369	5.889024	0.999912	0.989873	0.989873
TIB	GOTERM_CC_DIRECT	GO:0030424~axon	4	8.163265	0.003653	NEFL, NEFM, SLC8A1, SNCA	41	160	20655	12.59451	0.16109	0.13917	0.13917
TIB	GOTERM_BP_DIRECT	GO:0033693~neurofilament bundle assembly	2	4.081633	0.00494	NEFL, NEFM	47	2	18600	395.7447	0.479898	0.647173	0.647173
TIB	GOTERM_CC_DIRECT	GO:0099160~postsynaptic intermediate filament cytoskeleton	2	4.081633	0.005799	NEFL, NEFM	41	3	20655	335.8537	0.243574	0.13917	0.13917
TIB	GOTERM_MF_DIRECT	GO:0005347~ATP transmembrane transporter activity	2	4.081633	0.020972	SLC25A41, SLC25A23	42	10	19369	92.23333	0.773195	0.807043	0.807043
TIB	GOTERM_MF_DIRECT	GO:0046872~metal ion binding	9	18.36735	0.027234	PRP2, PPM1L, EYA1, PRP1, BPNT1, TUBB4A, SLC8A1, ZC3H15, SNCA	42	1738	19369	2.38809	0.855264	0.807043	0.807043
TIB	KEGG_PATHWAY	oas04060:Cytokine-cytokine receptor interaction	4	8.163265	0.035112	PRP2, CD70, PRP1, TNFSF9	22	338	9691	5.213018	0.869628	1	1
TIB	GOTERM_MF_DIRECT	GO:0005148~prolactin receptor binding	2	4.081633	0.035397	PRP2, PRP1	42	17	19369	54.2549	0.919756	0.807043	0.807043
TIB	GOTERM_BP_DIRECT	GO:1903489~positive regulation of lactation	2	4.081633	0.041239	PRP2, PRP1	47	17	18600	46.5582	0.996147	1	1
TIB	GOTERM_BP_DIRECT	GO:0031667~response to nutrient levels	2	4.081633	0.050698	PRP2, PRP1	47	21	18600	37.68997	0.998959	1	1
TIB	KEGG_PATHWAY	oas04151:PI3K-Akt signaling pathway	4	8.163265	0.052179	PRP2, PSPN, PRP1, ITGAV	22	396	9691	4.449495	0.95286	1	1
TIB	GOTERM_MF_DIRECT	GO:0005212~structural constituent of eye lens	2	4.081633	0.053638	CRYBB1, CRYBA4	42	26	19369	35.47436	0.978914	0.807043	0.807043
TIB	GOTERM_BP_DIRECT	GO:0030879~mammary gland development	2	4.081633	0.055393	PRP2, PRP1	47	23	18600	34.41258	0.999459	1	1
TIB	GOTERM_MF_DIRECT	GO:0005164~tumor necrosis factor receptor binding	2	4.081633	0.057646	CD70, TNFSF9	42	28	19369	32.94048	0.984333	0.807043	0.807043
TIB	GOTERM_BP_DIRECT	GO:0046427~positive regulation of receptor signaling pathway via JAK-STAT	2	4.081633	0.067031	PRP2, PRP1	47	28	18600	28.26748	0.999895	1	1
TIB	GOTERM_BP_DIRECT	GO:0002088~lens development in camera-type eye	2	4.081633	0.08309	CRYBB1, CRYBA4	47	35	18600	22.61398	0.999989	1	1
TIB	GOTERM_CC_DIRECT	GO:0005737~cytoplasm	12	24.4898	0.086385	DOCK5, EPRS1, EYA1, KHSRP, SRRD, RAB3GAP2, NEFL, NEFM, NMD3, ANXA10, TUBB4A, SNCA	41	3681	20655	1.642316	0.986919	1	1
TIB	KEGG_PATHWAY	oas00970:Aminoacyl-tRNA biosynthesis	2	4.081633	0.099118	EPRS1, IARS2	22	48	9691	18.35417	0.997393	1	1

Supplementary Table S4. Gene Ontology (GO) term comparison between DCMS and iHS analyses

GO_ID	Description
**Unique to both iHS and DCMS**	
GO:0000045	autophagosome assembly
GO:0003682	chromatin binding
GO:0032991	protein-containing complex
GO:0005615	extracellular space
GO:0048706	embryonic skeletal system development
GO:0000139	Golgi membrane
**Unique to DCMS**	
GO:0045030	G protein-coupled UTP receptor activity
GO:0007157	heterophilic cell-cell adhesion via plasma membrane cell adhesion molecules
GO:0050901	leukocyte tethering or rolling
GO:0070492	oligosaccharide binding
GO:0007219	Notch signaling pathway
GO:0034097	response to cytokine
GO:0033691	sialic acid binding
GO:0034237	protein kinase A regulatory subunit binding
GO:0016477	cell migration
GO:0005112	Notch binding
GO:0045177	apical part of cell
GO:1990349	gap junction-mediated intercellular transport
GO:0005922	connexin complex
GO:0007154	cell communication
GO:0016264	gap junction assembly
GO:1903763	gap junction channel activity involved in cell communication by electrical coupling
GO:0005243	gap junction channel activity
GO:0005515	protein binding
GO:0032590	dendrite membrane
**Unique to iHS**	
GO:0046849	bone remodeling
GO:0050955	thermoception
GO:0006874	cellular calcium ion homeostasis
GO:0009409	response to cold
GO:0016048	detection of temperature stimulus
GO:0051101	regulation of DNA binding
GO:0034080	CENP-A containing nucleosome assembly
GO:0007059	chromosome segregation
GO:0043254	regulation of protein complex assembly
GO:0046627	negative regulation of insulin receptor signaling pathway

GO:0090263	positive regulation of canonical Wnt signaling pathway
GO:0030837	negative regulation of actin filament polymerization
GO:0030336	negative regulation of cell migration
GO:1900025	negative regulation of substrate adhesion-dependent cell spreading
GO:0090521	glomerular visceral epithelial cell migration
GO:0008283	cell proliferation
GO:0010977	negative regulation of neuron projection development
GO:2000114	regulation of establishment of cell polarity
GO:0030036	actin cytoskeleton organization
GO:2000393	negative regulation of lamellipodium morphogenesis
GO:0035024	negative regulation of Rho protein signal transduction
GO:0090303	positive regulation of wound healing
GO:1900028	negative regulation of ruffle assembly
GO:0045835	negative regulation of meiotic nuclear division
GO:0060903	positive regulation of meiosis I
GO:0002176	male germ cell proliferation
GO:2000020	positive regulation of male gonad development
GO:0000902	cell morphogenesis
GO:0048599	oocyte development
GO:1900107	regulation of nodal signaling pathway
GO:0060009	Sertoli cell development
GO:0000122	negative regulation of transcription from RNA polymerase II promoter
GO:0007283	spermatogenesis
GO:0045840	positive regulation of mitotic nuclear division
GO:0008354	germ cell migration
GO:0030238	male sex determination
GO:0045944	positive regulation of transcription from RNA polymerase II promoter
GO:2000287	positive regulation of myotome development
GO:0014807	regulation of somitogenesis
GO:0046661	male sex differentiation
GO:0021521	ventral spinal cord interneuron specification
GO:0007628	adult walking behavior
GO:0042487	regulation of odontogenesis of dentin-containing tooth
GO:0019226	transmission of nerve impulse
GO:0006542	glutamine biosynthetic process
GO:0055085	transmembrane transport
GO:0043161	proteasome-mediated ubiquitin-dependent protein catabolic process
GO:0006974	cellular response to DNA damage stimulus
GO:0070233	negative regulation of T cell apoptotic process
GO:0007264	small GTPase mediated signal transduction
GO:1990869	cellular response to chemokine
GO:0061485	memory T cell proliferation
GO:0036336	dendritic cell migration
GO:1903905	positive regulation of establishment of T cell polarity

GO:2000406	positive regulation of T cell migration
GO:0043547	positive regulation of GTPase activity
GO:0001771	immunological synapse formation
GO:0043966	histone H3 acetylation
GO:0051457	maintenance of protein location in nucleus
GO:0019303	D-ribose catabolic process
GO:0006412	translation
GO:0006302	double-strand break repair
GO:0072425	signal transduction involved in G2 DNA damage checkpoint
GO:0043066	negative regulation of apoptotic process
GO:0045739	positive regulation of DNA repair
GO:0010212	response to ionizing radiation
GO:0007270	neuron-neuron synaptic transmission
GO:0006915	apoptotic process
GO:0001662	behavioral fear response
GO:0030855	epithelial cell differentiation
GO:0007612	learning
GO:2000378	negative regulation of reactive oxygen species metabolic process
GO:1903146	regulation of mitophagy
GO:0071353	cellular response to interleukin-4
GO:0042127	regulation of cell proliferation
GO:0030538	embryonic genitalia morphogenesis
GO:0048619	embryonic hindgut morphogenesis
GO:0044336	canonical Wnt signaling pathway involved in negative regulation of apoptotic process
GO:0048557	embryonic digestive tract morphogenesis
GO:0033153	T cell receptor V(D)J recombination
GO:0046632	alpha-beta T cell differentiation
GO:0021915	neural tube development
GO:0060070	canonical Wnt signaling pathway
GO:0006357	regulation of transcription from RNA polymerase II promoter
GO:0016055	Wnt signaling pathway
GO:0006511	ubiquitin-dependent protein catabolic process
GO:0051026	chiasma assembly
GO:0051865	protein autoubiquitination
GO:0050821	protein stabilization
GO:0033522	histone H2A ubiquitination
GO:0010845	positive regulation of reciprocal meiotic recombination
GO:0070193	synaptonemal complex organization
GO:0006344	maintenance of chromatin silencing
GO:0070979	protein K11-linked ubiquitination
GO:0070534	protein K63-linked ubiquitination
GO:0006513	protein monoubiquitination
GO:0006301	postreplication repair
GO:0070936	protein K48-linked ubiquitination
GO:0007288	sperm axoneme assembly

GO:0043951	negative regulation of cAMP-mediated signaling
GO:0042493	response to drug
GO:0070076	histone lysine demethylation
GO:0001701	in utero embryonic development
GO:0045141	meiotic telomere clustering
GO:0033128	negative regulation of histone phosphorylation
GO:0009411	response to UV
GO:0060395	SMAD protein signal transduction
GO:0050769	positive regulation of neurogenesis
GO:0000209	protein polyubiquitination
GO:1990138	neuron projection extension
GO:0090110	cargo loading into COPII-coated vesicle
GO:0006886	intracellular protein transport
GO:0045714	regulation of low-density lipoprotein particle receptor biosynthetic process
GO:0050714	positive regulation of protein secretion
GO:0042632	cholesterol homeostasis
GO:0016192	vesicle-mediated transport
GO:0008104	protein localization
GO:0008344	adult locomotory behavior
GO:0090103	cochlea morphogenesis
GO:0007189	adenylate cyclase-activating G-protein coupled receptor signaling pathway
GO:0030183	B cell differentiation
GO:0040018	positive regulation of multicellular organism growth
GO:1904588	cellular response to glycoprotein
GO:0071542	dopaminergic neuron differentiation
GO:0038194	thyroid-stimulating hormone signaling pathway
GO:0060122	inner ear receptor stereocilium organization
GO:1905229	cellular response to thyrotropin-releasing hormone
GO:0040012	regulation of locomotion
GO:0007166	cell surface receptor signaling pathway
GO:0006367	transcription initiation from RNA polymerase II promoter
GO:0016579	protein deubiquitination
GO:0015701	bicarbonate transport
GO:0034642	mitochondrion migration along actin filament
GO:0032465	regulation of cytokinesis
GO:0090140	regulation of mitochondrial fission
GO:0006506	GPI anchor biosynthetic process
GO:0072659	protein localization to plasma membrane
GO:0006694	steroid biosynthetic process
GO:0006396	RNA processing
GO:0008285	negative regulation of cell proliferation
GO:0060716	labyrinthine layer blood vessel development
GO:0010629	negative regulation of gene expression
GO:0030154	cell differentiation

GO:0033140	negative regulation of peptidyl-serine phosphorylation of STAT protein
GO:0061099	negative regulation of protein tyrosine kinase activity
GO:0042532	negative regulation of tyrosine phosphorylation of STAT protein
GO:0007420	brain development
GO:0001525	angiogenesis
GO:0051781	positive regulation of cell division
GO:1903672	positive regulation of sprouting angiogenesis
GO:0045766	positive regulation of angiogenesis
GO:0001658	branching involved in ureteric bud morphogenesis
GO:0008543	fibroblast growth factor receptor signaling pathway
GO:0090050	positive regulation of cell migration involved in sprouting angiogenesis
GO:0043536	positive regulation of blood vessel endothelial cell migration
GO:0060548	negative regulation of cell death
GO:0045786	negative regulation of cell cycle
GO:0006955	immune response
GO:0048469	cell maturation
GO:0045954	positive regulation of natural killer cell mediated cytotoxicity
GO:0032740	positive regulation of interleukin-17 production
GO:0007260	tyrosine phosphorylation of STAT protein
GO:0042531	positive regulation of tyrosine phosphorylation of STAT protein
GO:0042102	positive regulation of T cell proliferation
GO:0030890	positive regulation of B cell proliferation
GO:0002250	adaptive immune response
GO:0002366	leukocyte activation involved in immune response
GO:0002903	negative regulation of B cell apoptotic process
GO:0042104	positive regulation of activated T cell proliferation
GO:0007286	spermatid development
GO:0015914	phospholipid transport
GO:0042593	glucose homeostasis
GO:0035264	multicellular organism growth
GO:0002023	reduction of food intake in response to dietary excess
GO:0009749	response to glucose
GO:0046628	positive regulation of insulin receptor signaling pathway
GO:0007165	signal transduction
GO:0035973	aggrephagy
GO:1902175	regulation of oxidative stress-induced intrinsic apoptotic signaling pathway
GO:0071456	cellular response to hypoxia
GO:0034140	negative regulation of toll-like receptor 3 signaling pathway
GO:1901340	negative regulation of store-operated calcium channel activity
GO:0097352	autophagosome maturation
GO:0031398	positive regulation of protein ubiquitination
GO:0030433	ubiquitin-dependent ERAD pathway

GO:1903071	positive regulation of ER-associated ubiquitin-dependent protein catabolic process
GO:0046177	D-gluconate catabolic process
GO:0060271	cilium assembly
GO:0021591	ventricular system development
GO:0003351	epithelial cilium movement
GO:0032088	negative regulation of NF-kappaB transcription factor activity
GO:1902306	negative regulation of sodium ion transmembrane transport
GO:2000009	negative regulation of protein localization to cell surface
GO:0055070	copper ion homeostasis
GO:0048227	plasma membrane to endosome transport
GO:0006893	Golgi to plasma membrane transport
GO:0006289	nucleotide-excision repair
GO:0032434	regulation of proteasomal ubiquitin-dependent protein catabolic process
GO:1901985	positive regulation of protein acetylation
GO:0010824	regulation of centrosome duplication
GO:0046825	regulation of protein export from nucleus
GO:0034504	protein localization to nucleus
GO:0042176	regulation of protein catabolic process
GO:0000055	ribosomal large subunit export from nucleus
GO:0000056	ribosomal small subunit export from nucleus
GO:0051087	chaperone binding
GO:0006457	protein folding
GO:1904158	axonemal central apparatus assembly
GO:0033617	mitochondrial respiratory chain complex IV assembly
GO:0051881	regulation of mitochondrial membrane potential
GO:0006839	mitochondrial transport
GO:0006631	fatty acid metabolic process
GO:0000303	response to superoxide
GO:0030162	regulation of proteolysis
GO:0021997	neural plate axis specification
GO:0061511	centriole elongation
GO:0001947	heart looping
GO:1905515	non-motile cilium assembly
GO:0071539	protein localization to centrosome
GO:0008589	regulation of smoothened signaling pathway
GO:0042733	embryonic digit morphogenesis
GO:0016485	protein processing
GO:0006482	protein demethylation
GO:0005975	carbohydrate metabolic process
GO:0015459	potassium channel regulator activity
GO:0005249	voltage-gated potassium channel activity
GO:0033819	lipoyl(octanoyl) transferase activity
GO:1904894	positive regulation of STAT cascade
GO:0030335	positive regulation of cell migration
GO:0018279	protein N-linked glycosylation via asparagine

GO:1903614	negative regulation of protein tyrosine phosphatase activity
GO:0035556	intracellular signal transduction
GO:0098869	cellular oxidant detoxification
GO:1901031	regulation of response to reactive oxygen species
GO:0034198	cellular response to amino acid starvation
GO:0072593	reactive oxygen species metabolic process
GO:1904262	negative regulation of TORC1 signaling
GO:0042149	cellular response to glucose starvation
GO:0007040	lysosome organization
GO:0006685	sphingomyelin catabolic process
GO:0009143	nucleoside triphosphate catabolic process
GO:0018401	peptidyl-proline hydroxylation to 4-hydroxy-L-proline
GO:0006919	activation of cysteine-type endopeptidase activity involved in apoptotic process
GO:0001666	response to hypoxia
GO:0008360	regulation of cell shape
GO:0015721	bile acid and bile salt transport
GO:0071356	cellular response to tumor necrosis factor
GO:0043123	positive regulation of I-kappaB kinase/NF-kappaB signaling
GO:1902741	positive regulation of interferon-alpha secretion
GO:0034614	cellular response to reactive oxygen species
GO:0071276	cellular response to cadmium ion
GO:0098586	cellular response to virus
GO:0038061	NIK/NF-kappaB signaling
GO:0009415	response to water
GO:0071468	cellular response to acidic pH
GO:0007224	smoothened signaling pathway
GO:0051289	protein homotetramerization
GO:0032418	lysosome localization
GO:0048490	anterograde synaptic vesicle transport
GO:0008625	extrinsic apoptotic signaling pathway via death domain receptors
GO:0097345	mitochondrial outer membrane permeabilization
GO:0007275	multicellular organism development
GO:0006636	unsaturated fatty acid biosynthetic process
GO:0006633	fatty acid biosynthetic process
GO:0045717	negative regulation of fatty acid biosynthetic process
GO:0032933	SREBP signaling pathway
GO:0045541	negative regulation of cholesterol biosynthetic process
GO:0005576	extracellular region
GO:0009897	external side of plasma membrane
GO:0016021	integral component of membrane
GO:0005623	cell
GO:0005730	nucleolus
GO:0005654	nucleoplasm
GO:0000777	condensed chromosome kinetochore
GO:0005739	mitochondrion

GO:0005737	cytoplasm
GO:0032587	ruffle membrane
GO:0005634	nucleus
GO:0005789	endoplasmic reticulum membrane
GO:0010008	endosome membrane
GO:0005765	lysosomal membrane
GO:0033162	melanosome membrane
GO:0005829	cytosol
GO:0005886	plasma membrane
GO:0031256	leading edge membrane
GO:0030914	STAGA complex
GO:0005840	ribosome
GO:0070552	BRISC complex
GO:0070531	BRCA1-A complex
GO:0000152	nuclear ubiquitin ligase complex
GO:0005741	mitochondrial outer membrane
GO:0045202	synapse
GO:0005757	mitochondrial permeability transition pore complex
GO:0042645	mitochondrial nucleoid
GO:0005719	nuclear euchromatin
GO:0016604	nuclear body
GO:0033503	HULC complex
GO:0005657	replication fork
GO:0000790	nuclear chromatin
GO:0001741	XY body
GO:0030127	COPII vesicle coat
GO:0005794	Golgi apparatus
GO:0005783	endoplasmic reticulum
GO:0005814	centriole
GO:0000922	spindle pole
GO:0016323	basolateral plasma membrane
GO:0005672	transcription factor TFIIA complex
GO:0005669	transcription factor TFIID complex
GO:0030659	cytoplasmic vesicle membrane
GO:0005793	endoplasmic reticulum-Golgi intermediate compartment
GO:0005791	rough endoplasmic reticulum
GO:0030658	transport vesicle membrane
GO:0016324	apical plasma membrane
GO:0070062	extracellular exosome
GO:0031526	brush border membrane
GO:0005802	trans-Golgi network
GO:0048471	perinuclear region of cytoplasm
GO:0030667	secretory granule membrane
GO:0031362	anchored component of external side of plasma membrane
GO:0016459	myosin complex
GO:0036064	ciliary basal body

GO:0016235	aggresome
GO:0005776	autophagosome
GO:0005856	cytoskeleton
GO:0005929	cilium
GO:0055037	recycling endosome
GO:0005769	early endosome
GO:0031462	Cul2-RING ubiquitin ligase complex
GO:0097431	mitotic spindle pole
GO:0001917	photoreceptor inner segment
GO:0000235	astral microtubule
GO:0032391	photoreceptor connecting cilium
GO:0005813	centrosome
GO:0005642	annulate lamellae
GO:1990904	ribonucleoprotein complex
GO:0000776	kinetochore
GO:0031965	nuclear membrane
GO:0005761	mitochondrial ribosome
GO:0005762	mitochondrial large ribosomal subunit
GO:0097224	sperm connecting piece
GO:0005930	axoneme
GO:0005758	mitochondrial intermembrane space
GO:0031966	mitochondrial membrane
GO:0034451	centriolar satellite
GO:0016020	membrane
GO:0005923	bicellular tight junction
GO:0001650	fibrillar center
GO:0061700	GATOR2 complex
GO:0030136	clathrin-coated vesicle
GO:0005768	endosome
GO:0005911	cell-cell junction
GO:0005887	integral component of plasma membrane
GO:0009986	cell surface
GO:0046581	intercellular canaliculus
GO:0097730	non-motile cilium
GO:0043235	receptor complex
GO:0034704	calcium channel complex
GO:0031083	BLOC-1 complex
GO:0099078	BORC complex
GO:1904115	axon cytoplasm
GO:0000930	gamma-tubulin complex
GO:0005262	calcium channel activity
GO:0042802	identical protein binding
GO:0042393	histone binding
GO:0008013	beta-catenin binding
GO:0000987	core promoter proximal region sequence-specific DNA binding

GO:0046872	metal ion binding
GO:0001228	transcriptional activator activity, RNA polymerase II transcription regulatory region sequence-specific binding
GO:0000977	RNA polymerase II regulatory region sequence-specific DNA binding
GO:0003700	transcription factor activity, sequence-specific DNA binding
GO:0043565	sequence-specific DNA binding
GO:0004356	glutamate-ammonia ligase activity
GO:0031625	ubiquitin protein ligase binding
GO:0008270	zinc ion binding
GO:0032183	SUMO binding
GO:0004842	ubiquitin-protein transferase activity
GO:0005085	guanyl-nucleotide exchange factor activity
GO:0003676	nucleic acid binding
GO:0005525	GTP binding
GO:0003924	GTPase activity
GO:0003713	transcription coactivator activity
GO:0046982	protein heterodimerization activity
GO:0004402	histone acetyltransferase activity
GO:0005524	ATP binding
GO:0004747	ribokinase activity
GO:0003735	structural constituent of ribosome
GO:0031593	polyubiquitin binding
GO:0005164	tumor necrosis factor receptor binding
GO:0008308	voltage-gated anion channel activity
GO:0019901	protein kinase binding
GO:0044325	ion channel binding
GO:0001227	transcriptional repressor activity, RNA polymerase II transcription regulatory region sequence-specific binding
GO:0000978	RNA polymerase II core promoter proximal region sequence-specific DNA binding
GO:0003677	DNA binding
GO:0004721	phosphoprotein phosphatase activity
GO:0004672	protein kinase activity
GO:0061631	ubiquitin conjugating enzyme activity
GO:0004022	alcohol dehydrogenase (NAD) activity
GO:0061630	ubiquitin protein ligase activity
GO:0004996	thyroid-stimulating hormone receptor activity
GO:0044877	macromolecular complex binding
GO:0038023	signaling receptor activity
GO:0017025	TBP-class protein binding
GO:0016251	obsolete general RNA polymerase II transcription factor activity
GO:0001103	RNA polymerase II repressing transcription factor binding
GO:0004843	thiol-dependent ubiquitin-specific protease activity
GO:0005509	calcium ion binding
GO:0004089	carbonate dehydratase activity
GO:0003779	actin binding
GO:0060002	plus-end directed microfilament motor activity

GO:0032027	myosin light chain binding
GO:0016887	ATPase activity
GO:0016746	transferase activity, transferring acyl groups
GO:0000253	3-keto sterol reductase activity
GO:0004303	estradiol 17-beta-dehydrogenase activity
GO:0072582	17-beta-hydroxysteroid dehydrogenase (NADP+) activity
GO:0072555	17-beta-ketosteroid reductase activity
GO:0003723	RNA binding
GO:0008173	RNA methyltransferase activity
GO:0005178	integrin binding
GO:0005104	fibroblast growth factor receptor binding
GO:0008083	growth factor activity
GO:0008201	heparin binding
GO:0016787	hydrolase activity
GO:0031683	G-protein beta/gamma-subunit complex binding
GO:0032795	heterotrimeric G-protein binding
GO:0005134	interleukin-2 receptor binding
GO:0005125	cytokine activity
GO:0004000	adenosine deaminase activity
GO:0000287	magnesium ion binding
GO:0000166	nucleotide binding
GO:0019900	kinase binding
GO:0008092	cytoskeletal protein binding
GO:0046316	gluconokinase activity
GO:0008017	microtubule binding
GO:0003777	microtubule motor activity
GO:0080025	phosphatidylinositol-3,5-bisphosphate binding
GO:0042803	protein homodimerization activity
GO:0005507	copper ion binding
GO:0070300	phosphatidic acid binding
GO:0005547	phosphatidylinositol-3,4,5-trisphosphate binding
GO:0043325	phosphatidylinositol-3,4-bisphosphate binding
GO:0005546	phosphatidylinositol-4,5-bisphosphate binding
GO:0005049	nuclear export signal receptor activity
GO:0008536	Ran GTPase binding
GO:0051082	unfolded protein binding
GO:0017077	oxidative phosphorylation uncoupler activity
GO:0051722	protein C-terminal methylesterase activity
GO:0051721	protein phosphatase 2A binding
GO:0004656	procollagen-proline 4-dioxygenase activity
GO:0005506	iron ion binding
GO:0031418	L-ascorbic acid binding
GO:0016702	oxidoreductase activity, acting on single donors with incorporation of molecular oxygen, incorporation of two atoms of oxygen
GO:0016868	intramolecular transferase activity, phosphotransferases
GO:0047933	glucose-1,6-bisphosphate synthase activity

GO:0030145	manganese ion binding
GO:0030144	alpha-1,6-mannosylglycoprotein 6-beta-N-acetylglucosaminyltransferase activity
GO:0004864	protein phosphatase inhibitor activity
GO:0005096	GTPase activator activity
GO:0005198	structural molecule activity
GO:0000179	rRNA (adenine-N6,N6-)-dimethyltransferase activity
GO:0043015	gamma-tubulin binding
GO:0070728	leucine binding
GO:0004767	sphingomyelin phosphodiesterase activity
GO:0031545	peptidyl-proline 4-dioxygenase activity
GO:0005089	Rho guanyl-nucleotide exchange factor activity
GO:0042626	ATPase activity, coupled to transmembrane movement of substances
GO:0008514	organic anion transmembrane transporter activity
GO:0042910	xenobiotic transporter activity
GO:0004181	metallocarboxypeptidase activity
GO:0097110	scaffold protein binding
GO:0008384	IkappaB kinase activity
GO:0051371	muscle alpha-actinin binding
GO:0005272	sodium channel activity
GO:0033040	sour taste receptor activity
GO:0015269	calcium-activated potassium channel activity
GO:0048018	receptor agonist activity
GO:0004768	stearoyl-CoA 9-desaturase activity
GO:0016491	oxidoreductase activity
GO:0015485	cholesterol binding
