# Supplementary figures and images for: Comparative genomic insights into adaptation, selection signatures, and population dynamics in indigenous Indian sheep and foreign breeds
Source: Front Genet. 2025 Aug 21;16:1621960. doi: 10.3389/fgene.2025.1621960 (PMC12408274; doi:10.3389/fgene.2025.1621960)

# BGA

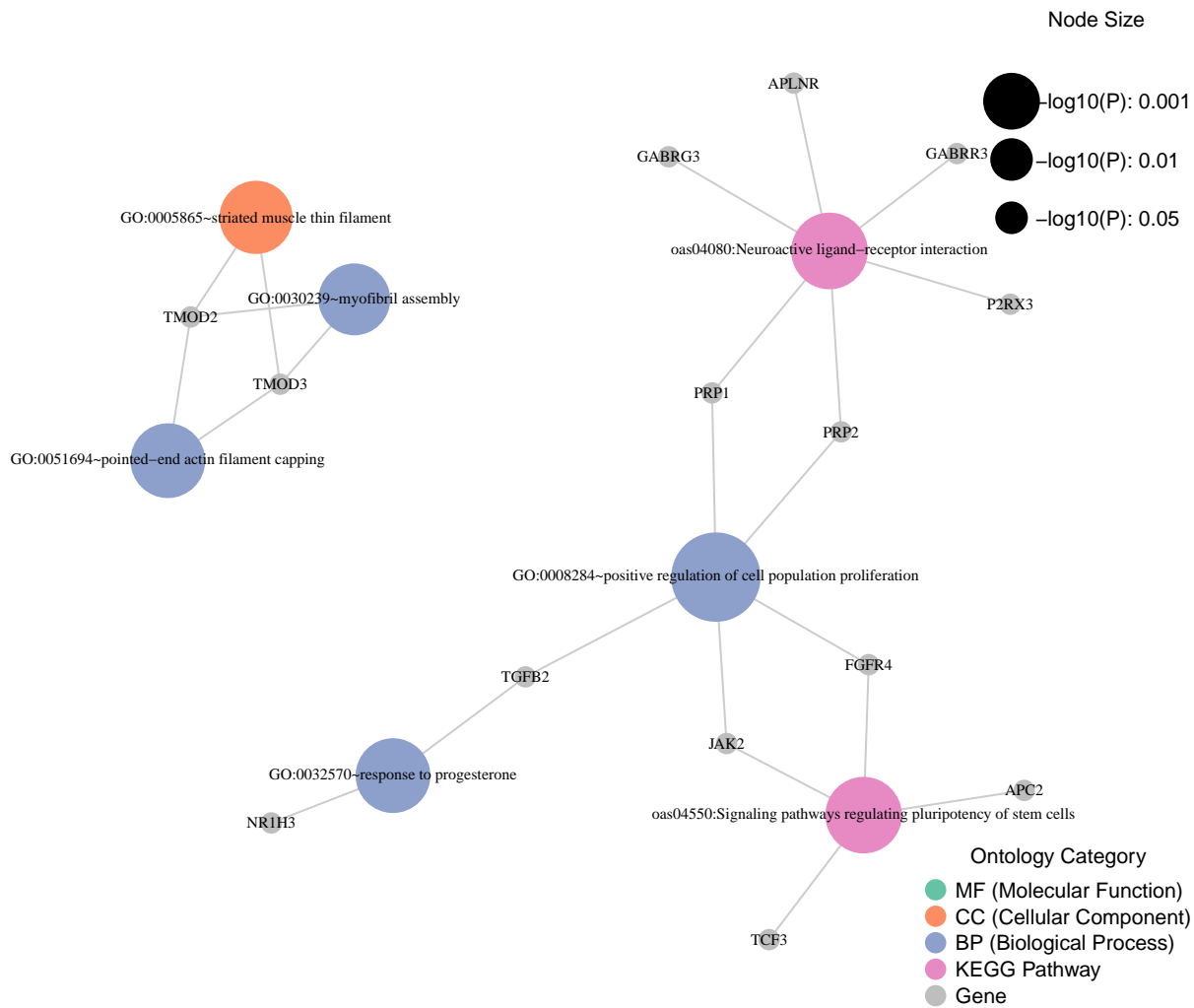

# BGE

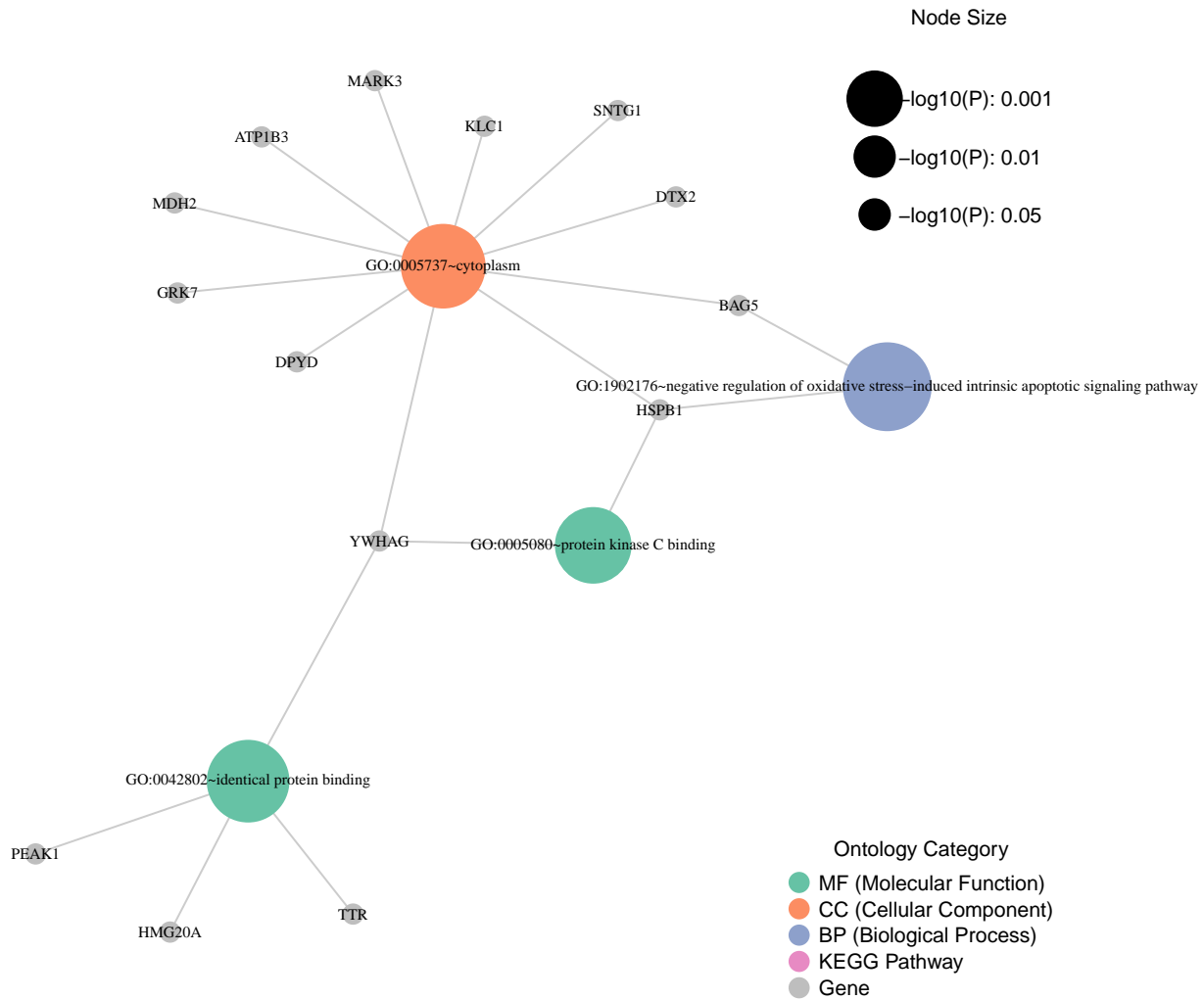

# CME

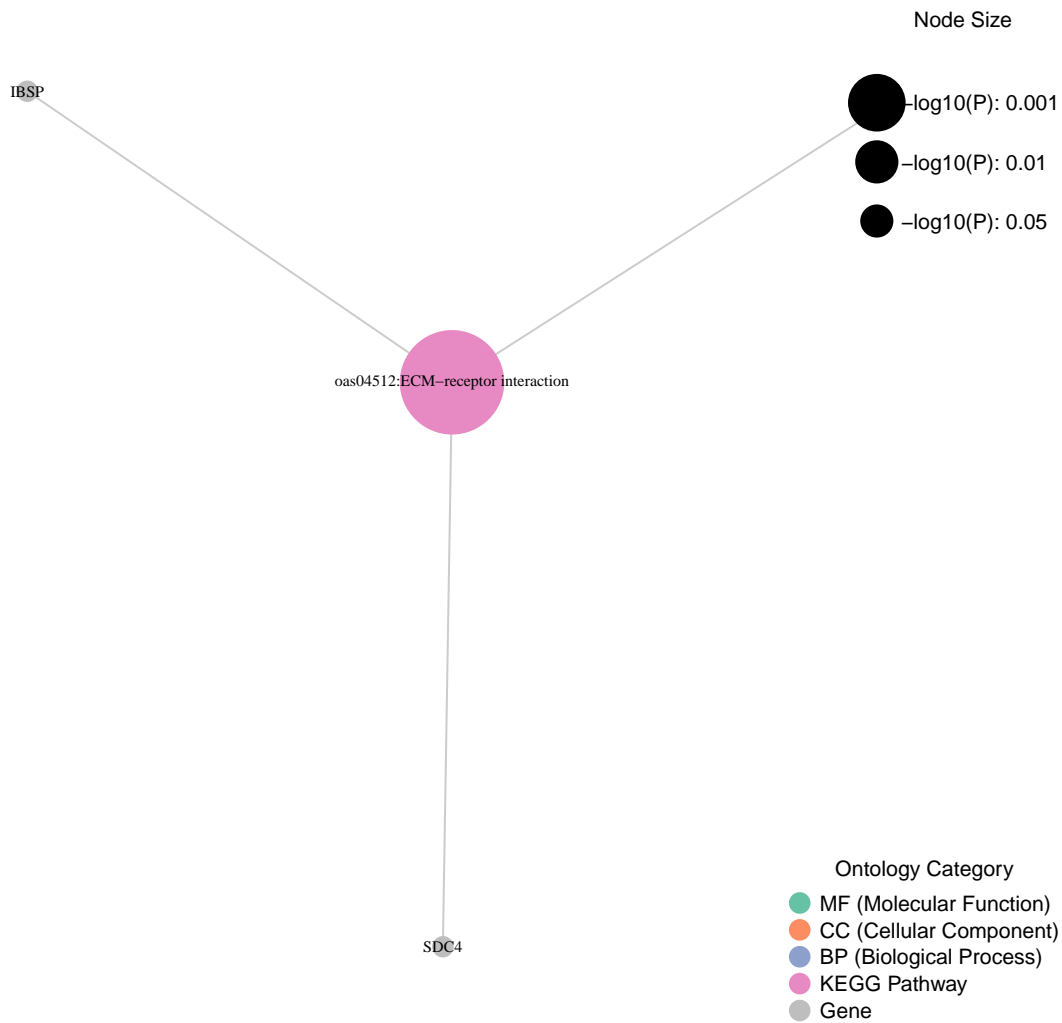

# EMZ

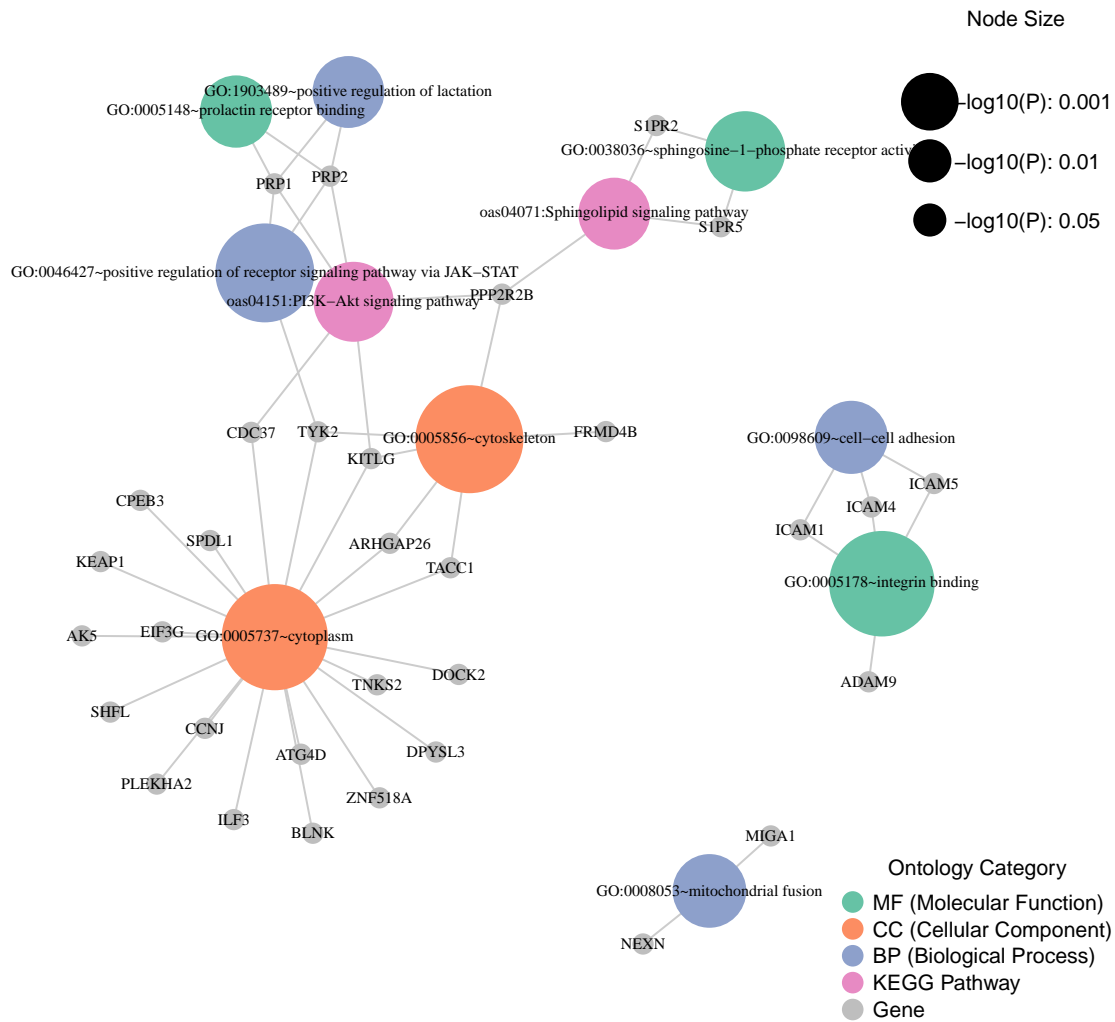

# IDC

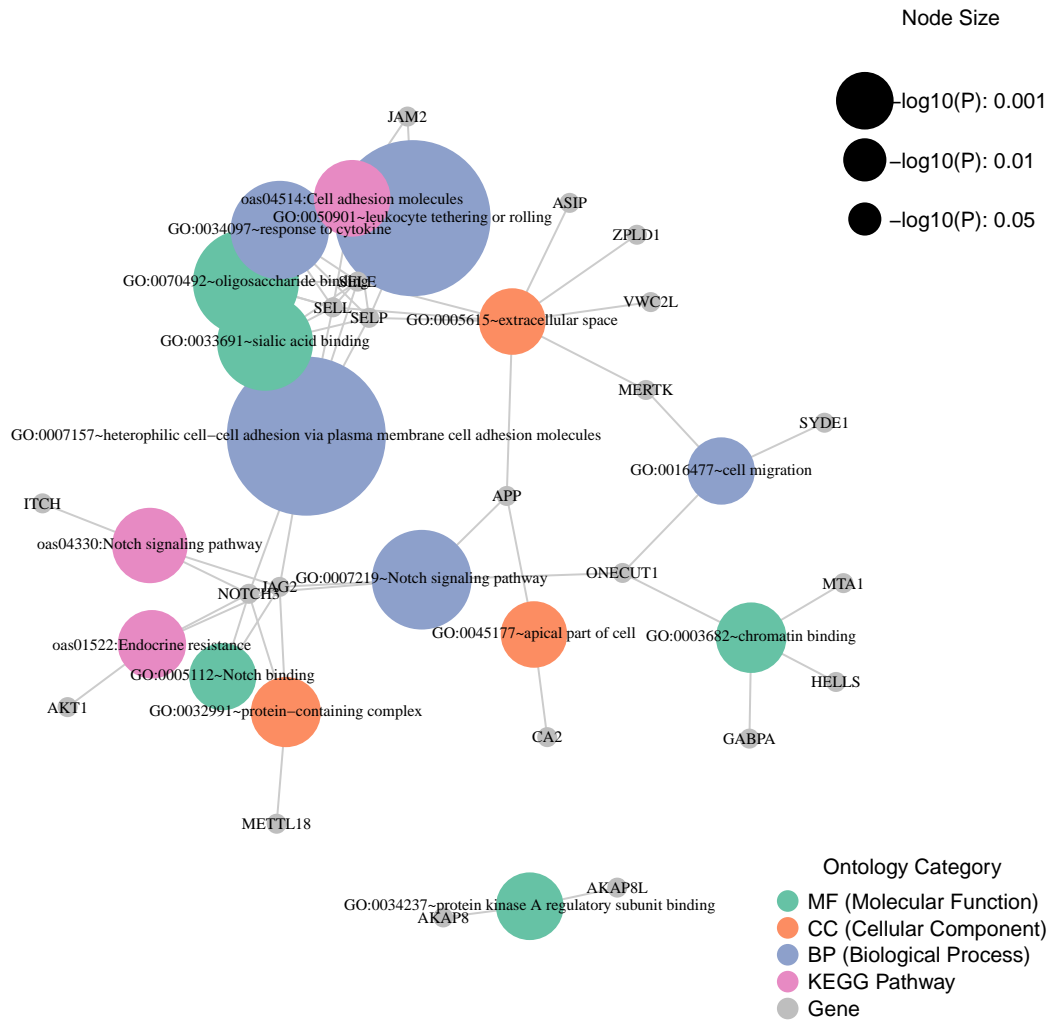

# SUF

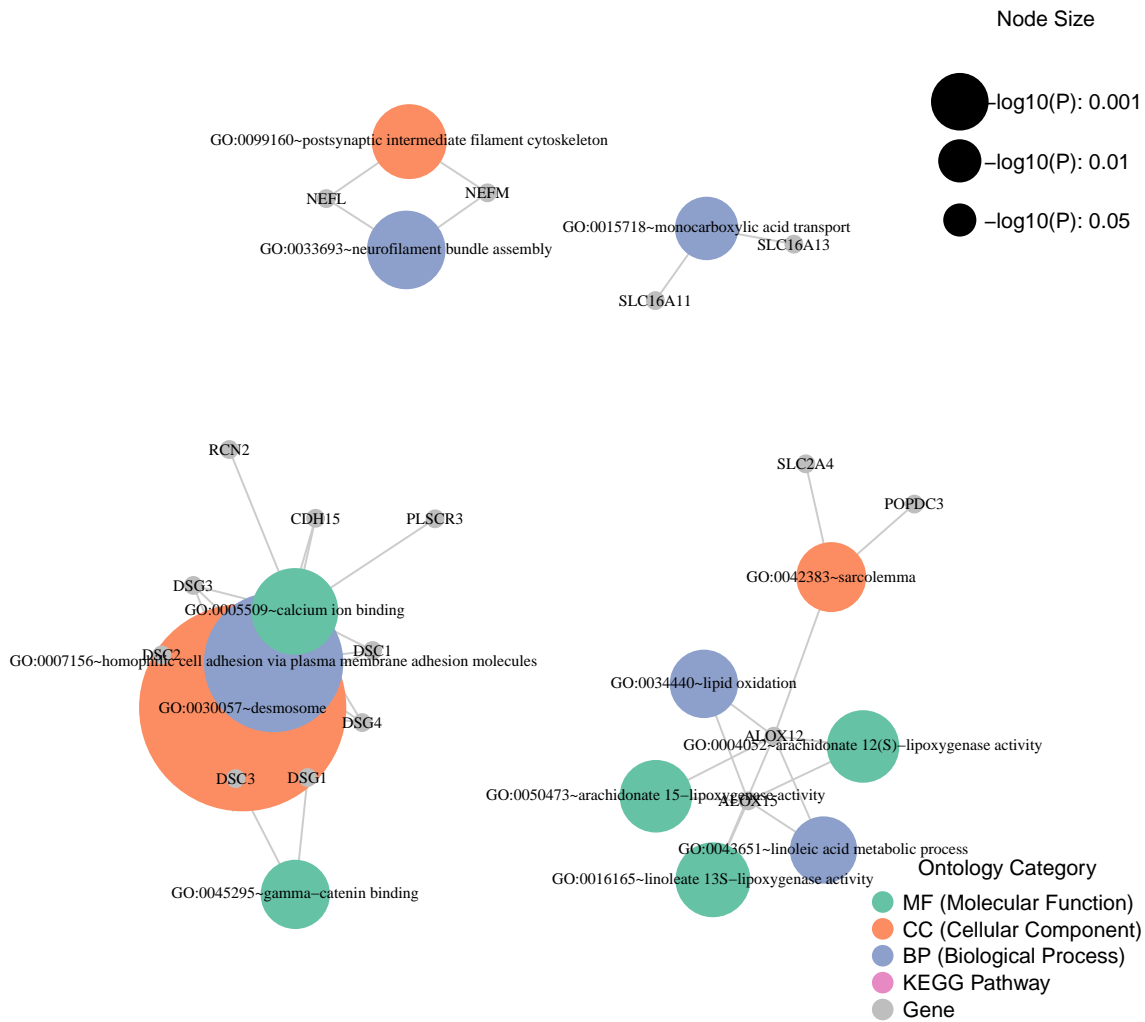

# TIB

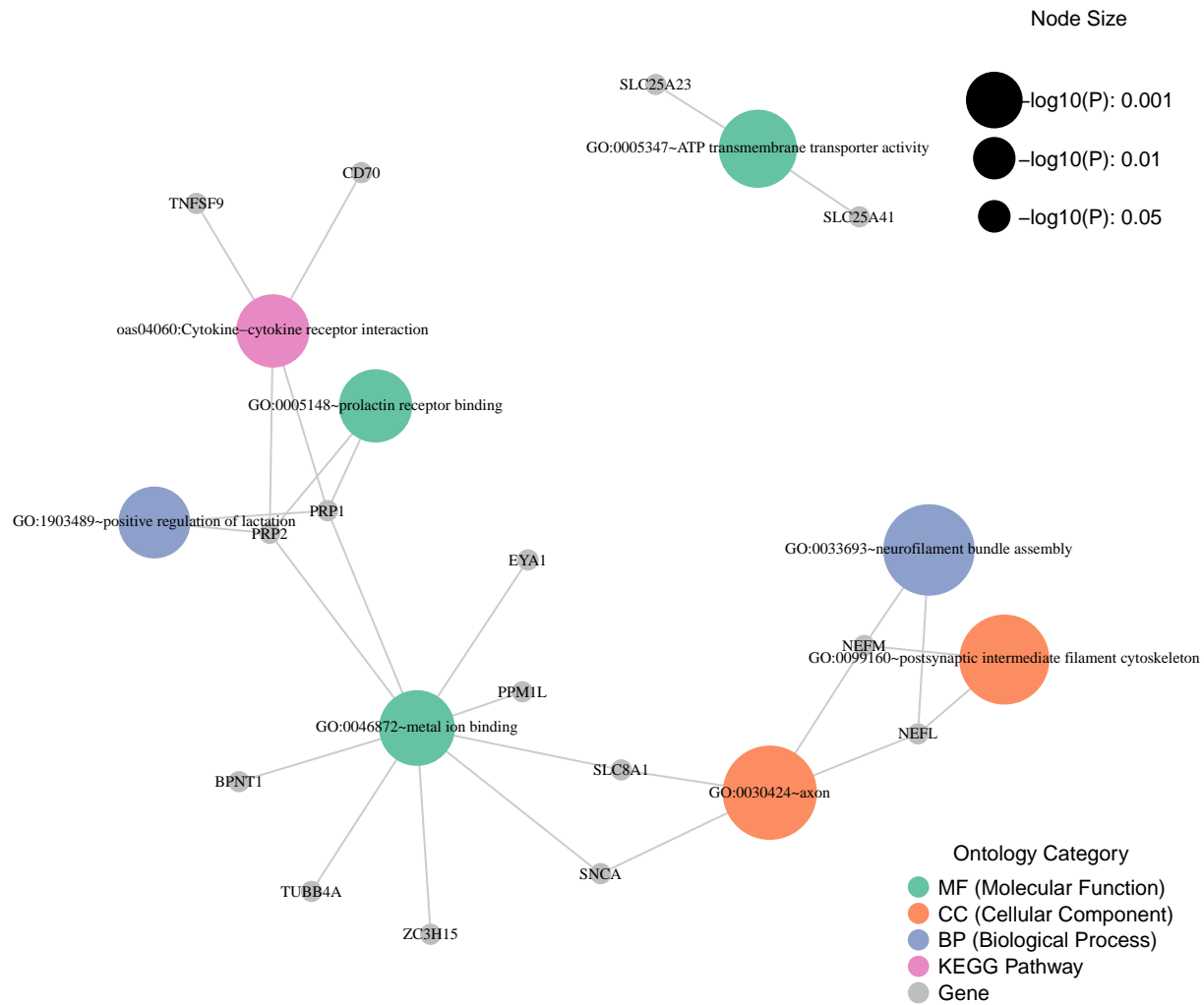

Supplement: Supplementary file 3 [file DataSheet1.pdf]
