# Supplementary material for: Comparative genomic insights into adaptation, selection signatures, and population dynamics in indigenous Indian sheep and foreign breeds
Source: Front Genet. 2025 Aug 21;16:1621960. doi: 10.3389/fgene.2025.1621960 (PMC12408274; doi:10.3389/fgene.2025.1621960)
Supplement: Supplementary file 1 [file DataSheet2.pdf]

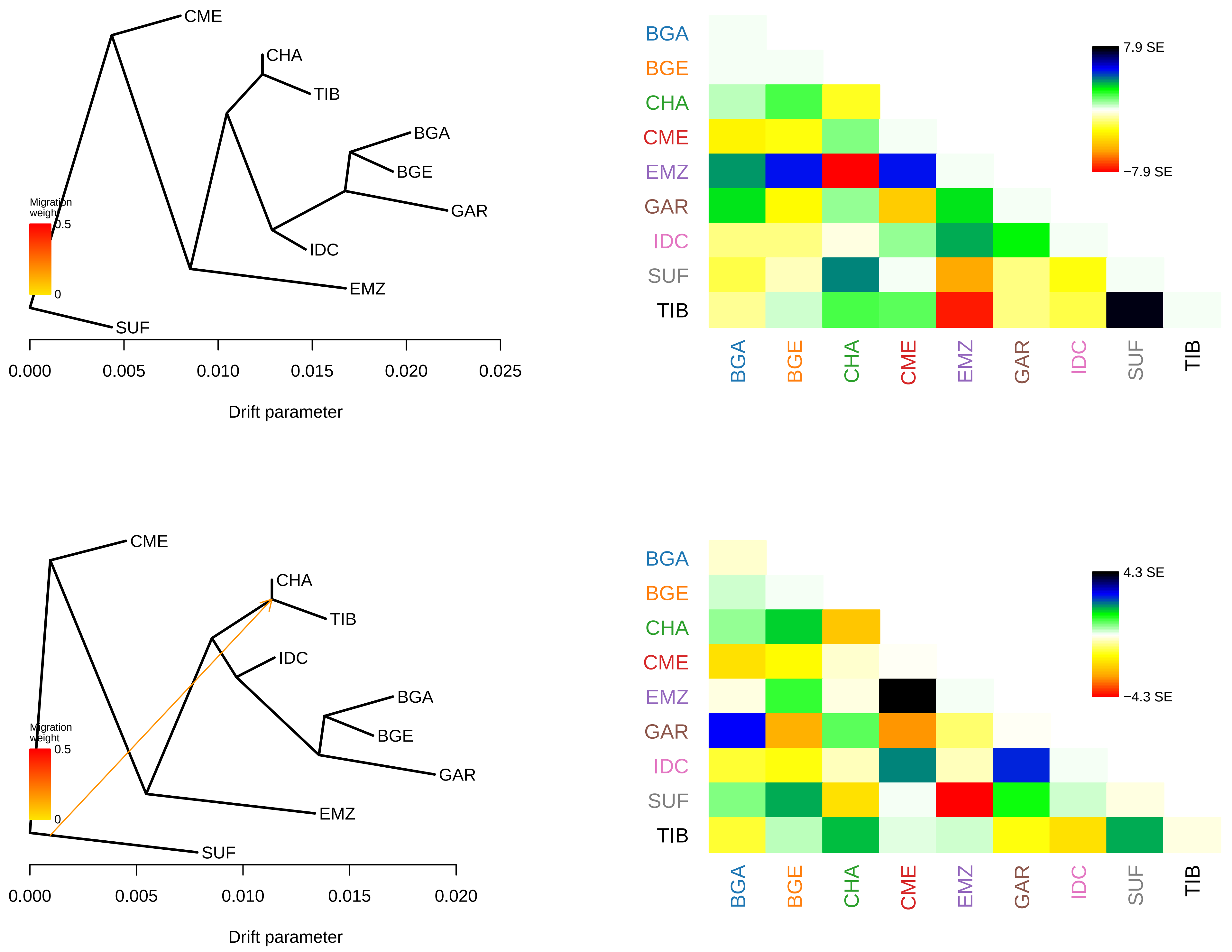

CME
CHA
TIB
BGA
BGE
GAR
IDC
EMZ
SUF
Migration weight
0.5
0
0.000
0.005
0.010
0.015
0.020
0.025
Drift parameter
BGA
BGE
CHA
CME
EMZ
GAR
IDC
SUF
TIB
7.9 SE
−7.9 SE
CME
CHA
TIB
IDC
BGA
BGE
GAR
EMZ
SUF
Migration weight
0.5
0
0.000
0.005
0.010
0.015
0.020
Drift parameter
BGA
BGE
CHA
CME
EMZ
GAR
IDC
SUF
TIB
4.3 SE
−4.3 SE

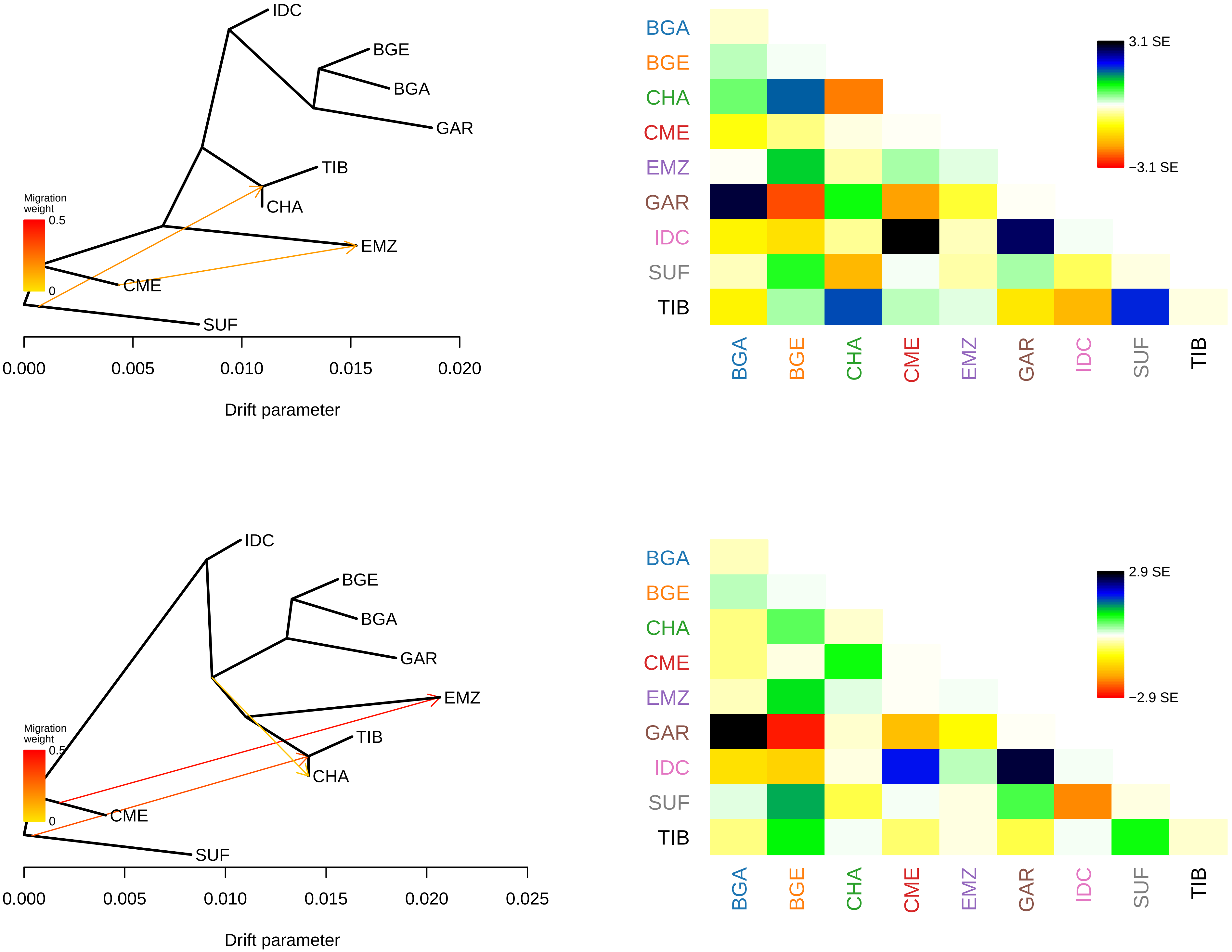
IDC
BGE
BGA
GAR
TIB
CHA
EMZ
CME
SUF
Migration weight
0.5
0
0.000
0.005
0.010
0.015
0.020
Drift parameter
BGA
BGE
CHA
CME
EMZ
GAR
IDC
SUF
TIB
3.1 SE
−3.1 SE
IDC
BGE
BGA
GAR
EMZ
TIB
CHA
CME
SUF
Migration weight
0.5
0
0.000
0.005
0.010
0.015
0.020
0.025
Drift parameter
BGA
BGE
CHA
CME
EMZ
GAR
IDC
SUF
TIB
2.9 SE
−2.9 SE

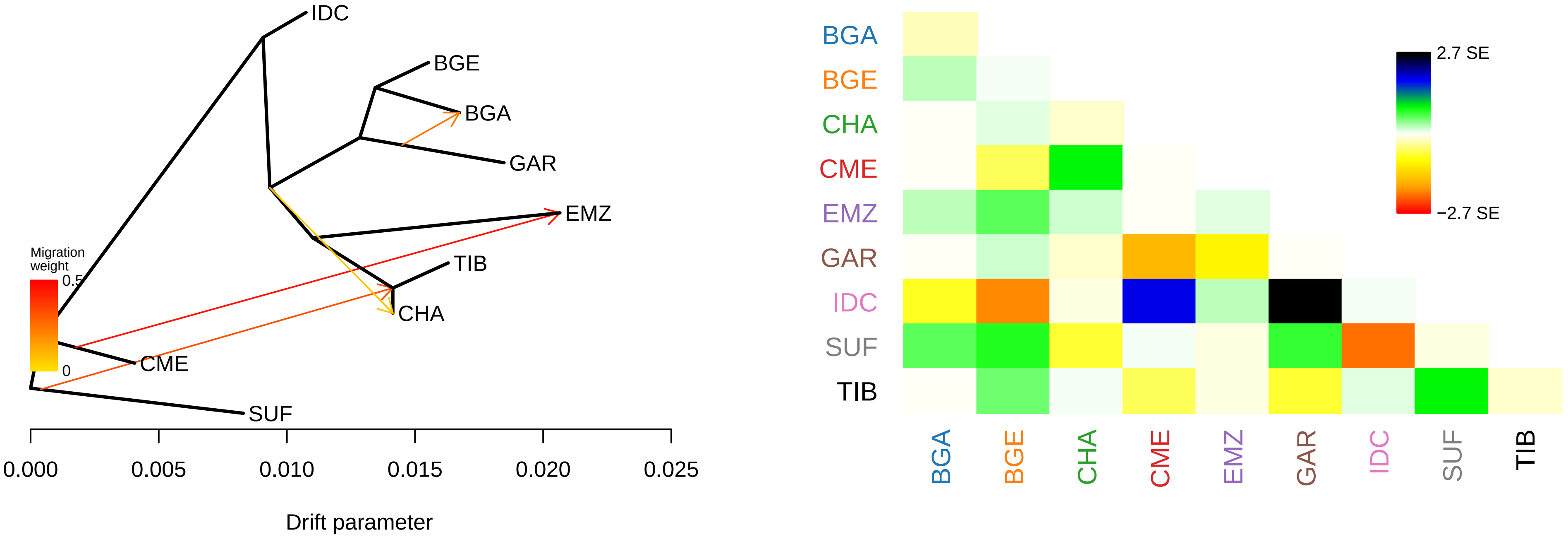
IDC
BGE
BGA
GAR
EMZ
TIB
CHA
CME
SUF
Migration weight
0.5
0
0.000
0.005
0.010
0.015
0.020
0.025
Drift parameter
BGA
BGE
CHA
CME
EMZ
GAR
IDC
SUF
TIB
2.7 SE
−2.7 SE
